# Single-Carbon Bridged Pentacene Dimers Enable Efficient Singlet Fission and Quintet State Stabilization

**DOI:** 10.1021/jacs.5c14851

**Published:** 2026-01-23

**Authors:** Chao-Hsien Hsu, Yi-Ching Liao, Chu-Chun Cheng, Bo-Han Wu, Chou-Hsun Yang, Chao-Ping Hsu, Bo-Han Chen, Shang-Da Yang, Yuling Hsu, Li-Kang Chu, Yun-Wei Chiang, Ken-Tsung Wong, Pi-Tai Chou

**Affiliations:** † Department of Chemistry, 33561 National Taiwan University, No. 1, Sec. 4, Roosevelt Rd., Taipei 106319, Taiwan; ‡ Department of Chemistry, 34881 National Tsing Hua University, Hsinchu 300044, Taiwan; § Institute of Chemistry, Academia Sinica, 128 Section 2 Academia Road, Nankang, Taipei 11529, Taiwan; ∥ Physics Division, National Center for Theoretical Sciences, Taipei 10617, Taiwan; ⊥ Institute of Photonics Technologies, National Tsing Hua University, Hsinchu 300044, Taiwan; # Institute of Atomic and Molecular Sciences, Academia Sinica, Taipei 10617, Taiwan

## Abstract

Singlet fission (SF) offers a promising avenue for quantum information science, as it generates spin-entangled triplet pairs with quintet character (^5^TT) upon photoexcitation, enabling access to multilevel spin qubit states beyond the traditional two-level systems. However, the ^5^TT state often decays via several pathways: (1) dissociation into isolated triplets; (2) triplet–triplet annihilation back into the singlet manifold; or (3) spin conversion to lower-multiplicity triplet pair states. These competing relaxation channels pose a major challenge for stabilizing ^5^TT. Here, we introduce a novel molecular design that prolongs ^5^TT lifetime by anchoring two pentacene chromophores to the same carbon (C9) position of a fluorene bridge, yielding **FlePc2** and **FlePhPc2**. This single-point attachment enforces a near-parallel intramolecular geometry, promoting strong through-space spin interactions that hinder dissociation. Field-swept electron spin echo (FS-ESE) measurements reveal dominant ^5^TT signals, indicative of suppressed relaxation pathways. Theoretical calculations predict a substantial binding energy for the reported dimers, accompanied by significant spin density delocalization across both pentacenes, thereby rationalizing ^5^TT stabilization. These findings establish a molecular design principle for kinetically trapping high-spin multiexciton states, paving the way for spin-based quantum technologies.

## Graphical abstract

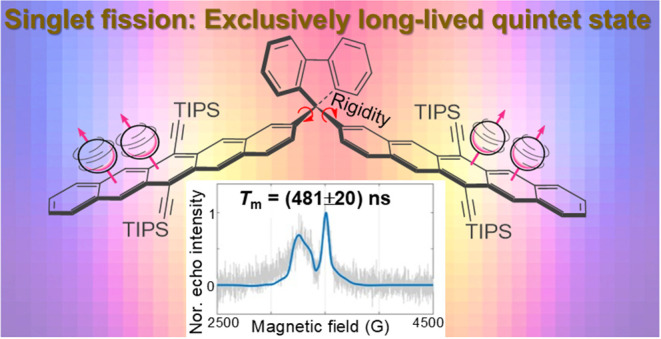

## Introduction

SF is a spin-allowed multiexciton generation process in which one singlet exciton splits into two triplet excitons.^1^ This phenomenon and theory were first proposed based on observations in anthracene crystals,^2^ and were then used to explain the low fluorescence quantum yield of tetracene crystals.^3^ The theory was subsequently confirmed in 1969 through studies of magnetic field effects.^4,5^ SF has attracted significant attention for its potential to break the Shockley–Queisser limit in photovoltaic devices by enhancing quantum efficiency.^6,7^ In recent years, molecular engineering strategies have enabled the realization of intramolecular SF in covalently linked chromophore dimers, offering improved solubility, structural tunability, and the potential to control spin-correlated intermediate states.^8–10^

Among the various intermediates formed during the SF process, the correlated triplet pair state (TT) can evolve into distinct spin multiplicities, including singlet (^1^TT), triplet (^3^TT), and ^5^TT configurations. Although these states may interconvert as the SF process evolves, their conversion pathways are governed by spin selection rules. Typically, ^1^TT can transform into ^5^TT via singlet–quintet mixing.^11–13^ However, subsequent spin conversion between ^5^TT and ^3^TT is generally forbidden by symmetry and magnetic dipolar considerations, due to the differing polarities of their wave functions.^1,14^ Consequently, the spin–orbit coupling (SOC) is required to promote further spin evolution. In addition to SOC, external perturbations, such as magnetic fields, can mitigate spin selection rules, thereby modulating the triplet yield.^15^

Nevertheless, triplet pairs with different spin multiplicities can dissociate into isolated triplets (T_1_) if the SF coupling weakens because of conformational flexibility.^16,17^ In this work, SF coupling refers to the strength of the electronic coupling between the photoexcited singlet state (S_1_S_0_) and the ^1^TT state, which governs the rate of SF according to Fermi’s golden rule. Details regarding the quantitative evaluation of SF coupling strength are described in the Theoretical Calculation section. Therefore, in the past, most studies focused on harvesting isolated T_1_ excitons for energy applications, as the dissociation process has more parameters to regulate, such as SF coupling,^8,17^ bridge linker,^18–21^ and the exciton migration length.^22–24^

Recently, the high-spin ^5^TT states have opened up a research avenue in the field of quantum information science.^25,26^ Since the ^5^TT state, composed of spin-correlated but spatially localized triplets, exhibits long coherence times and well-defined spin multiplicities, making it an excellent candidate for qubits and molecular spin logic.^27–30^ In 2023, Dill et al. first reported a rigid pentacene-based SF dimer that exhibited exclusive formation of the ^5^TT state, with no detectable lower-spin triplet intermediates.^31^ The absence of additional magnetically responsive intermediates such as ^3^TT or T_1_ excitons prevents state mixing and field-dependent spin precession, both of which would otherwise accelerate dephasing and shorten coherence lifetimes. However, the generation and stabilization of such high-spin multiexciton states remain challenging, posing significant hurdles for rational molecular design. The primary obstacles lie in precisely controlling both the electronic coupling and spatial orientation of the chromophore units to suppress undesired relaxation pathways. Consequently, maintaining a sufficiently close distance between the chromophores is essential to ensure substantial SF coupling.

Recent studies have shown that parallel and head-to-tail configurations of rigid pentacene dimers can possibly maintain the ^5^TT state even at room temperature.^28,29,32^ Within such molecular architectures, limited structural flexibility has been suggested as a key factor in enhancing the ^5^TT yield. In contrast to previous systems that commonly employ multiple σ-bridges or phenylene spacers,^21,31,33,34^ our design introduces a single-point fluorene linkage that connects two pentacene units through a single carbon atom (C9) of the fluorene core. This unique architecture enforces close spatial proximity and a well-defined, near-parallel intramolecular geometry while retaining only minimal but sufficient conformational freedom. The architecture promotes strong through-space orbital overlap while minimizing π-conjugation across the bridge and simultaneously simplifies the synthetic route, thereby reducing synthetic complexity and cost.

Importantly, this controlled rigidity effectively suppresses large-amplitude conformational motion and undesired relaxation pathways, yielding predominantly quintet-selective, long-lived EPR-active ^5^TT within the coherence-relevant regime. Such robust quintet-dominated spin selectivity is essential for coherent spin applications, as it minimizes state mixing and field-dependent spin precession, both of which would otherwise accelerate decoherence. The observed phase-memory times (*T*_m_) = 481 ± 20 ns at 80 K demonstrates that molecular rigidity and steric confinement can substantially mitigate decoherence, yielding a remarkably stable quintet even under thermally active conditions. Beyond maximizing absolute coherence times, the fluorene-bridge framework offers a complementary design strategy for stabilizing quintet states through controlled geometry and spin-density regulation. Electronic structure calculations using restricted active space double spin-flip (RAS-2SF) and density functional theory (DFT) further reveal strong spin–spin exchange coupling and clarify how subtle conformational fluctuations contribute to ^5^TT stabilization.

Our transient absorption (TA) and FS-ESE measurements reveal that this molecular architecture favors the generation of a long-lived ^5^TT state that dominates within the coherence-relevant time window, while substantially suppressing both the formation of dissociated T_1_ excitons and relaxation into the ^3^TT state. Notably, the stabilized ^5^TT state in FlePc2 persists without detectable decay up to 130 K, possibly near the glass transition temperature of the mixing solution, outperforming its structurally modified analogues. The observed stabilization of the ^5^TT state arises from a combination of geometric rigidity, suppressing the spin–orbit-induced mixing that would otherwise promote conversion to the ^3^TT state or separate into the T_1_ states. Therefore, the single-point fluorene attachment acts as a new design principle for SF-active molecules optimized not for charge harvesting, but for quantum coherent spin-state generation. Details of the results and discussion are elaborated in the following sections.

## Results and Discussion

### Molecular Design and Strategy

The syntheses and structures of molecules utilized in this study, **PhTIPSPc**, **FlePhPc2**, and **FlePc2**, are depicted in Scheme 1. In brief, **PhTIPSPc** was obtained via phenyl substitution of compound **2** via Suzuki coupling reaction. **FlePhPc2** was synthesized by the reaction of 9,9-bis­(4-iodophenyl)-9*H*-fluorene and **2**, catalyzed by Pd­(PPh_3_)_4_ under weak base conditions. **FlePc2** was obtained by the addition reaction of triisopropylsilyl (TIPS) acetylide with compound **6** in THF, followed by the treatment of tin­(II) chloride, where **6** was prepared by the reaction of **4** and **5** in DMF containing potassium iodide. Detail of the synthesis and characterization is elaborated in Section 2 of the Supporting Information (SI). For decoupling the direct electronic communication, the sp^3^-hybridized C9 of fluorene was adopted as a bridge linking two pentacene derivatives to afford **FlePc2**. A phenylene group was inserted between C9 and pentacene to increase the interchromophore distance to give **FlePhPc2**. **PhTIPSPc** serves as the monochromophore model for the comparison study. Their syntheses and characterization are summarized in the SI.

**Scheme 1:**
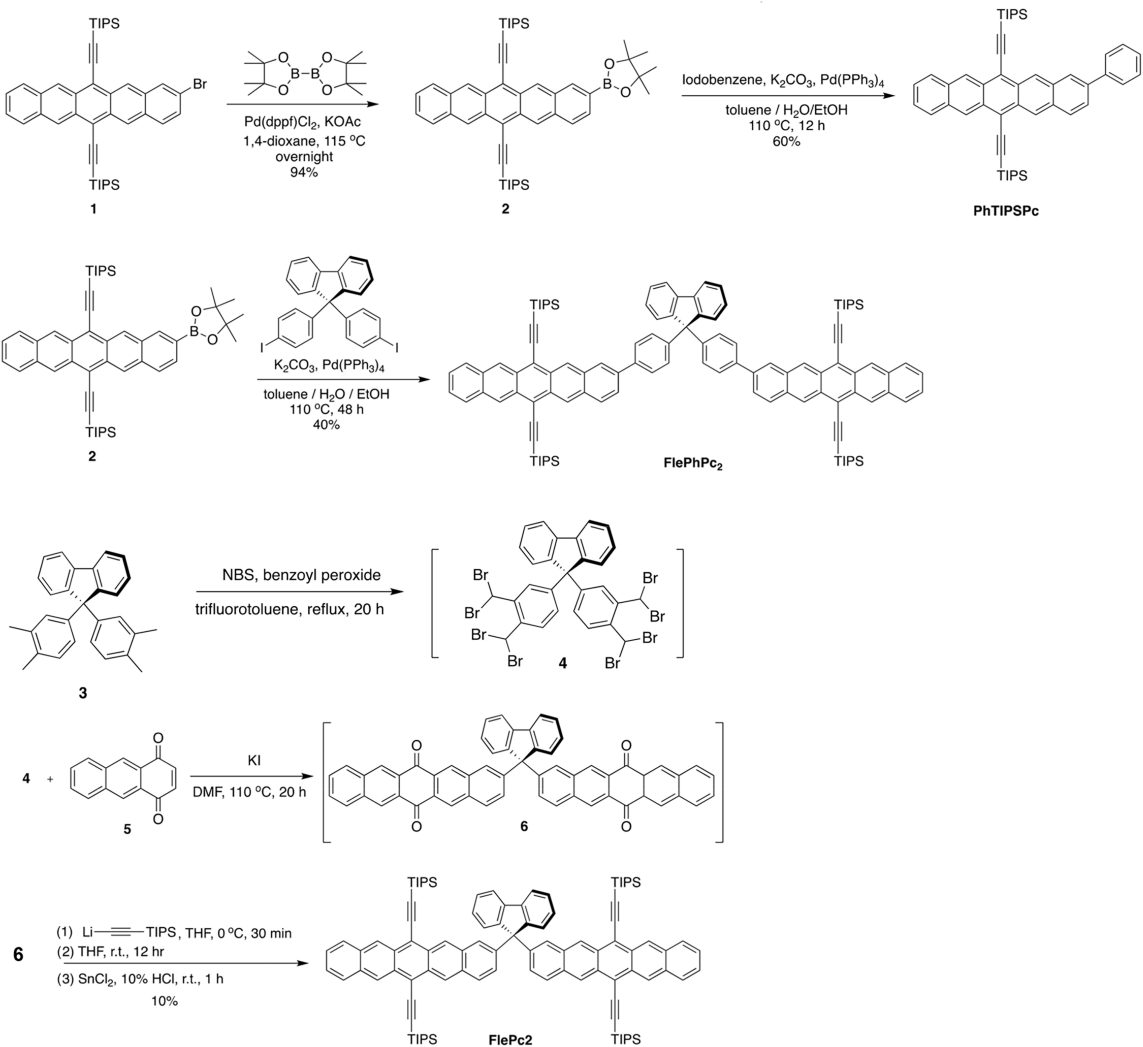
Syntheses and Structures of Molecules Studied in This Work

The crystal of **FlePc2** suitable for X-ray analysis was obtained by a two-layer solvent (chloroform/methanol) method. As shown in Figure 1, the two pentacene peripherals are close to each other with the C2–C2′ distance estimated to be 2.513 Å. The two pentacene rings are attached to the C9 of fluorene with a dihedral angle of 78°. The characteristics of this crystal structure are conducive to the process of SF.

**Figure 1:**
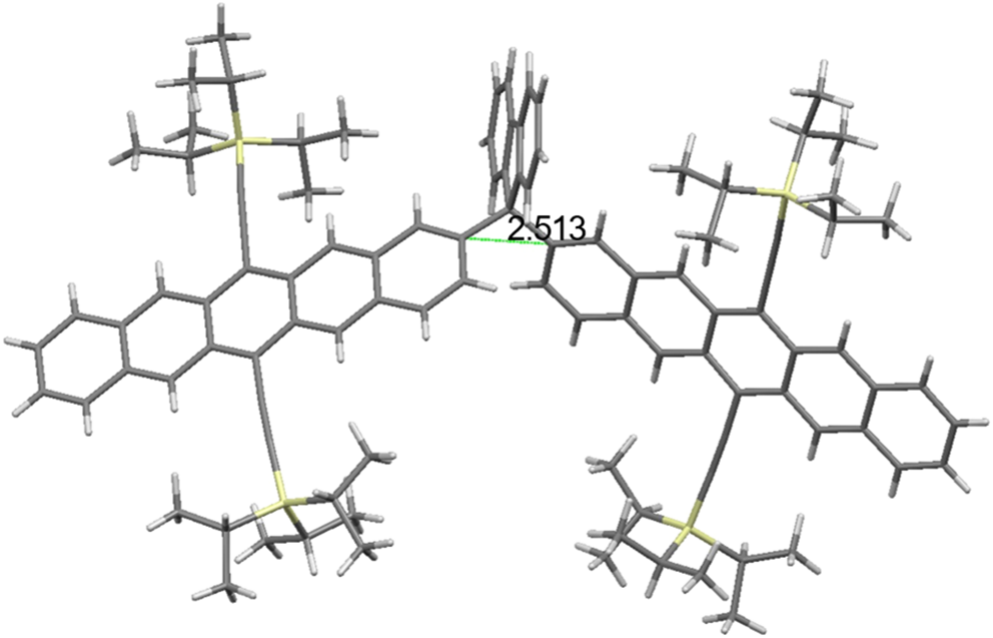
X-ray structure of **FlePc2**.

### Photophysical Properties

The steady-state absorption and emission spectra of **PhTIPSPc, FlePc2**, and **FlePhPc2** in toluene are shown in Figure 2, with additional spectra compiled in Figures S1–S3. Key parameters are summarized in Table S1. Among the reported compounds, all exhibit nearly identical absorption and emission profiles.

**Figure 2:**
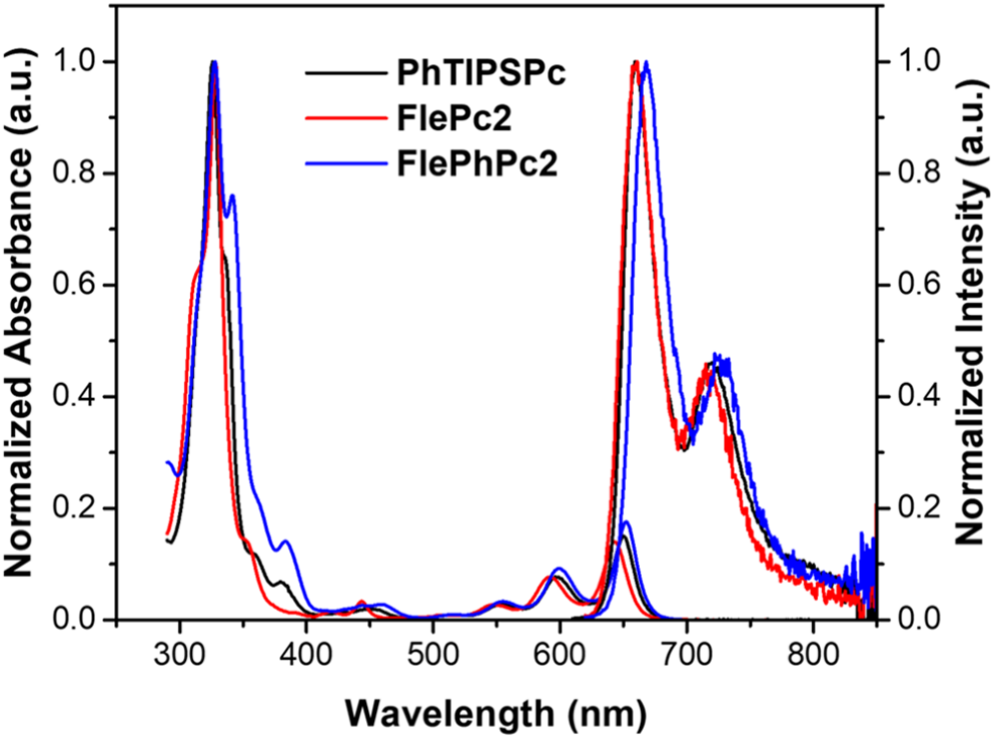
Steady-state absorption and emission spectra of reported compounds recorded in toluene.

The absorption and emission onsets of dimeric compounds closely resemble those of the monomeric reference **PhTIPSPc**. This resemblance suggests minimal contribution of the fluorene bridge to the S_0_ → S_1_S_0_ transition. Nevertheless, the bridge plays a critical role in facilitating the SF process.^35^ Specifically, it ensures the close distance between the two pentacene moieties and brings sufficient SF coupling. Notably, the strength of this SF coupling is modulated by both the chemical nature of the bridge^21^ and the relative positions of its substituents (e.g., *ortho*, *meta*, or *para*) with different extents of π-conjugation.^36,37^

The employed fluorene bridge connects both pentacene moieties to the same carbon atom of the fluorene core. Although this configuration compromises π-conjugation across the bridge, the fluorene bridge brings them into close spatial proximity and enhances through-space orbital overlap. As a result, the absorption and emission onsets of the dimeric compounds become nearly identical to those of monomeric reference **PhTIPSPc**, further reinforcing the observation that the bridge contributes negligibly to the S_0_ → S_1_S_0_ transition. This viewpoint is corroborated by natural transition orbital (NTO) analysis (Figure 3). Furthermore, the HOMO and LUMO energy levels of the fluorene are not energetically aligned with those of the adjacent pentacene units, thus hindering effective delocalization across the bridge (Table S2).

**Figure 3:**
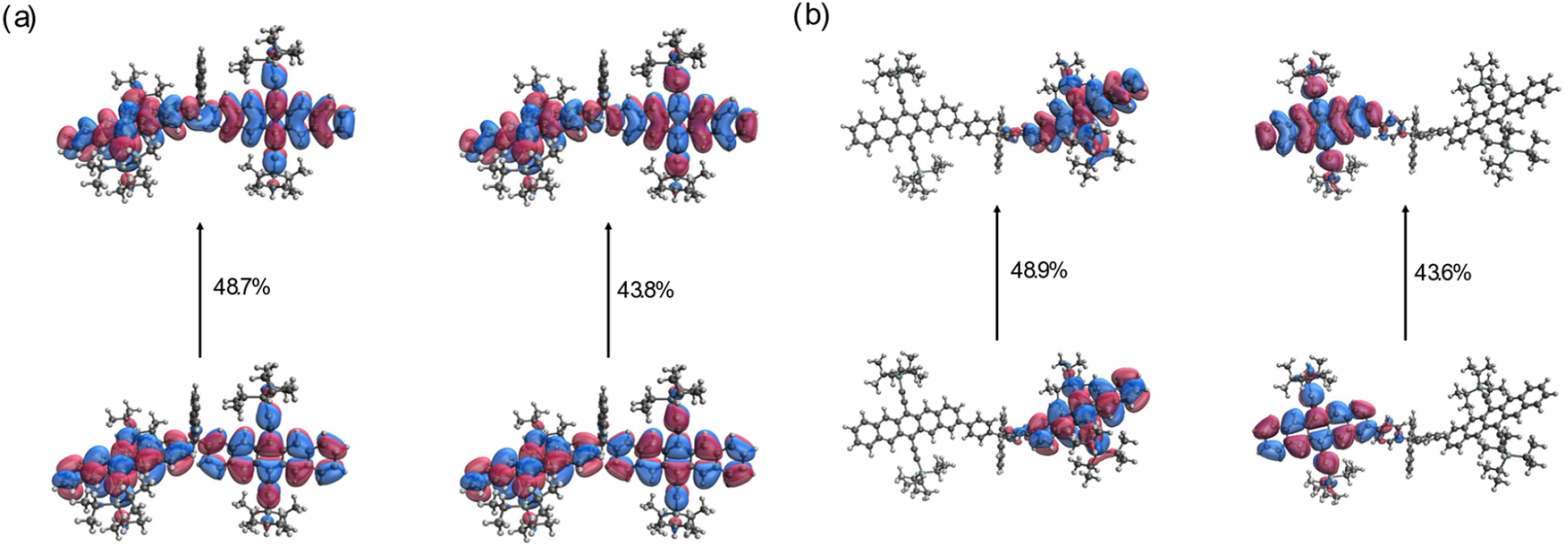
NTO analysis of S_1_S_0_ state for (a) **FlePc2** and (b) **FlePhPc2** at their S_0_ optimized structures.

Additionally, the similarity in absorption peaks indicates negligible ground-state electronic coupling between the pentacene units. This is further supported by the molar absorption coefficients (ε), which are approximately doubled for the dimers compared to the monomer, consistent with the additive absorbance of two noninteracting chromophores (Figure S3). Accordingly, ground-state electronic coupling, such as Davydov splitting or H-/J-aggregation, appears minimal. Theoretical calculations corroborate this conclusion, predicting a Davydov splitting of only ∼3 to 5 meV in their S_1_S_0_ states (Figure S4). These values are too small to be spectroscopically resolved, thus rendering the two split excitonic states effectively degenerate.^28^

Given that Davydov splitting and aggregation effects are typically observed under close intermolecular proximity, especially in the solid state, steady-state absorption measurements were also conducted under highly concentrated solutions (up to 400 μM, Figures S5–S7).^38–40^ Under these concentrated conditions, the average intermolecular distance is theoretically estimated to be approximately 8.9 nm, which may allow weak intermolecular interactions.^41^ Nevertheless, in our reported compounds, they show traceable aggregation effects across the concentrations and display linear correlation between concentration and absorbance at the monitoring wavelengths corresponding to the S_1_S_0_ state and the S_2_ state. The normalized absorption spectra remain unchanged between diluted (∼5 μM) and concentrated solutions (∼400 μM), indicating that ground-state intermolecular interactions are negligible within this concentration range (see frames c of Figures S5–S7). Therefore, a concentration was employed within the range for all subsequent fs-TA and FS-ESE measurements to ensure sufficient signal intensity while minimizing potential aggregation effects.

Based on the above results, it can be concluded that the intra- and intermolecular interactions in the ground state are minimal. We then examined the fluorescence properties to gain insight into how the molecules behave in the excited state. The fluorescence quantum yield of **FlePc2** of <0.5% in various solvents is significantly lower than those (≫7%) of the monomeric reference **PhTIPSPc** and **FlePhPc2** under the concentration of 10 μM (see Table S1), indicating the presence of a dominant nonradiative deactivation pathway for **FlePc2** in the excited-state dynamics. This phenomenon is attributed to SF, consistent with observations reported for other pentacene-based intramolecular dimers.^8,42,43^

Notably, the substantial disparity in quantum yields between the two dimers suggests that the SF rate is sensitive to structural modifications. In particular, the incorporation of phenyl substituents increases the spatial separation between the pentacene moieties. This structural change alters the SF coupling strength by approximately an order of magnitude (vide infra), thereby substantially slowing down the SF process. This interpretation is supported by fluorescence lifetime measurements shown in Figure 4 and Table 1. A fast picosecond-scale lifetime is observed for **FlePc2**, whereas no such rapid decay component is detected for **FlePhPc2**. The result indicates that, in the latter case, the SF, if any, proceeds with a time scale as slow as that (few nanoseconds and longer) of radiative decay time. Therefore, whether the SF occurs with **FlePhPc2** is thus masked through the fluorescence lifetime measurement. Also, shown in Table 1, a trace of long-lived residue with a lifetime of ∼10 ns was observed in **FlePc2**. This small but non-negligible residue cannot be removed even after thorough purification; therefore, its presence as an impurity was ruled out. It also cannot arise from residual S_1_S_0_ excitons that bypass SF, as detailed in the SI. Instead, the most plausible explanation is that the residue originates from delayed fluorescence via the bound TT triplet–triplet annihilation (TTA).^42^ The long-lived component is strongly affected by both external magnetic fields and temperature changes (Figure S8), a behavior that is consistent with a thermally activated, endothermic TTA process. The relevant results and further discussion are elaborated in the SI. Supporting Information.

**Figure 4:**
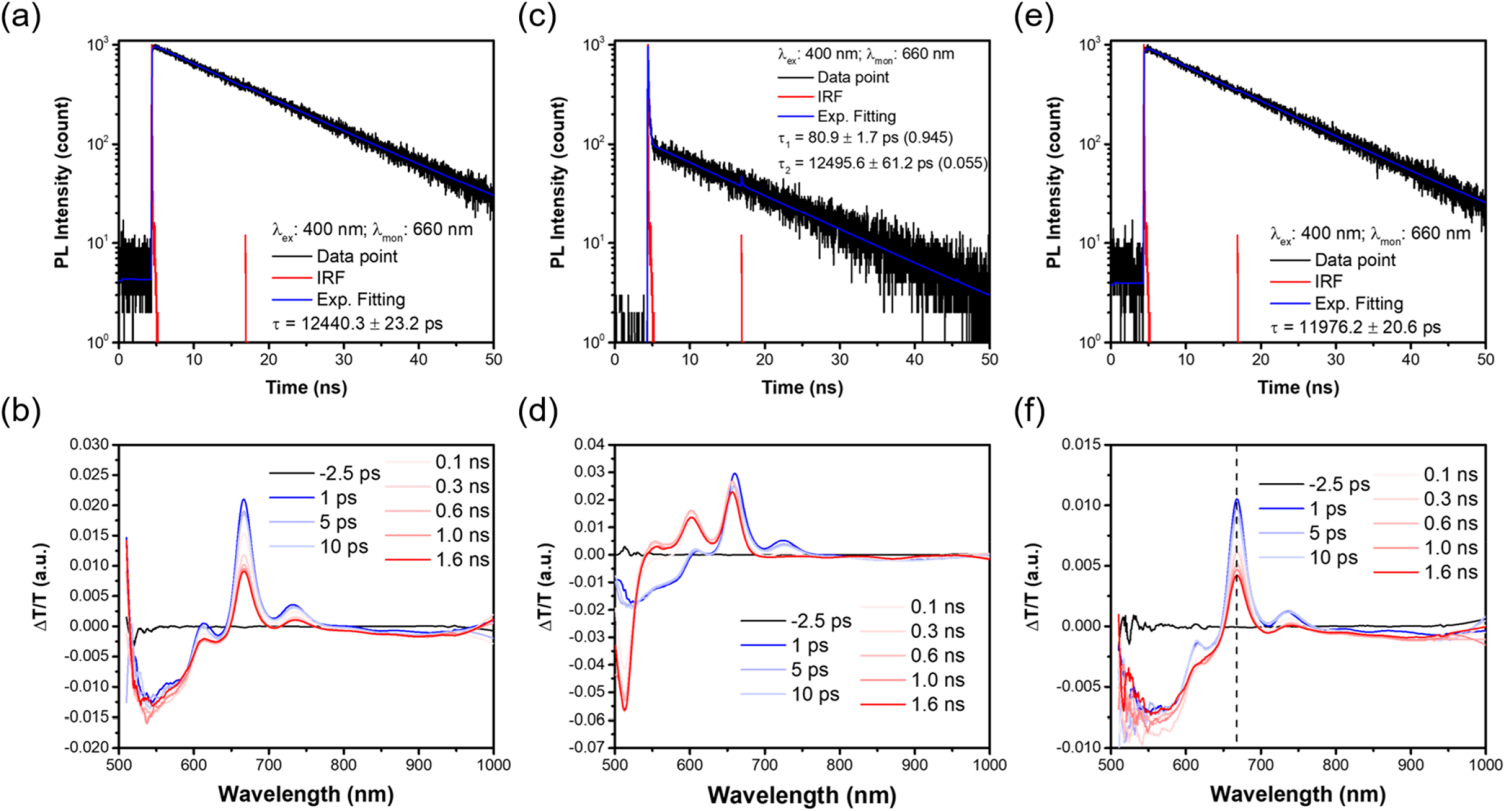
Fluorescence lifetime measurements (a, c, e) conducted at a concentration of ∼10 μM and fs-TA measurements (b, d, f) conducted at ∼100 μM in toluene for **PhTIPSPc**, **FlePc2**, and **FlePhPc2**, respectively.

**Table 1:** Photophysical Properties of the Studied Compounds in Various Solvents

molecule	solvent	λ_ex_/nm	λ_mon_/nm	τ_1_(pre-exp. factor)/τ_2_(pre-exp. factor)/ps
**PhTIPSPc**	TOL	400	660	12440.3 ± 23.2
	THF	400	660	11693.2 + 20.3
	DCM	400	660	8531.4 ± 12.3
**FlePc2**	TOL	400	660	80.9 ± 1.7 (0.945)/12495.6 ± 61.2 (0.055)
	THF	400	660	72.2 + 1.5 (0.950)/11670.0 + 59.5(0.050)
	DCM	400	660	71.7 + 1.5 (0.952)/8977.3 + 51.2 (0.048)
**FlePhPc2**	TOL	400	660	11976.2 ± 20.6
	THF	400	660	11067.2 ± 21.2
	DCM	400	660	8085.3 ± 12.5

To gain more insight into the SF process, fs-TA spectroscopy in various solvents was conducted across a broad range of delayed times, and global analysis was applied to fit the time-resolved data (Figures 4 and 5 and S9–S12, Table S3; detailed procedure is described in Section S1 of SI). In Figure 4b,d,f, alongside the ground-state bleaching (GSB) and stimulated emission (SE) (Δ*T*/*T* > 0), all reported compounds exhibited prominent excited-state absorption (ESA) features. In **FlePc2** (Figure 4d), a broad ESA band initially appears in the 500–600 nm region and is assigned to the absorption originating from the S_1_S_0_ state, as it resembles the spectral features observed in the monomeric reference **PhTIPSPc**. After ∼100 ps, the ESA profile evolves into a new pattern with a sharp peak centered at 510 nm, which is attributed to the T_1_ state absorption of pentacene.^18,43–46^ This signal gradually intensifies and reaches a steady-state level around 600 ps (Figure 5a). Furthermore, the maximum intensity at 510 nm exhibits a linear dependence on excitation power (Figure S13), consistent with a monomolecular excited-state process. Additionally, a slowly growing signal at 510 nm parallels the increase in ground-state bleaching at 600 nm, suggesting that the second pentacene moiety in the dimeric **FlePc2** is also converted to its T_1_ state through the SF process. This leads to depletion of the ground-state population and thus enhances the GSB signal as observed in Figure 5a. The SF rate constants and reaction equilibrium constants in various solvents are summarized in Table S3. Notably, **PhTIPSPc** lacks a sharp, intensified ESA at 510 nm, further indicating that the observed signal in **FlePc2** is not attributed to intermolecular interactions under the applied concentration of 100 μM.

**Figure 5:**
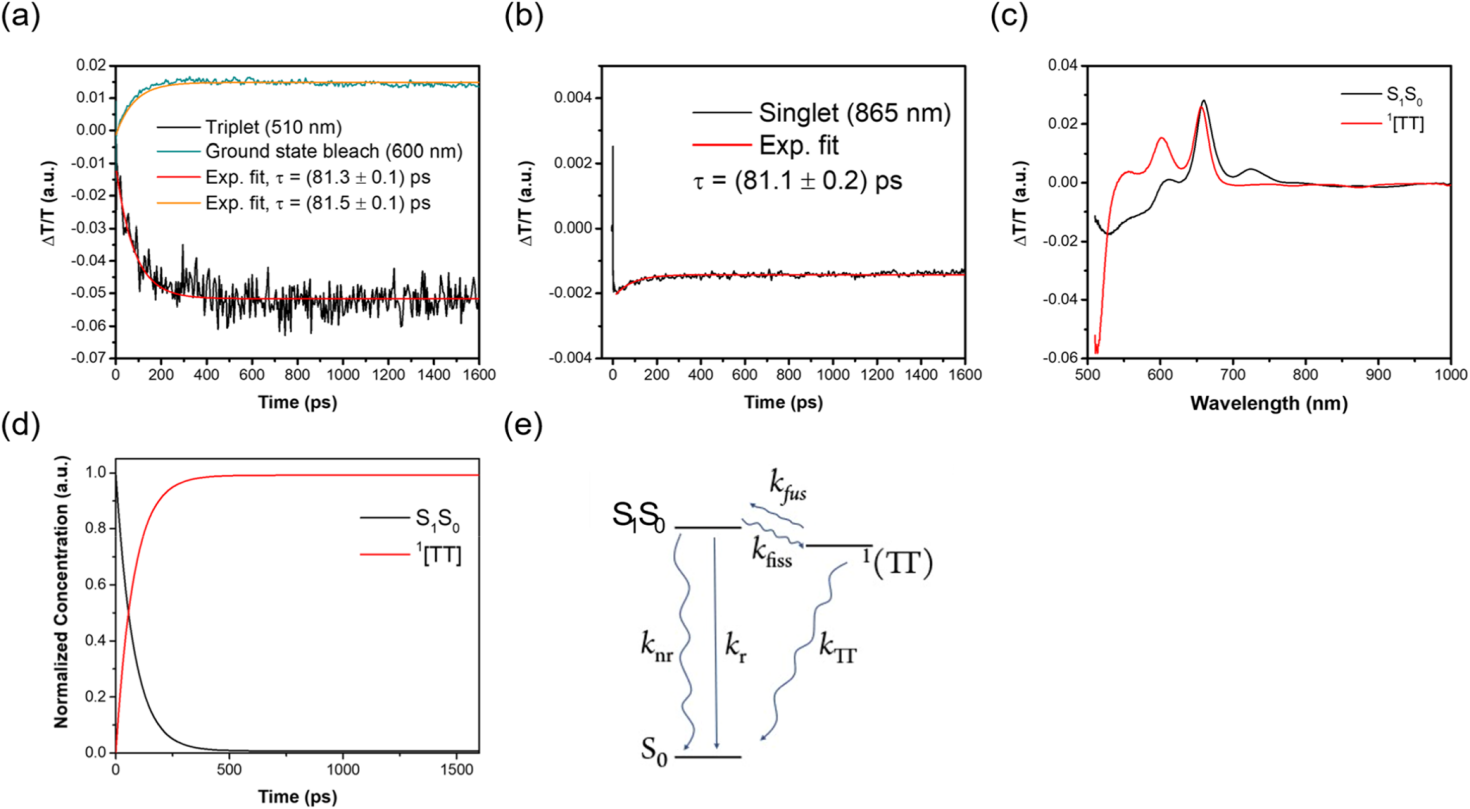
(a, b) Selected single-wavelength kinetic traces (data points) and corresponding model fits (solid lines) obtained from global analysis of fs-TA data for **FlePc2** in toluene. Notably, the identical decay time constants observed in both fs-TA and TCSPC measurements (Figure 4c) suggest that these processes correspond to precursor–successor dynamics. (c, d) show the results of the global analysis, including the species-associated spectra and the corresponding time-dependent concentration profiles, respectively. (e) illustrates the simplified kinetic model used to describe the SF process within a 1.6 ns observation window. All the measurements were conducted at ∼298 K.

Although the 510 nm ESA feature is consistent with the T_1_ state of pentacene, the subnanosecond rise time suggests it more plausibly originates from the formation of a ^1^TT via SF, occurring on a time scale faster than typical intersystem crossing (ISC). Moreover, ISC-induced triplet signals were not observed in **PhTIPSPc** within the measured 1.6 ns time window, further supporting that the ^1^TT state in **FlePc2** arises from intramolecular SF rather than ISC. In contrast, **FlePhPc2** does not display a distinct ^1^TT absorption peak within the current 1.6 ns time window, suggesting that SF either does not occur or occurs with extremely low efficiency in this compound.

To probe the lifetime monitored at 510 nm, which may originate from ^1^TT, ^3^TT, ^5^TT, or isolated T_1_ states, nanosecond transient absorption (ns-TA) spectroscopy and triplet sensitization experiments were performed, and the results are shown in Figures S14–S17. In the absence of the sensitizer PtOEP (Figures S14–S15), the ns-TA spectra of the compounds in toluene exhibit GSB, SE, and triplet absorption features consistent with the fs-TA results. When using PtOEP for triplet sensitization experiments (Figures S16–S17), the T_1_ state of pentacene was selectively populated via Dexter-type energy transfer, exhibiting a characteristic lifetime of approximately 5 μs across all reported compounds. In contrast, the lifetimes observed without the sensitizer—particularly for **FlePc2** (τ_510nm_ = 0.28 μs)—differ significantly, suggesting that the long-lived species in this case most likely arises from bound TT rather than from fully dissociated T_1_ states.

Within the nanosecond time window, it is plausible that the initially formed ^1^TT undergoes spin conversion to ^5^TT through singlet-quintet mixing,^47^ followed by further evolution to ^3^TT or dissociation into two isolated T_1_ states, as typically observed in pentacene-based SF systems. However, although the strength of spin–spin dipolar interactions between the two triplets depends on their mutual orientation and distance, the energy splitting among ^1^TT, ^3^TT, ^5^TT, and isolated T_1_ spin states does not significantly shift the absorption peak position in the visible range.^12,13^ As a result, the TA signals at 510 nm arising from these spin states are spectroscopically indistinguishable in the visible region, making it impossible to differentiate their spin states from TA alone.

To further investigate the spin dynamics, FS-ESE measurements were performed at 80 K in a toluene glass matrix under continuous-wave (CW) laser excitation at 532 nm. The rationale for conducting the experiments at 80 K is discussed in the SI, as measurements at ≤20 K are typically used in the SF community. At 80 K, **FlePc2** and **FlePhPc2** exhibit similar spectral envelopes (Figure 6a), yet the principal resonance at 3509 G is nearly twice as intense for **FlePc2**. In contrast, the monomeric reference **PhTIPSPc** shows no detectable response under identical conditions (Figure 6b), further corroborating the absence of multiexcitonic processes in a single pentacene. The observed EPR signals are unlikely to originate from SF-induced pentacene radical ions, as neither fs-TA (Figure S18)^48,49^ nor FS-ESE measurements^50^ detected such species. Consequently, the intensity trend, **FlePc2** > **FlePhPc2** ≫ **PhTIPSPc** ≈ 0, links the magnitude of the EPR signal to their intramolecular geometry and the probability of generating high-spin ^5^TT state via intramolecular SF. Since the SF rate of **FlePhPc2** is calculated to be on the same order as the pentacene’s radiative rate from its S_1_S_0_ (vide infra), the population reaching ^5^TT therefore dwindles, resulting in a diminished ^5^TT signal in the FS-ESE result.

**Figure 6:**
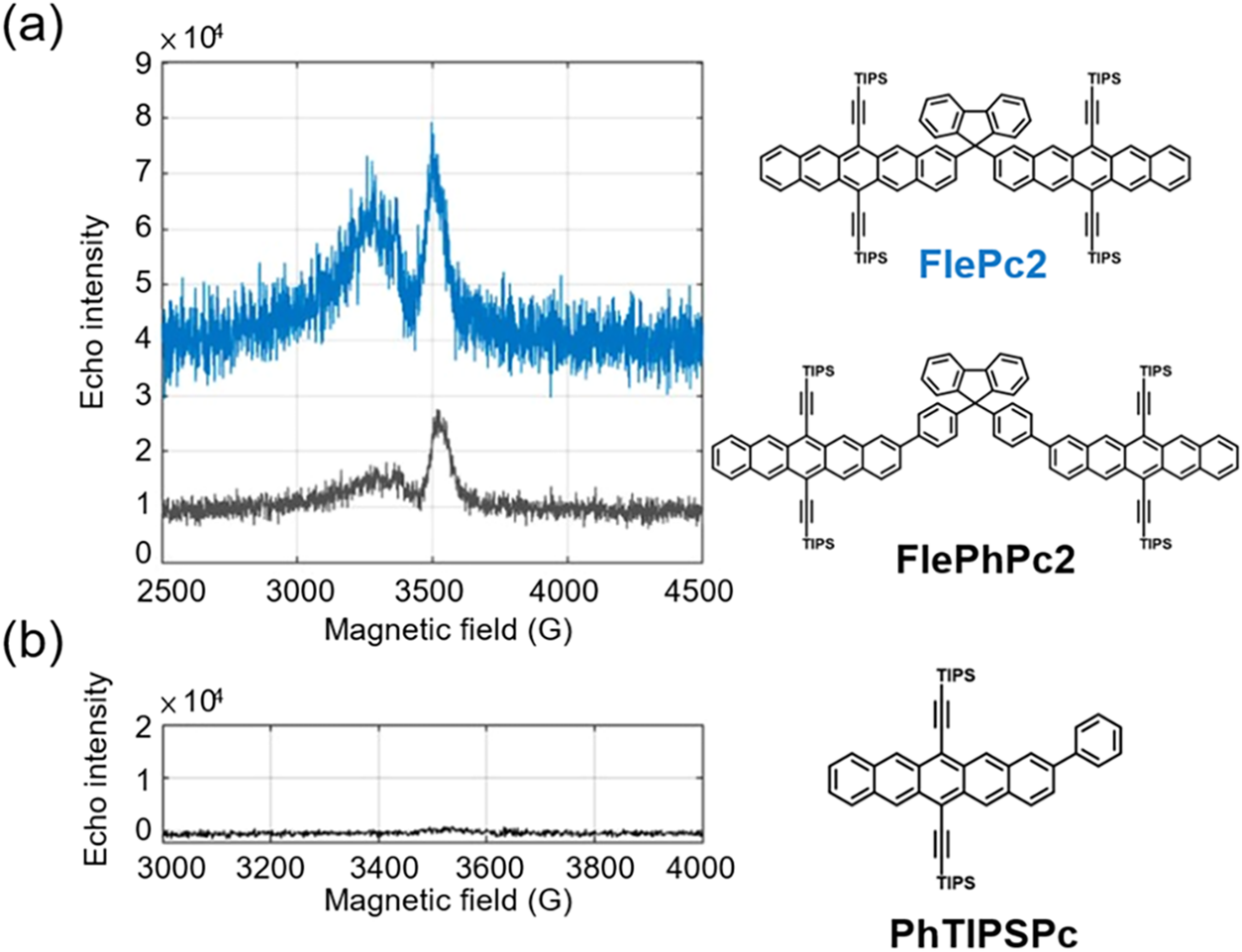
FS-ESE spectra recorded for (a) **FlePc2** and **FlePhPc2**, and (b) **PhTIPSPc** under CW laser excitation at 532 nm. All samples were measured in flash-frozen toluene at 80 K. The concentrations were 0.3 mM for the dimeric species **FlePc2** and **FlePhPc2**, and 0.6 mM for the monomeric species **PhTIPSPc**. Measurements for **FlePc2** and **FlePhPc2** were performed using identical scan parameters to allow direct comparison of signal intensities. In (a), the slightly higher baseline noise in the **FlePc2** trace arises from the longer integration window used to capture its broader spin–echo signal, not from differences in receiver gain or detection sensitivity.

Remarkably, the EPR signals of both dimers remain stable within ±3% over 15 min after switching off the CW laser excitation, showing no appreciable decay throughout the measurement period at 80 K. A noticeable decrease in signal is only observed above ∼130 K (Figure S19), suggesting that the spin states formed at low temperatures are highly persistent. This thermal stability is likely related to exciton trapping below the glass transition temperature (*T*_g_) of the solvent. Although the *T*_g_ of pure toluene is ∼117 K,^51^ the presence of solutes in the system alters the effective *T*_g_, thus influencing the mobility and decay pathways of the spin states.^52^ These results suggest that depopulation of the ^5^TT state occurs primarily via intermolecular quenching processes, which are suppressed in the frozen matrix due to restricted molecular diffusion.

The FS-ESE spectrum of **FlePc2** was simulated with the EasySpin toolbox using a model of two exchange-coupled spin1 centers, the minimal description for the TT state. The best agreement (Figure 7a) is obtained with a spin–spin exchange interaction (*J*) of 25 GHz and triplet zero-field splitting parameters (*D* = 1650 MHz, *E* = 150 MHz). The positive sign of *J* indicates antiferromagnetic coupling between the two triplets, placing the ^5^TT state energetically above the ^1^TT state (vide infra). Moreover, the magnitude of *J* (|*J*| > |*D*|) signifies a strong exchange-coupling regime, forming well-defined ^5^TT state with *S* = 2.^13,26^ Notably, the *D* and *E* values for the triplet state of pentacene in our reported dimers are relatively large compared to those reported for other pentacene dimers undergoing SF.^13,20,53–55^ However, these parameters are known to be sensitive to variations in mutual chromophores’ orientation.^56^

**Figure 7:**
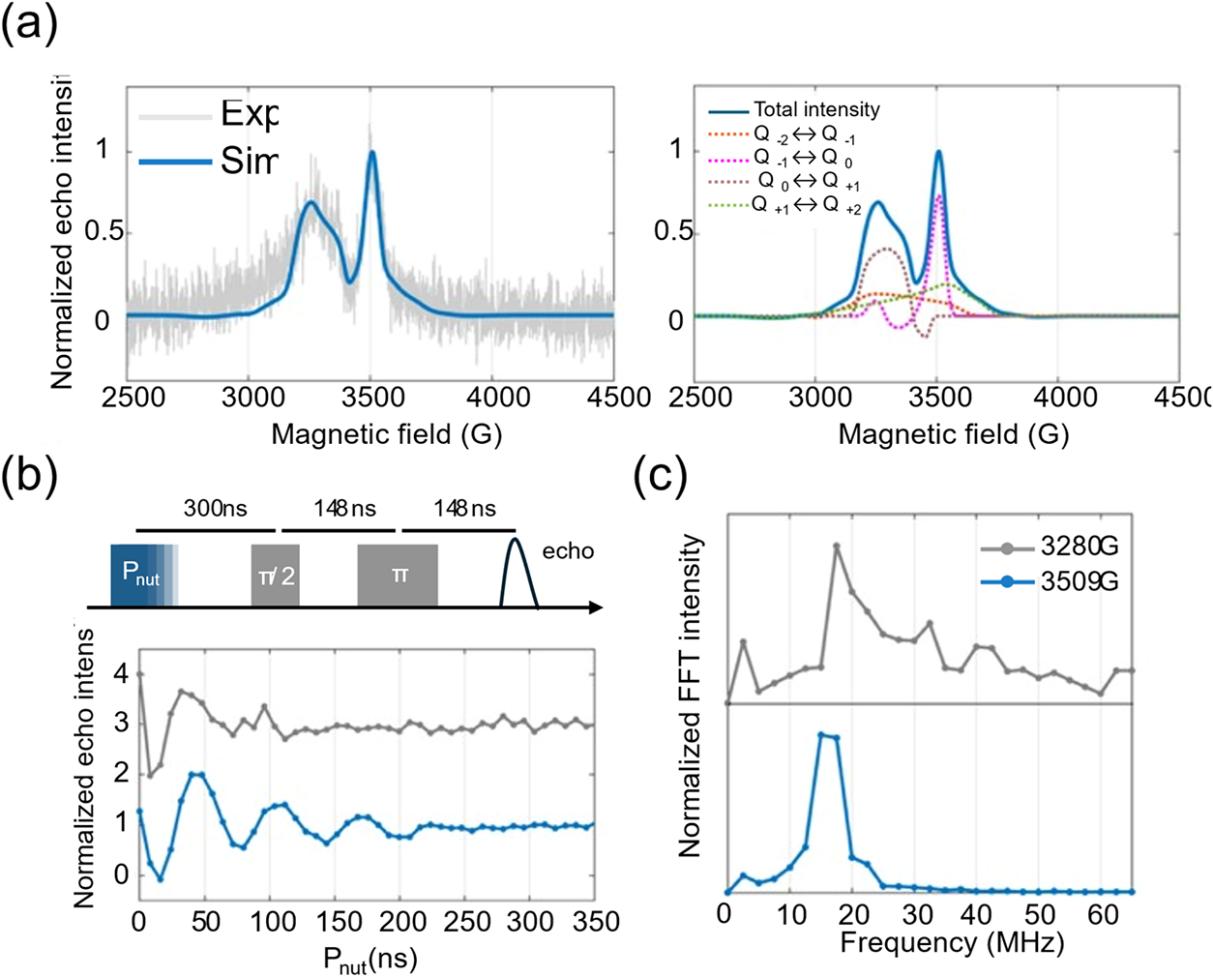
(a) Left panel: ^5^TT simulation of the FS-ESE spectrum for **FlePc2** using optimized spin Hamiltonian parameters. Right panel: Simulated individual transitions within the quintet spin manifold, illustrating detailed spin polarization characteristics. (b) Upper panel: Pulse sequence utilized in the nutation experiments. Lower panel: Experimental Rabi oscillations measured at magnetic fields of 3280 and 3509 G for **FlePc2**, recorded in flash-frozen toluene at 80 K under 532 nm CW laser excitation. (c) Frequency-domain spectra derived from the Fourier transform analysis of the recorded Rabi oscillations confirm identical nutation frequencies at both field positions.

To account for inhomogeneous broadening effects arising from structural heterogeneity and molecular interactions, an additional line width broadening parameter of 300 MHz was included in the simulation. This adjustment effectively reproduced the experimental FS-ESE spectral profile. These parameters yield the optimized quintet sublevel populations, [0.13, 0.11, 0.16, 0.31, 0.29] for Q_+2_ to Q_–2_, successfully reproducing the observed net emissive/absorptive pattern and confirming the presence of non-Boltzmann spin polarization generated during SF. Further details of the non-Boltzmann sublevel distribution are provided in SI. Pulsed EPR nutation traces were recorded at 3280 G and 3509 G, fields corresponding to the two dominant maxima in the FS-ESE spectrum (Figure 7b). According to the simulation, these fields monitor the *m*_s_ = 0 → +1 and *m*_s_ = –1 → 0 transitions, respectively, within the *S* = 2 manifold. For a quintet, the nutation frequencies for these transitions are identical, as described by eq 1.

1 $$\omega \left(m_{s},m_{s}\pm 1\right)\propto \sqrt{\left[s\left(s+1\right)-m_{s}\left(m_{s}\pm 1\right)\right]}$$

Fourier transformation indeed yields overlapping peaks at 17.5 MHz for both field positions (Figure 7c), conclusively assigning the entire spectrum to a single quintet species. A shoulder at 14.5 MHz is also observed when monitoring at 3509 G, which may arise from the *m*_s_ = ±2 → ±1 transitions, as its frequency is ∼0.81 times that of the *m*_s_ = ±1 → 0 transitions. This observation further excludes the presence of lower-spin triplet states such as ^3^TT or isolated T_1_ states, which typically yield a nutation frequency of 10.1 MHz, calculated by eq 1, after Fourier transformation.

Considering the 100 ns dead time inherent to pulsed-EPR detection, continuous-wave transient electron paramagnetic resonance (trEPR) measurements were additionally performed to assess whether any precursor species might exist prior to the formation of the ^5^TT state (Figure S19). The trEPR results confirm that the long-lived ^5^TT remains the dominant spin species in **FlePc2**, and no additional transient signals were detected. These results verify that no short-lived radical or triplet intermediates were missed in the pulsed-EPR measurements, and they are fully consistent with the FS-ESE observations.

The trEPR results, together with theoretical simulations, confirm that the long-lived ^5^TT state is the dominant spin-active species in **FlePc2** within the coherence-relevant regime. To further corroborate the dominance of the quintet state, it is essential to enhance sensitivity to any potential triplet contributions. Since triplet states exhibit lower Rabi frequencies than the high-spin quintet state, increasing either the delay time after the laser flash or the pulse width can, in principle, improve sensitivity to triplet-derived signals.

Accordingly, we selected two representative time regimes corresponding to the formation and subsequent quenching of the quintet state, enabling a direct comparison of potential triplet contributions between the two regimes. The corresponding results are shown in Figure S20. No additional transient signals attributable to triplet species were detected under any of these conditions.

These results indicate that any triplet population generated in the trEPR experiment either (i) remains at a level too small to produce a detectable spin echo under our experimental conditions, or (ii) relaxes or dephases too rapidly to yield an observable echo signal. By contrast, the quintet state retains sufficient coherence for robust echo detection, indicating that the spin–echo-active photoproduct is dominated by the quintet state, with relaxation into the ^3^TT or T_1_ manifold being negligible within the echo-detectable regime.

Furthermore, the *T*_m_ of **FlePc2** at 80K, measured at both 3280 and 3509 G, exhibited identical decay behaviors (*T*_m_ = 481 ± 20 ns). The results further corroborate that these transitions originate from the same quintet state, differing only in their magnetic spin quantum numbers (Figure S21). These findings highlight the potential of the present compounds as promising qubit candidates, as the involvement of additional magnetically responsive states, such as ^3^TT or isolated T_1_ excitons, would inevitably induce state mixing and field-dependent spin precession, thereby accelerating dephasing and shortening the decoherence time.^31^ In contrast, the quintet states observed in the FS-ESE spectra of the reported compounds persist without spin conversion to ^3^TT or dissociation, effectively suppressing such pathways and supporting prolonged coherence.

### Theoretical Calculation

To simulate the SF process and provide evidence for ^5^TT stabilization, we employed DFT, time-dependent DFT (TDDFT), and the RAS-2SF approach. Further computational details are provided in the SI, Section S1.

For the dimeric compounds, the S_1_S_0_ configuration forms after optical excitation and subsequent relaxation within several picoseconds, and its structure is corroborated by theoretical results shown in Table S4. This indicates that the two pentacene moieties in the dimers behave as two independent chromophores in their relaxed excited-state structure. As shown in Figure 2, the emission spectra of the dimers are nearly identical to that of the monomeric reference **PhTIPSPc**, suggesting that the mutual interaction between the two pentacene moieties in the relaxed S_1_S_0_ configuration is negligible.

Subsequently, the ^1^TT state forms via the SF process. Although the SF rate can be simulated with RAS-2SF method, this yields only relative values rather than absolute rates, since RAS-2SF does not directly provide the absolute value of the electronic coupling in eV or the correct Franck–Condon weighted density of states (FCWD).^57^ In addition, because RAS-2SF does not explicitly include dynamic electron correlation, the simulation generally overestimates excitation energies.^10^ Nevertheless, it can qualitatively describe adiabatic wave functions and interstate couplings via one-particle transition-density matrices (∥*γ*∥^2^).^58,59^

To address these limitations, we applied the *θ*-optimized fragment excitation difference (FED) scheme within the RAS-2SF framework to estimate the SF and TTA coupling strengths, which were then used along with Fermi’s golden rule to calculate the FCWD and the associated SF rates.^35,60^ The results for the convergence test, electronic couplings, FCWD, and SF rates are summarized in Tables 2, S5 and S6, and the FCWD functions are depicted in Figure S22. The SF coupling strengths between the S_1_^D^S_0_^A^ configuration and the ^1^TT state are referred to as *V*_FED_. Among the dimers, **FlePc2** exhibits the strongest SF coupling, while **FlePhPc2** shows an order-of-magnitude weaker coupling. Furthermore, the ratio of the SF to TTA rate constants (denoted as the equilibrium constant, *K*_eq_) agrees well with results obtained from global fitting of transient absorption data (Table S3).

**Table 2:** Theoretical Calculation Data of the Rate for the Reported Compounds at Their S_1_S_0_ Geometry

	singlet fission			triplet–triplet annihilation			
	Calc.		Exp.	Calc.	Exp.^a^	K_eq_ ^b^	
molecule	|*V*_FED_|/meV	*k*_*S*F_(×10^8^)/s^–1^	*k*_*S*F_(×10^8^)/s^–1^	*k*_TTA_(×10^6^)/s^–1^	*k*_T_(×10^6^)/s^–1^	Calc.	Exp.
**FlePc2**	0.57	53	123	30.2	0.156	175.5	136.0
**FlePhPc2**	0.15	3.8		2.17	0.182		

a The values are derived from the decay of the T_1_ state, as obtained through the sensitization experiment.

b The values are calculated by *k*_SF_/*k*_TTA_.

For the excitation energy of ^1^TT, its energy level was calculated using the broken-symmetry DFT (BS-DFT) method.^61^ Through singlet–quintet mixing, ^5^TT was formed and characterized with unrestricted DFT (UDFT). In addition, the formation of ^5^TT is facilitated by structural fluctuations, which modulate *J* and promote a multiexcitonic quintet configuration.^14,55^ This observation is consistent with the dimer fluctuations identified in our theoretical analysis (Figure S23). Gas-phase calculations suggest that these molecular fluctuations raise the energy of ^5^TT state above that of ^1^TT state, which further hampers dissociation into isolated T_1_.^62^

To quantify this dissociation barrier, the binding energy (*E*_b_), estimated as *E*(^5^TT) – *E*(^1^TT), was found to be 81 meV for **FlePc2** and 86 meV for **FlePhPc2**. While these computed values are probably overestimated, they are primarily indicative of qualitative SF behavior. These positive *E*_b_ values reflect the magnitude of *J*, a key factor in ^5^TT stabilization. To gain further insight, we examined the spin density distribution of the quintet state for both dimeric compounds.^32,63,64^ Moreover, the small spin density at the C9 position of the fluorene bridge in the ^5^TT state (Figures 8 and S24) suggests possible interactions between the σ-orbitals of the bridge and the π-orbitals of pentacene in the quintet-state conformation, enabling σ–π overlap.

**Figure 8:**
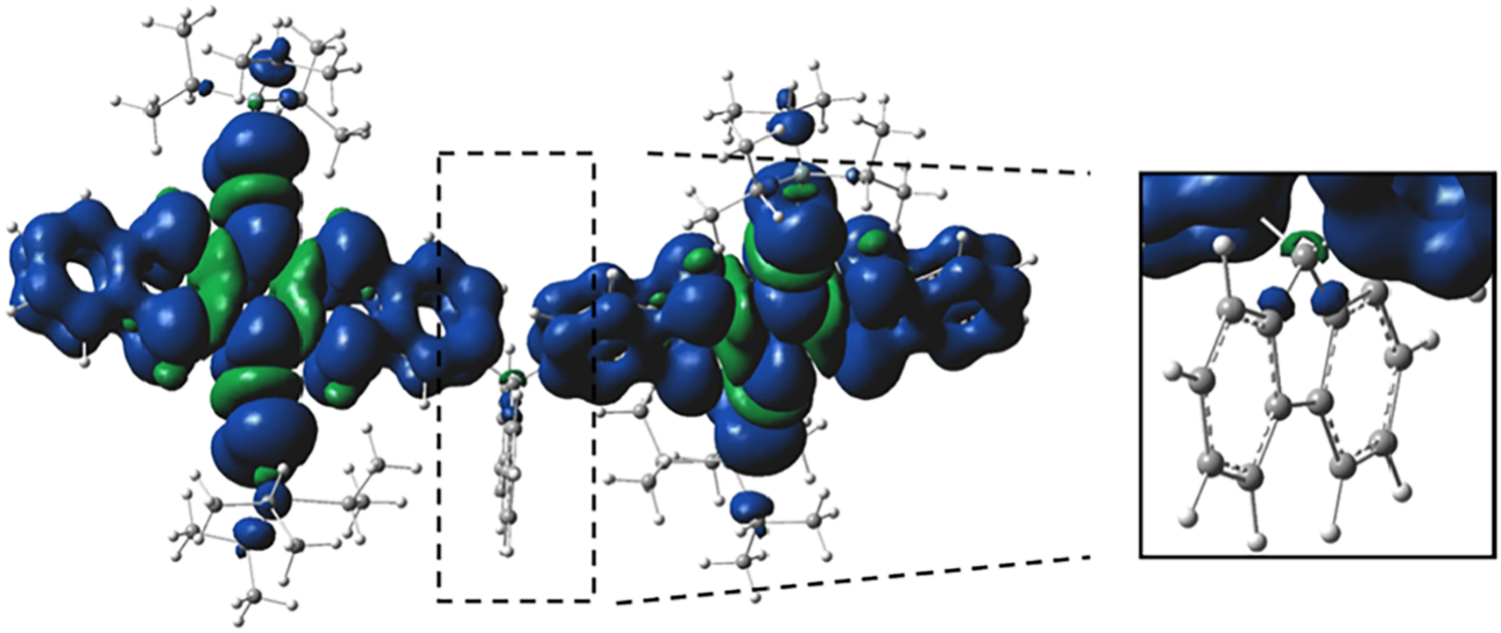
Spin density distribution of the **FlePc2** of ^5^TT and its enlarged view, obtained by *ωB*97x-D/6–31G­(d,p). Isovalue for spin density is 0.0004.

This leads to substantial calculated *J* for **FlePc2**. Furthermore, when employing the quintet-state conformation of **FlePc2** in the RAS-2SF/FED single point calculations, the coupling between the S_1_S_0_ and ^1^TT states was found to be 42 meV. This large coupling value could further facilitate the TTA process, which may explain why the lifetime of the 510 nm signal observed in the ns-TA data decays more rapidly than that of an isolated T_1_ (Figures S15 and S17). Collectively, these findings provide possible evidence for the stabilization of the quintet state.

## Conclusion

In summary, we designed and synthesized pentacene-based dimers connected via a fluorene bridge at a single carbon position to investigate their SF behavior and spin dynamics. This unique single-point attachment enforces close spatial proximity between the two chromophores, enabling strong through-space electronic coupling while minimizing π-conjugation across the bridge.

Among the studied compounds, **FlePc2** demonstrated efficient intramolecular SF, as confirmed by RAS-2SF calculations combined with FED analysis, which align closely with the kinetic parameters obtained from transient absorption and lifetime measurements. FS-ESE and trEPR unambiguously revealed that the spin–echo-active photoproduct is dominated by the ^5^TT, with relaxation into the ^3^TT or T_1_ manifold being negligible within the echo-detectable regime. The consistency further substantiates the predominant formation, stabilization, and persistence of the ^5^TT state. Our work demonstrates that simple molecular modifications—specifically, the symmetrical single-point attachment of two pentacenes to a fluorene bridge—can profoundly reconfigure SF dynamics.

This fluorene-single-carbon bridged architecture stabilizes the ^5^TT state within the coherence-relevant regime while suppressing dissociation and spin conversion pathways. Importantly, by regulating the spin density distribution across the two pentacene units, this design provides a versatile molecular platform for achieving temperature-robust, quintet-selective spin coherence and systematically probing the relationship between spin density, quintet stability, and spin coherence. Such features highlight a complementary design strategy to highly rigid scaffolds, opening new opportunities for molecular spintronics and quantum information applications.

## Supplementary Material





## Notes

Reagents and materials, synthesis and characterization of the target compounds, detailed measurements, computational details, crystal, spectroscopic data, and other supporting data (PDF)
Structure Factor tables (PDF)
