# Supplementary material for: Single-Carbon Bridged Pentacene Dimers Enable Efficient Singlet Fission and Quintet State Stabilization
Source: J Am Chem Soc. 2026 Jan 23;148(4):4062–73. doi: 10.1021/jacs.5c14851 (PMC12879938; doi:10.1021/jacs.5c14851)
Supplement: Supplementary file 1 [file ja5c14851_si_001.pdf]

Supporting information for:

Single-Carbon Bridged Pentacene Dimers Enable Efficient Singlet Fission and Quintet State Stabilization

Chao-Hsien Hsu [a], Yi-Ching Liao [a], Chu-Chun Cheng [b], Bo-Han Wu [b], Chou-Hsun Yang [c], Chao-Ping Hsu [c, d], Bo-Han Chen [e], Shang-Da Yang [e], Yuling Hsu [a], Li-Kang Chu [b, *], Yun-Wei Chiang [b, *], Ken-Tsung Wong [a, f, *], Pi-Tai Chou [a, *]

[a] Department of Chemistry, National Taiwan University, No. 1, Sec. 4, Roosevelt Rd., Taipei 106319, Taiwan

[b] Department of Chemistry, National Tsing Hua University, Hsinchu 300044, Taiwan

[c] Institute of Chemistry, Academia Sinica, 128 Section 2 Academia Road, Nankang, Taipei 11529, Taiwan

[d] Physics Division, National Center for Theoretical Sciences, Taipei 10617, Taiwan

[e] Institute of Photonics Technologies, National Tsing Hua University, Hsinchu 300044, Taiwan

[f] Institute of Atomic and Molecular Sciences, Academia Sinica, Taipei 10617, Taiwan

Corresponding authors: Email: lkchu@mx.nthu.edu.tw (L.-K. Chu); ywchiang@mx.nthu.edu.tw (Y.-W. Chiang); kenwong@ntu.edu.tw (K.-T. Wong); chop@ntu.edu.tw (P.-T. Chou)

Table of Contents

Section 1. Materials and General Methods 2

Section 2. Synthesis 7

Section 3. Photophysical Measurements 12

Section 4. Quantum Chemical Calculations 33

Section 5. ^{1}H and ^{13}C NMR spectra 115

Section 6. X-ray Crystallography 119

Section 7. Reference 121

Section 1. Materials and General Methods

X-ray Crystallography

Crystallographic data were collected at 100(2) K on a Bruker Venture diffractometer using IμS Diamond II Cu-Kα radiation (λ = 1.54178Å). Cell parameters and data reduction were retrieved and refined by manipulating Apex6 software on all reflections. The structures were solved and refined with SHELXL programs. The hydrogen atoms were included in calculated positions and refined using a riding mode.

Photophysical Measurements

In this study, unless otherwise specified, were performed at RT (298 K) and conducted using a 10-mm square cell. UV-visible NIR spectrophotometer HITACHI UH5700. The steady-state emission spectra and excitation spectra were measured with a spectrofluorometer (Edinburgh FL 980), and lifetimes were also determined by its time-correlated single-photon counting (TCSPC) system with EPL-634 nm diode lasers as the excitation source. Both the wavelength-dependent excitation and emission responses were calibrated. In this study, we used LDS 821 as a reference dye, for which the photoluminescence quantum yield (PLQY) is 12.1% in dichloromethane (DCM).[1]

Femtosecond Transient Absorption (fs-TA)

The schematic setup of our light source is illustrated in the previous work.[2] Briefly speaking, the measurements were performed using a commercial Yb: KGW laser system (Pharos, Light Conversion) with a central wavelength of 1030 nm, an average power of 2.5 W, a repetition rate of 3.125 kHz, a pulse energy of 800 μJ, and a pulse duration of 190 fs. Two identical pulses were produced with a low-GDD 50/50 beam splitter and passed through our designed nonlinear compressor, named multiple plate compression (MPC). For this experiment, a high-pass filter with a cut-off wavelength of 980 nm was applied. Pulse compression was achieved with 8 chirped mirror bounces (Ultrafast Innovation), thus removing the material dispersion introduced by the optics before the sample. The compressed pulses had an FWHM duration of 3.2 fs and were characterized at the sample position with a polarization-gating frequency-resolved optical gating (PG-FROG). All samples were loaded into a 1 mm cuvette for the fs-TA measurements.

Step-Scan Pump-Probe Time-Resolved UV–Vis Measurements (ns-TA)

Step-scan UV–vis spectroscopy was performed using a Vertex 80 spectrometer (Bruker) with a Si diode detector, and a second harmonic of a Q-switched Nd: YAG pumped Ti: Sapphire laser (Tsunami, Spectra Physics) at 380 nm was used as the pump pulse, which has a pulse width 8 ns and a repetition rate of 10 Hz. All step-scan ns-TA measurements were performed in a 2 mm cuvette, and all samples were degassed using a freeze–pump–thaw cycle at least three times to remove dissolved oxygen. This ensured that the T_1 state of pentacene—

generated via the singlet fission process—would not be quenched by oxygen and that its signal could be reliably detected. The background reduction and optimization of pumping power required various combinations of interference filters, and this procedure was necessary to avoid thermal noise and sample decomposition. Typical excitation energy was adjusted to lower than 200 mJ cm^{-2}. An optimized laser pulse had a signal-to-noise ratio adjusted to avoid damaging the sample. Moreover, the excitation source was synchronized with the spectrometer and was set to 45° with respect to the UV-vis probe beam to maximize the pump-probe overlap. In addition, the visible source (tungsten lamp, 24 V, 150 W) was employed as an external source coupled with a water-cooling unit and power supply.

In the triplet sensitization experiment, Pt(II) Octaethylporphyrin (PtOEP) was selected as the sensitizer for the target compound. The concentrations of PtOEP and the target compounds were set to 5×10^{-4} M and 1×10^{-4} M, respectively. Under 380 nm optical excitation, the target compounds were insufficiently excited because their absorbance was approximately 35-fold lower than that of PtOEP. The triplet lifetime of the sensitizer PtOEP was fitted to 28.8 μs (Figure S16 and S17), which was consistent with the time-correlated single photon counting (TCSPC) results. Upon mixing with the target compounds, the excited triplet state absorption of PtOEP was partially quenched, and the signal of the target compounds gradually increased. Notably, upon mixing the sensitizer and the target compounds, the entire wavelength range in the transient absorption profile displayed a different pattern from that of the sensitizer, indicating that the energy transfer process had occurred.

Since the Si diode detector used in the step-scan technique probes signals in the visible range, achieving simultaneously high spatial and temporal resolution is challenging. Obtaining both high spatial and temporal resolution would require an MCT detector coupled with a cooling system. However, the MCT detector cannot operate in the visible wavelength range, and its scanning range, expressed in wavenumbers, is considerably narrower. The detection range reaches only a few thousand cm^{-1}, compared to the broader range of absorption in the tens of thousands of wavenumbers. In the step-scan FTIR experiment, maximizing temporal resolution, therefore, unavoidably required a sacrifice of spectral resolution. Nevertheless, monitoring the decay lifetime at a single wavelength still provides valuable reference information. Therefore, to achieve high temporal resolution,

we were limited to choosing only 51 specific points ranging from 431 nm to 920 nm, while the overall spectral shape is still discernible. We then selected specific time points to create the corresponding 2D spectra, as shown in Figure S13 and S15.

Global Analysis by Multivariate Curve Resolution (MCR) Method

The fs-TA data were processed by multivariate spectral analysis, specifically multivariate curve resolution (MCR) analysis.[3] This approach allowed us to decompose the raw TA data into the pure state spectra based on the kinetic model present in Figure 5(e). When performing MCR, the number of components was set to be two for fs-TA measurements of all the target compounds. A non-negativity constraint and a closure constraint were also applied in the concentration profile. The optimization process fulfills a single criterion until the termination, known as the lack of fit (LOF). The optimization is usually finished when the difference in model fit between consecutive iterations does not improve significantly (e.g., a difference of less than 0.1% among the lack of fit between consecutive iterations).

Field-Swept Electron Spin Echo (FS-ESE) Measurements

EPR measurements were conducted using a Bruker ELEXSYS E580-400 X-band CW/pulsed spectrometer, equipped with an EN4118X-MS3 split-ring resonator and a cryogenic ultra-low-noise microwave amplifier to ensure optimal sensitivity and resolution.[4, 5] Pulsed EPR experiments employed the Hahn echo detection sequence. Microwave pulse powers were carefully adjusted to maximize the echo signal intensity from each sample. Optimal pulse lengths of 16 ns for the $\pi/2$ pulse and 32 ns for the π pulse were selected. Spin nutation experiments were performed by replacing the initial $\pi/2$ pulse with a variable-duration pulse ranging from 0 to 400 ns, allowing precise determination of the nutation frequencies and characterization of the spin states involved. Measurements were carried out at cryogenic temperatures in flash-frozen toluene solutions to stabilize paramagnetic species. The combination of light irradiation and EPR detection followed the previous instrumentation.[6] The samples were excited through the top of the sample holder with a 532-nm CW laser module (DPGL-2100, SUWTECH LASER) via a 7-meter-long optical fiber with a 1.0 mm diameter (QMMF-UVVIS-1000, OZ Optics, Ottawa, Ontario, Canada). The laser energy at the exit of the optical fiber was roughly 2.0 mW.

We employed FS-ESE rather than conventional continuous-wave transient electron paramagnetic resonance (trEPR) because, at ≤130 K (below glass transition temperature of the matrix), the photoinduced spins are long-lived and phase-coherent, so echo detection refocuses inhomogeneous broadening and, via time gating, suppresses laser/resonator artifacts to yield non derivative, flat baseline spectra while avoiding CW saturation and power broadening. These conditions enable quantitative simulations of the quintet (^{5}TT), allowing extraction of zero-field splitting parameters, intertriplet exchange, and sublevel populations with improved confidence. The

pulsed framework further supports nutation and echo decay at selected fields to verify spin multiplicity and determine phase-memory time T_m.

Continuous-wave transient electron paramagnetic resonance (trEPR)

Unless otherwise specified, trEPR data were collected at X-band (9.7 GHz) in transient mode (CW microwave source) equipped with a Bruker MD5 dielectric ring resonator. Samples were photoexcited through the top of the sample holder with a 532-nm CW laser module (DPGL-2100, SUWTECH LASER) via a 7-meter-long optical fiber with a 1.0 mm diameter (QMMF-UVVIS-1000, OZ Optics, Ottawa, Ontario, Canada). The optical power at the fiber output was approximately 1.0 mW.

The samples were dissolved in toluene (~0.2 mM) and loaded into a 4 mm outer-diameter quartz tube for measurements. Measurements were conducted at 140 K, rather than 80 K, to ensure an observable EPR signal decay upon switching off the excitation source.

Theoretical Calculation

Density function theory (DFT) and time-dependent density function theory (TDDFT) calculations were carried out for structural optimization, optical excitation energies, and emission energies of the reported compounds at the ωB97X-D/6-31G** level of theory by using the Gaussian 16 program package. The optimized structures were confirmed as true minima by vibrational frequency analysis, showing no imaginary frequencies.

Restricted active space with double spin-flip (RAS-2SF) was calculated with the 6-31G** basis sets. RAS-2SF requires a high-spin reference, and therefore, the quintet restricted open-shell Hartree-Fock was used here. We used four active electrons and four active orbitals (4e/4o) in RAS-SF calculations because singlet fission involves the highest occupied and lowest unoccupied molecular orbitals of the donor and acceptor. The setting was verified by using different numbers of active orbitals and electrons, and insignificant changes in excitation energies for the relevant states were seen (see Table S5). All calculations reported in the present work were performed with the developmental version of Q-Chem 6.1.

It is noted that while the RAS-2SF approach can describe qualitatively correct adiabatic wavefunctions and interstate couplings, its description of dynamic correlation is incomplete, leading to a quantitative overestimation of absolute excitation energies.[7] To refine these energies, we computed the excitation energies of the singlet (S_1S_0) and isolated triplet (T_1S_0) states using at the ωB97X-D/6-31G** level of theory. Specifically, these two states were evaluated with spin-restricted TDDFT (RTDDFT), spin-unrestricted DFT (UDFT), respectively. The multiexciton states [^{1}TT and ^{3}TT] were further characterized using broken-symmetry DFT (BS-DFT). This DFT-based protocol, with carefully chosen exchange–correlation functionals, has proven effective for modeling singlet fission systems.[8]

To simulate the experimentally observed SF rate from a theoretical perspective, the electronic coupling (in eV) between the S_1S_0 and ^{1}TT states is required, which can then be used in combination with Fermi's golden rule to

estimate the transition rate. Although the RAS-2SF approach provides interstate electronic couplings via one-particle transition-density matrices ($\|\gamma\|^2$)[9, 10], it does not directly yield the absolute value of the electronic coupling in eV.[11, 12] Therefore, we applied the θ-optimized fragment excitation difference (FED) scheme within the RAS-2SF framework to estimate the SF[13] and triplet–triplet annihilation (TTA) coupling strengths[14], which were then combined with Fermi's golden rule to calculate the corresponding SF and TTA rates.

Section 2. Synthesis

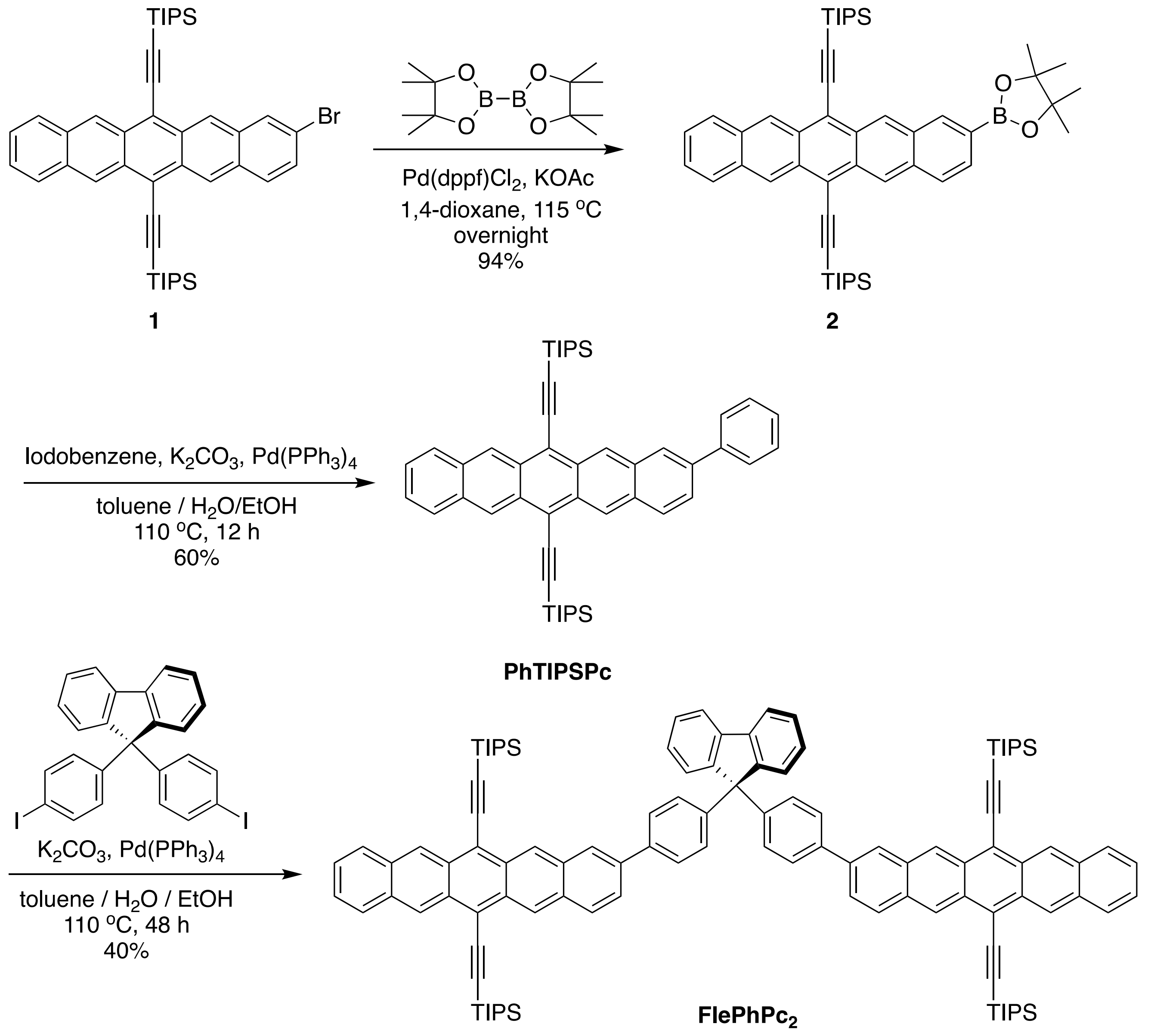

Scheme S1. The synthesis and structures of **PhTIPSPC** and **FlePhPc₂**.

((2-(4,4,5,5-tetramethyl-1,3,2-dioxaborolan-2-yl)pentacene-6,13-diyl)bis(ethyne-2,1-diyl))bis(triisopropylsilane) (2)

Compound **1**[15] (1.436 g, 2.0 mmol), bis(pinacolato)diboron (0.762 g, 3.0 mmol), KOAc (0.589 g, 6.0 mmol) and Pd(dppf)Cl_2 (0.146 g, 0.2 mmol) were placed in a two-neck round bottle. The reaction bottle was evacuated and purged with argon gas, and then 1,4-dioxane (10.0 mL) was added. The reaction was heated under reflux and stirred for 12 hours. After cooling down to the room temperature, the mixture was passed through a short pad of

celite. The filtrate was extracted with CH_2Cl_2, washed with water and brine. The resulting organic layer was dried over anhydrous $MgSO_4$, and concentrated by rotary evaporator to get crude product. The crude was then purified by column chromatography (SiO_2, EtOAc/Hexane = 1/15). Finally, the product was further purified by recrystallization in CH_2Cl_2/MeOH, and compound 2 (1.44 g, 1.9 mmol, 94%) was obtained as a deep blue solid. M.p. (DSC) 142 °C, ^{1}H NMR (400 MHz, $CDCl_3$) δ 9.32 (d, *J* = 4.1 Hz, 2H), 9.30 (s, 1H), 9.26 (s, 1H), 8.51 (s, 1H), 7.97-7.95 (m, 2H), 7.92 (d, *J* = 8.6 Hz, 1H), 7.70 (d, *J* = 8.6 Hz, 1H), 7.42-7.40 (m, 2H), 1.44 (s, 12H), 1.38-1.37 (m, 42H). ^{13}C NMR (101 MHz, CDCl3) δ 137.93, 133.01, 132.32, 131.75, 130.26, 129.58, 128.66, 127.81, 127.50, 126.52, 126.48, 126.02, 125.62, 118.81, 118.23, 107.46, 104.55, 84.07, 24.95, 19.04, 18.99, 11.67. HRMS (m/z, MALDI, $[M]^+$) calcd for $C_{50}H_{65}B_1O_2Si_2$ 764.4616, found 764.4589.

Synthesis of **((2-phenylpentacene-6,13-diyl)bis(ethyne-2,1-diyl))bis(triisopropylsilane) (PhTIPSPc)**

Compound **2**(0.682 g, 0.33 mmol), K_2CO_3 (0.493 g, 1.3 mmol) and $Pd(PPh_3)_4$ (0.103 g, 0.2 mmol)were placed in a two-neck bottle. The two-neck bottle was evacuated and purged with argon gas, and then dry toluene (19 ml), water (5.4 mL) and 95% ethanol (2.7 mL) were added. Then, iodobenzene (0.10 mL, 0.3 mmol) was added into the solution. The reaction was heated under reflux and stirred for 24 hours. After cooling down to the room temperature, the reaction mixture passed through a short pad of celite. The filtrate was extracted with CH_2Cl_2, and washed with water and brine. The resulting organic layer was dried over anhydrous $MgSO_4$, and concentrated by rotary evaporator to get crude product. The crude product was then purified by column chromatography (SiO_2, Hexane). Finally, **PhTIPSPc** (0.14 g, 0.2 mmol, 60%) was obtained as a deep blue solid. M.p. (DSC) 239 °C, ^{1}H NMR (400 MHz, $CDCl_3$) δ 9.34 (s, 1H), 9.31 (s, 3H), 8.14 (s, 1H), 8.06 (d, *J* = 8.9 Hz, 1H), 7.99-7.96 (m, 2H), 7.81 (d, *J* = 7.5 Hz, 2H), 7.71 (dd, *J* = 8.9, 1.6 Hz, 1H), 7.55 (t, *J* = 7.5 Hz, 2H), 7.45-7.41 (m, 3H), 1.37-1.45 (m, 42H). ^{13}C NMR (101 MHz, $CDCl_3$) δ 140.84, 138.32, 132.42, 132.32, 132.28, 131.46, 130.93, 130.74, 130.61, 129.33, 128.97, 128.67, 127.67, 127.27, 126.56, 126.45, 126.32, 126.12, 126.03, 125.88, 118.44, 118.26, 107.26, 107.16, 104.68, 104.65, 19.00, 11.68. HRMS (m/z, MALDI, $[M]^+$) calcd for $C_{50}H_{58}Si_2$ 714.4077, found 714.4536.

Synthesis of **((((9H-fluorene-9,9-diyl)bis(4,1-phenylene))bis(pentacene-2,6,13-triyl))tetrakis(ethyne-2,1-diyl))tetrakis(triisopropylsilane) (FlePhPc2)**

Compound **2** (0.530 g, 0.69 mmol), 9,9-bis(4-iodophenyl)-9H-fluorene[16] (0.188 g, 0.33 mmol), K_2CO_3 (0.182 g, 1.32mmol) and $Pd(PPh_3)_4$ (0.038 g, 0.03 mmol) were placed in a two-neck round bottle. The reaction bottle was evacuated and purged with argon gas, and then dry toluene (7.0 mL), water (2.0 mL) and 95% ethanol (1.0 mL) were added. The reaction was heated under reflux and stirred for 48 hours. After cooling down to the room temperature, the mixture was passed through a short pad of celite. The filtrate was extracted with CH_2Cl_2, washed with water and brine. The resulting organic layer was dried over anhydrous $MgSO_4$, and concentrated by rotary evaporator to get crude product. The crude was then purified by column chromatography (SiO_2, Hexane). Finally, the product was further purified by recrystallization in CH_2Cl_2/MeOH, and **FlePhPc2** (0.210 g, 0.13 mmol, 40%) was obtained as a deep blue solid. M.p. > 407 °C (decomp.). ^{1}H NMR (400 MHz, $CDCl_3$) δ 9.32 (s, 2H), 9.30 (s, 6H), 8.13 (s, 2H), 8.05 (s, 1H), 8.03 (s, 1H), 7.97 (dd, *J* = 6.7, 3.2 Hz, 4H), 7.87 (d, *J* = 7.5 Hz, 2H), 7.74 (s, 2H), 7.72 (s, 3H), 7.69 (d, *J* = 1.6 Hz, 1H), 7.60 (d, *J* = 7.5 Hz, 2H), 7.48-7.45 (m, 5H), 7.43-7.40 (m, 5H), 7.39 (s, 1H), 7.37 (s, 1H), 1.44-1.36 (m, 84H). ^{13}C NMR (101 MHz, $CDCl_3$) δ 151.00, 145.42, 140.27, 139.05, 138.21, 132.41, 132.29, 132.25, 131.44, 130.91, 130.71, 130.58, 129.34, 128.82, 128.66, 127.95, 127.74, 127.09, 126.51, 126.31, 126.24, 126.11, 125.57, 120.38, 118.42, 118.21, 107.24, 107.13, 104.67, 104.62, 65.17, 18.99, 11.15. HRMS (m/z, MALDI, $[M]^+$) calcd for $C_{113}H_{122}Si_4$ 1590.8624, found 1591.8407.

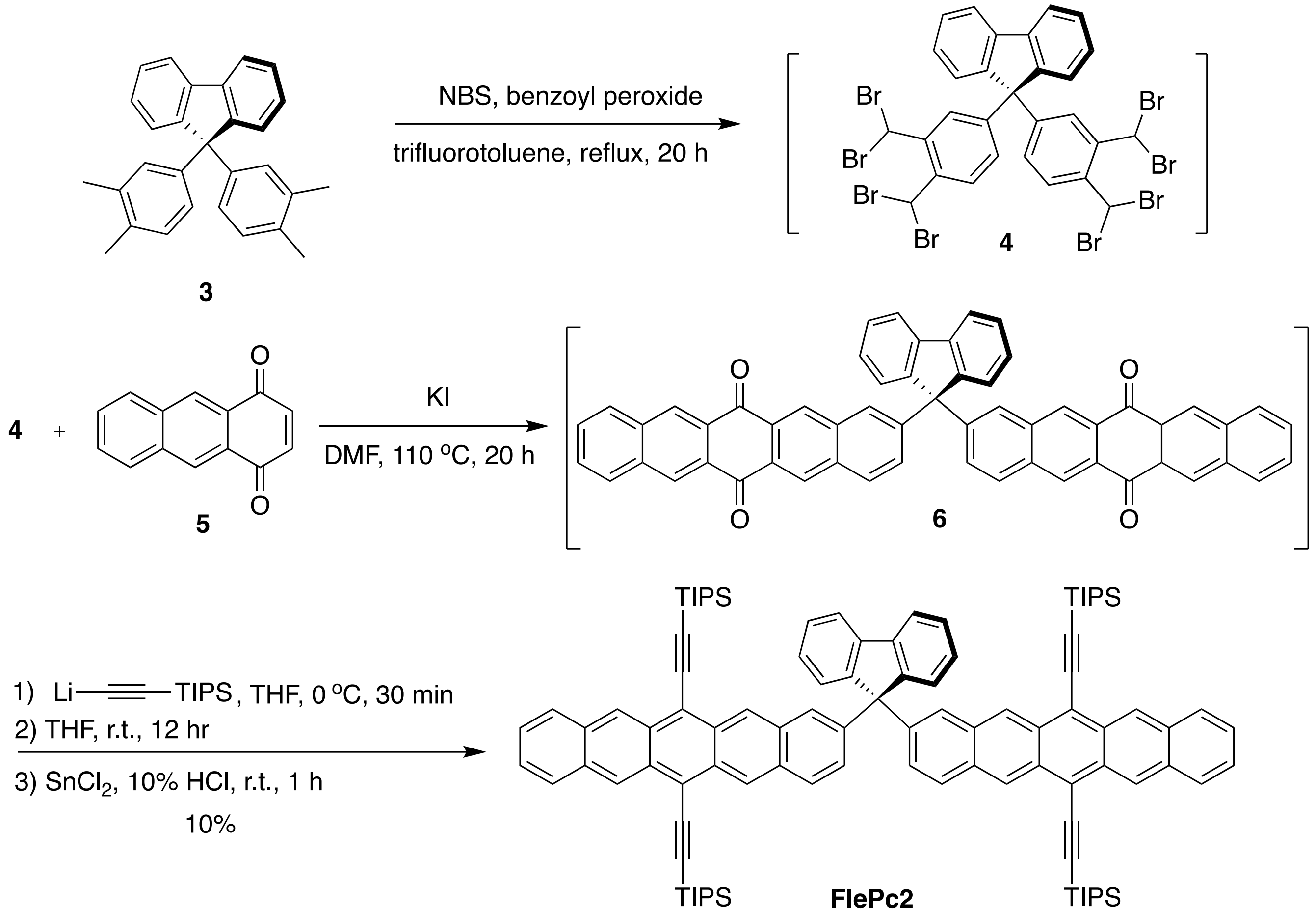

Scheme S2. The synthesis and structure of **FlePc2**

Synthesis of **9,9-bis(3,4-bis(dibromomethyl)phenyl)-9H-fluorene (4)**

Compound **3** (0.300 g, 8.0 mmol), which was prepared according to the reported procedure[17], N-bromosuccinimide (NBS) (0.598 g, 33.6 mmol), and benzoyl peroxide (BPO) (0.134 g, 0.5 mmol) were placed in a two-neck round bottle. The two-neck round bottle was evacuated and purged with argon gas, and then trifluorotoluene (10.0 mL) was added. The solution was refluxed for 4 hours. After slowly cooling to room temperature, added NBS (0.598 g, 33.6 mmol) and BPO (0.134 g, 0.5 mmol) into the reaction. Then, the mixture was refluxed for 20 hours. After slowly returning to room temperature, the precipitated white solid was filtered out. The filtrate was extracted with DCM, and washed with water, 1.0 N NaOH and brine. The resulting organic layer was dried over anhydrous $MgSO_4$, and concentrated by rotary evaporator to get white crude product **2** which was directly taken to the next step without further purification.

Synthesis of **2-(9-(6,13-dioxo-6,6a,12a,13-tetrahydropentacen-2-yl)-9H-fluoren-9-yl)pentacene-6,13-dione (6)**

Compound **4** (0.302 g, 3.0 mmol), compound **5**[18] (0.137 g, 6.6 mmol) and potassium iodide (0.598 g, 36.0 mmol) were placed in a two-neck round bottle. The two-neck round bottle was evacuated and purged with argon gas, and then dry DMF (60 mL) was added. The reaction mixture was heated to 110 °C and stirred for 29 hours. The reaction mixture was cooled down to 0 °C and quenched with water. The resultant solid was collected by filtration, and washed several times with water, acetone and methanol until the filtrate becomes clear. Finally, crude compound **6** was obtained as a brown solid. The product was insoluble in various common organic solvents, making it impossible to measure by NMR spectroscopy.

Synthesis of **(((9H-fluorene-9,9-diyl)bis(pentacene-2,6,13-triyl))tetrakis(ethyne-2,1-diyl))tetrakis(triisopropylsilane) (FlePc2)**

Triisopropylsilylacetylene (1.0 mL, 4.3 mmol) was added into dry THF (12.5 mL) under nitrogen atmosphere. n-BuLi (3.3 ml, 4.3 mmol) was added dropwise to the solution with stirring, and the reaction temperature was kept at 0 °C for 30 minutes. Crude compound **6** (0.300 g, 0.4 mmol) was added and the reaction mixture was slowly warmed to room temperature, and stirred for 15 hours. The solution of tin(II) chloride (0.728 g, 3.8 mmol) in 10% HCl (4.0 mL) was then added dropwise. The color of the reaction mixture changed from brown to deep blue. The solution was stirred for 1 hour at room temperature. Then, the reaction mixture was passed through a short pad of celite. The filtrate was extracted with CH_2Cl_2 and washed with water. The resulting organic layer was dried over anhydrous $MgSO_4$, and concentrated by rotary evaporator to get crude product, which was then purified by column chromatography (SiO_2, Hexane). Finally, the product was further purified by recrystallization in CH_2Cl_2/methanol, and ^{1}H NMR (800 MHz, $CDCl_3$) δ 9.28 (s, 2H), 9.24 (d, *J* = 6.4 Hz, 4H), 9.13 (s, 2H), 7.97-7.96 (m, 4H), 7.93-7.92 (m, 4H), 7.74 (s, 2H), 7.70 (d, *J* = 7.6 Hz, 2H), 7.50-7.46 (m, 4H), 7.41-7.38 (m, 6H), 1.36-1.35 (m, 42H), 1.24-1.23 (m, 42H). ^{13}C NMR (201 MHz, $CDCl_3$) δ 149.91, 141.75, 140.65, 132.22, 131.99, 131.37, 130.88, 130.81, 130.53, 130.38, 128.87, 128.65, 127.97, 127.83, 127.73, 126.57, 126.52, 126.30, 126.26, 126.21, 125.97, 125.68, 120.56, 118.30, 118.26, 107.16, 106.97, 104.65, 104.52, 65.44, 18.96, 18.85, 11.65, 11.58. HRMS (m/z, MALDI, $[M]^+$) calcd for $C_{101}H_{114}Si_4$ 1438.7998, found 1439.8086.

Section 3. Photophysical Measurements

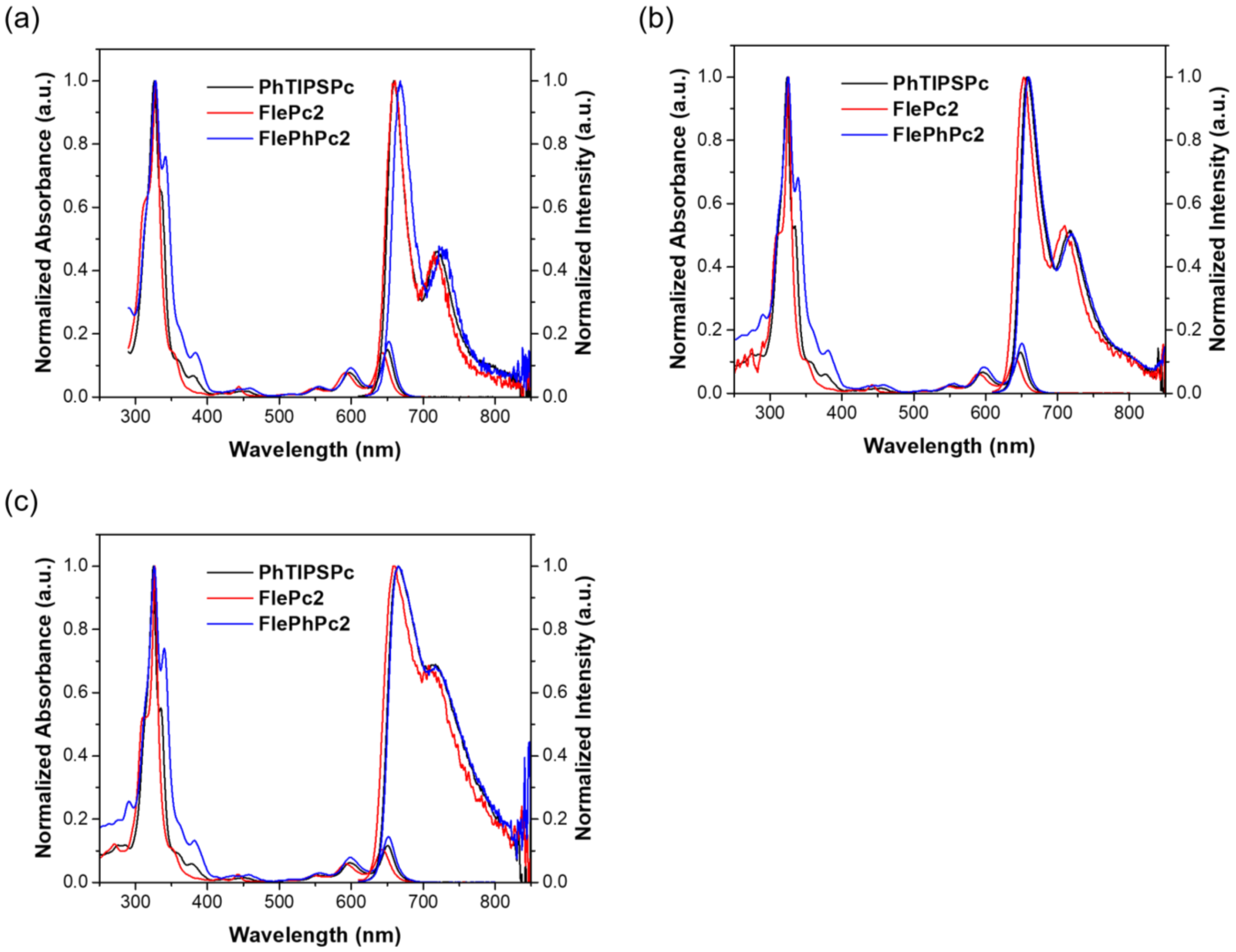

Figure S1. Absorption and emission spectra recorded in (a) Toluene, (b) THF, and (c) DCM for the reported compounds specified in the legend.

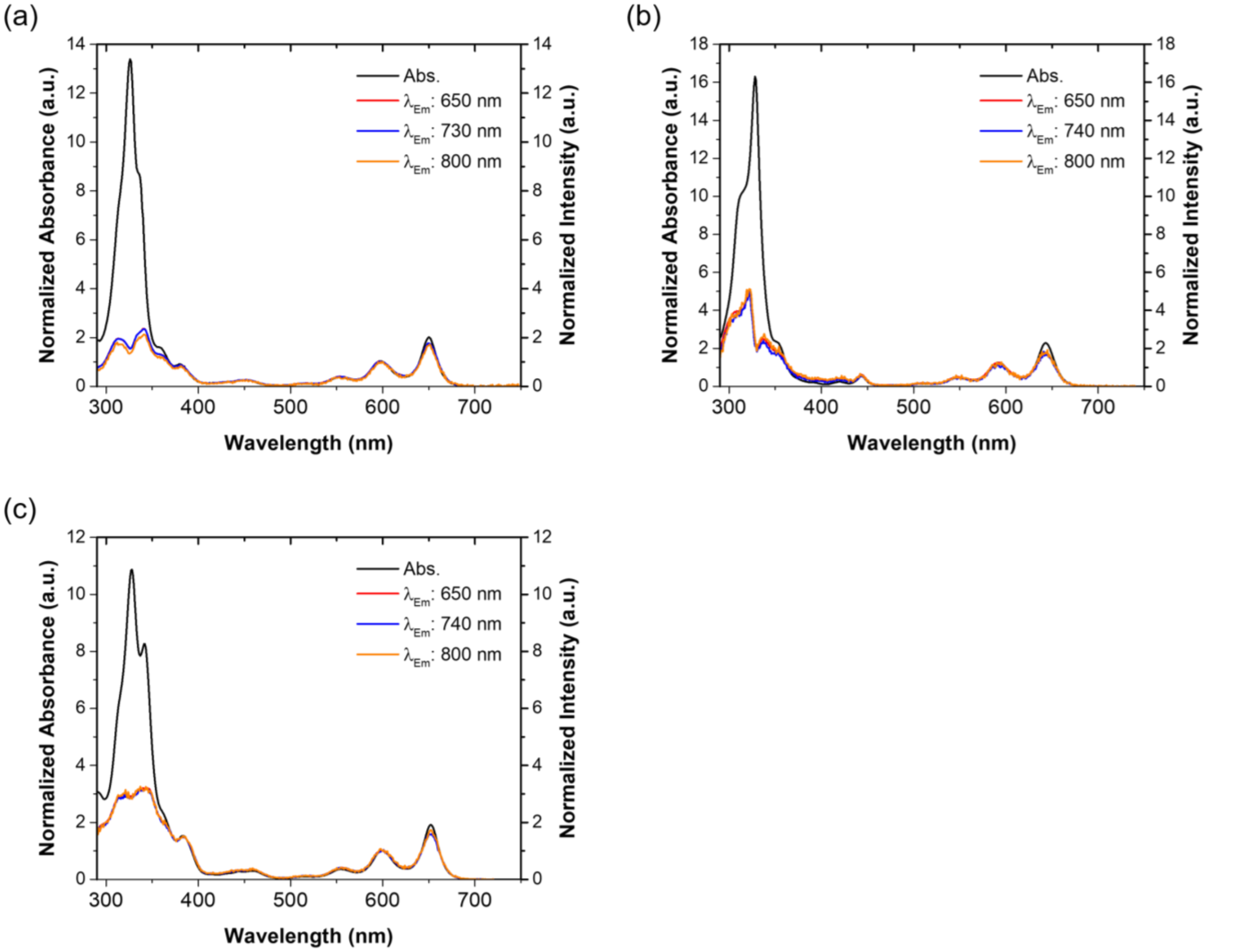

Figure S2. Excitation spectra for (a) **PhTIPSPc**, (b) **FlePc2**, and (c) **FlePhPc2** recorded in toluene. All spectra were normalized at their lowest-lying absorption band at 600 nm. "λ_{Em}" represents the monitored wavelength while conducting the excitation profile.

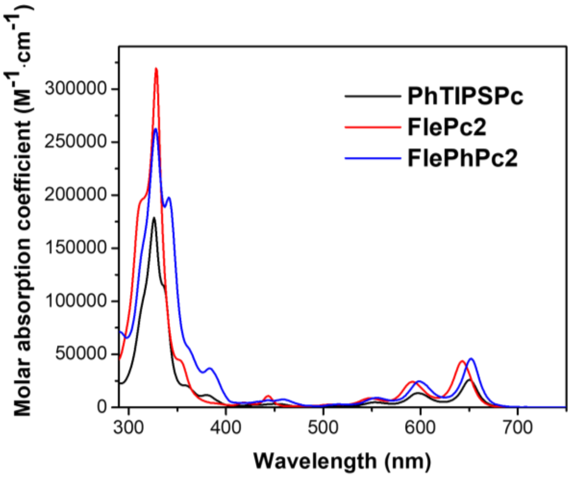

Figure S3. Molar absorption coefficients for the reported compounds recorded in toluene.

Table S1. Photophysical data for the reported compounds.

Molecule	Solvent	$\lambda_{abs}(\varepsilon)$/ nm ($M^{-1}cm^{-1}$) [a]	λ_{em}/ nm [a]	QY/ % [a, b]
**PhTIPSPc**	Tol	553 (4832), 596 (13354), 650 (25963)	659, 719	17.7
	THF	552, 596, 648	658, 716	10.9
	DCM	554, 597, 650	666, 717	8.6
**FlePc2**	Tol	548 (8700), 592 (24044), 643 (43634)	661, 717	0.4
	THF	547, 590, 641	653, 710	0.3
	DCM	549, 592, 643	659, 708	0.2
**FlePhPc2**	Tol	555 (8963), 599 (24605), 652 (45948)	668, 722	12.4
	THF	556, 598, 650	659, 721	10.2
	DCM	556, 599, 652	665, 716	7.2

a. UV-vis spectra, PL spectra, and quantum yields were recorded in toluene with a concentration of 10^{-5} M at 25 °C.

b. LDS 821 in DCM (QY=12.1%) was employed as the standard.

Table S2. HOMO and LUMO energy levels (in eV) of pentacene and fluorene calculated at the B3LYP/6-311G(d,p) level of theory.

Molecule		Energy (eV)
Pentacene	HOMO	-4.861
	LUMO	-2.654
Fluorene	HOMO	-6.004
	LUMO	-1.029

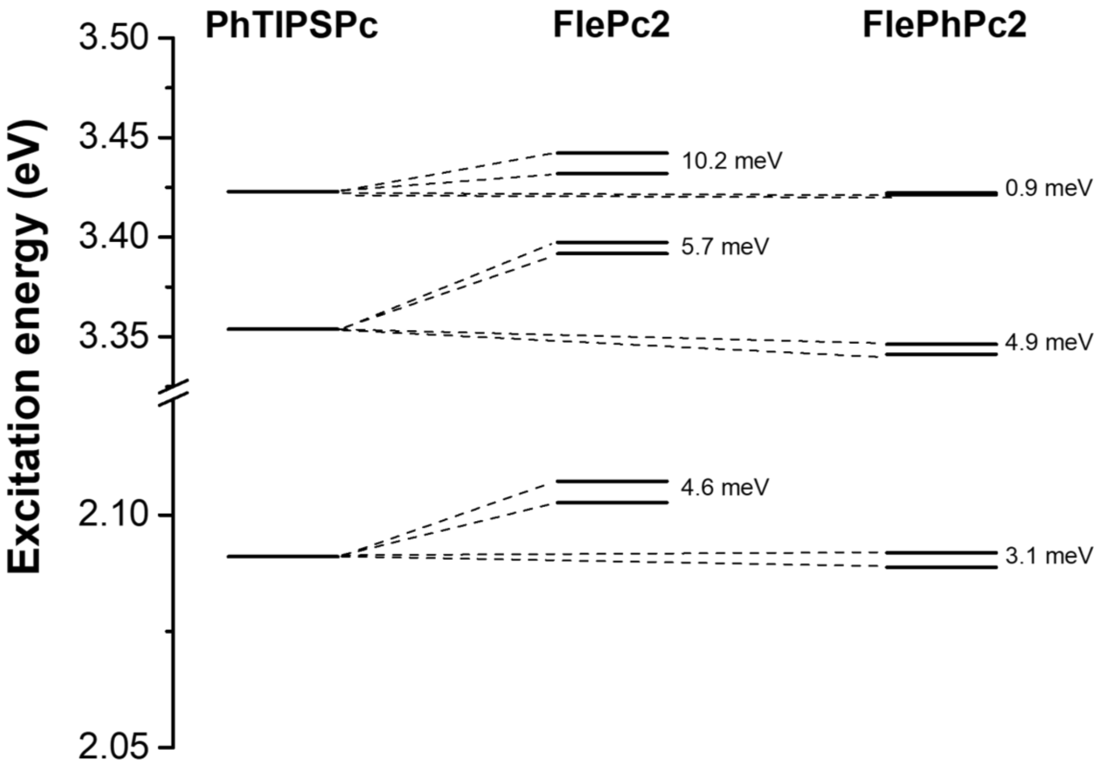

Figure S4. Energy-level diagrams of the calculated singlet excited states at optimized ground-state geometries for **PhTIPSPc**, **FlePc2**, and **FlePhPc2**. Dashed lines indicate Davydov splitting, and the associated energy values (in meV) are shown next to the corresponding states.

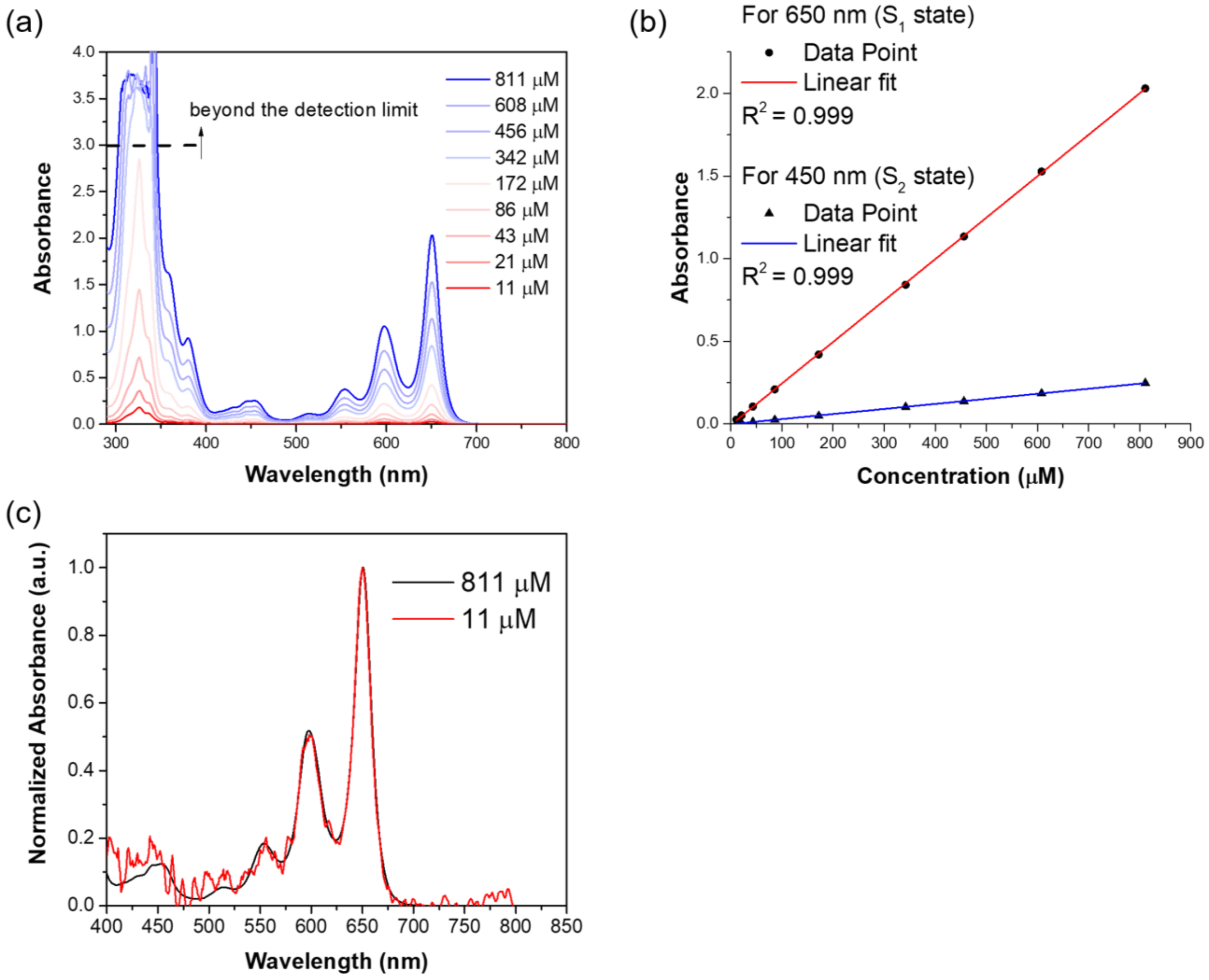

Figure S5. (a) Absorption spectra of **PhTIPSPc** in toluene at various concentrations, as indicated in the legend. Notably, the concentrations used here are two times higher than those of the dimers (see Figures S6–S7), ensuring that the total number of pentacene units in solution is comparable across samples. (b) Linear correlation between concentration and absorbance at the monitoring wavelengths corresponding to the S_1 state (red line) and the S_2 state (blue line). (c) Normalized absorption profiles at the S_1 transition, showing consistent spectral shapes between the diluted and concentrated ones.

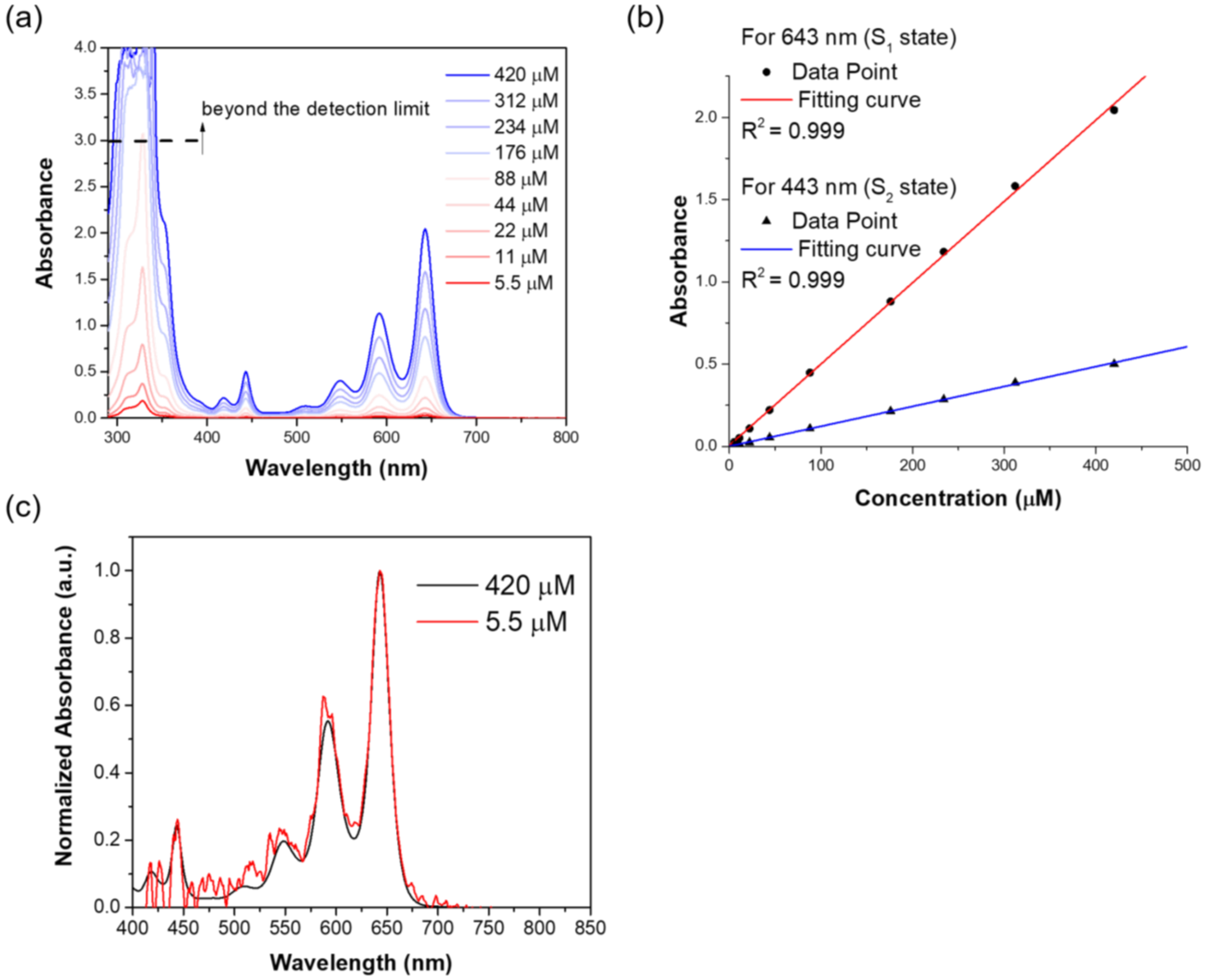

Figure S6. (a) Absorption spectra of **FlePc2** in toluene at various concentrations, as indicated in the legend. (b) Linear correlation between concentration and absorbance at the monitoring wavelengths corresponding to the S_1 state (red line) and the S_2 state (blue line). (c) Normalized absorption profiles at the S_1 transition, showing consistent spectral shapes between the diluted and concentrated ones.

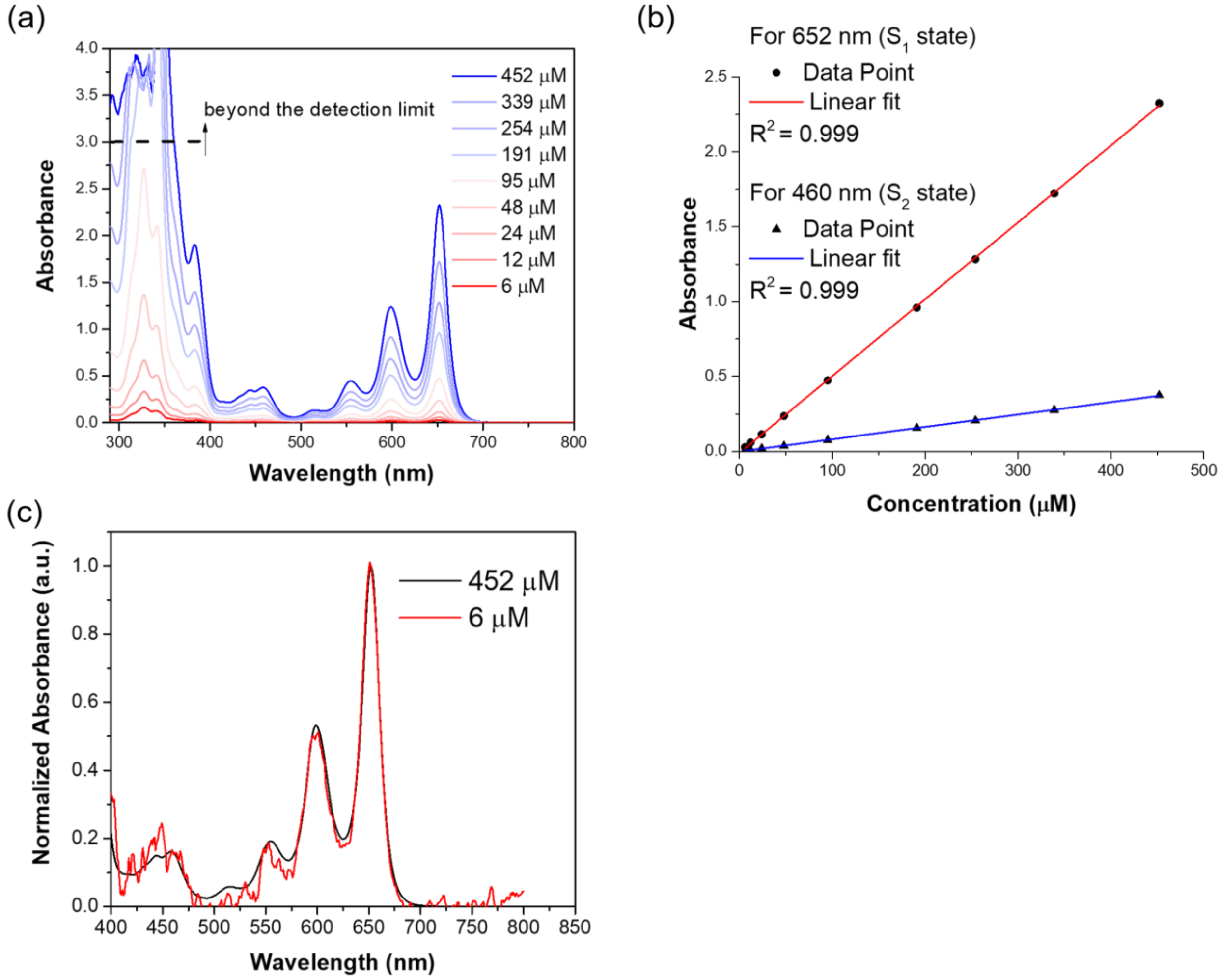

Figure S7. (a) Absorption spectra of **FlePhPc2** in toluene at various concentrations, as indicated in the legend. (b) Linear correlation between concentration and absorbance at the monitoring wavelengths corresponding to the S_1 state (red line) and the S_2 state (blue line). (c) Normalized absorption profiles at the S_1 transition, showing consistent spectral shapes between the diluted and concentrated ones.

Discussion on the Origin of the Long-Lived Fluorescence Component in FlePc2

Because the long-lived lifetime (~10 ns) component of **FlePc2** is nearly identical to that of **PhTIPSPc**, it could initially be attributed to residual S_1S_0 excitons that bypass SF. However, this assignment can be excluded based on both quantitative and spectroscopic evidence. The long-lived component observed in the fluorescence decay of **FlePc2** (Figure 4c) accounts for only ~10% of the total emission amplitude, implying that over 90% of the singlet population undergoes SF. If the remaining minor fraction (~10%) of un-fissioned S_1S_0 state truly existed, distinct spectral signatures from this state should be observable in the fs-TA spectra. In contrast, the fs-TA data (Figure 4d) display a clear precursor-successor relationship between the decay of the S_1S_0 excited-state absorption and the rise of the correlated ^1TT state (Figure 5c and 5d). No independent spectral features assignable to un-fissioned S_1S_0 excitons were detected, thereby ruling out the presence of a minor fraction (~10%) of residual S_1S_0 population.

In this study, we attribute the long-lived fluorescence component to triplet-triplet annihilation (TTA), even though TTA in TIPS-pentacene is endothermic and, therefore, thermodynamically disfavored. Similar delayed fluorescence attributed to thermally activated TTA has been reported in pentacene-based singlet-fission dimers[19, 20], where an extended S_1 population emerges at elevated temperatures.[21] Such behavior implies that the correlated triplet-pair state requires additional thermal energy to overcome the energetic barrier associated with TTA. To further substantiate the assignment of the long-lived component to TTA, we performed both magnetic-field-dependent and temperature-dependent lifetime measurements (Figure S13). The observed modulation of the delayed fluorescence intensity and lifetime with both magnetic field and temperature provides clear evidence that the two T_1 excitons interact and that the recombination dynamics are governed by TTA.

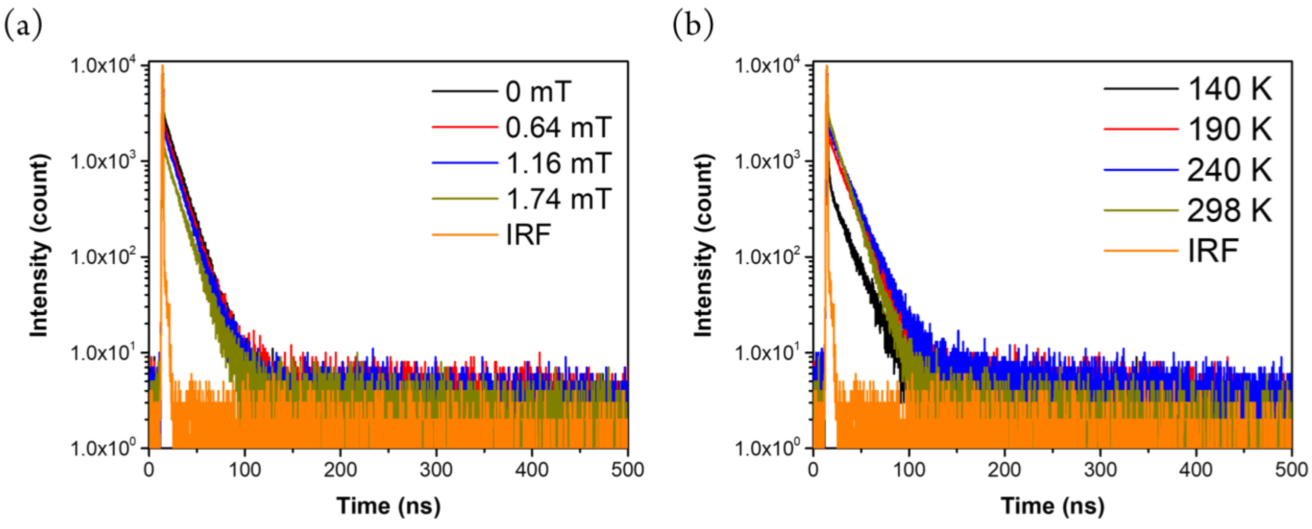

Figure S8. (a) Magnetic-field-dependent conducted at 298K and (b) temperature-dependent lifetime measurements of the delayed fluorescence attributed to TTA.

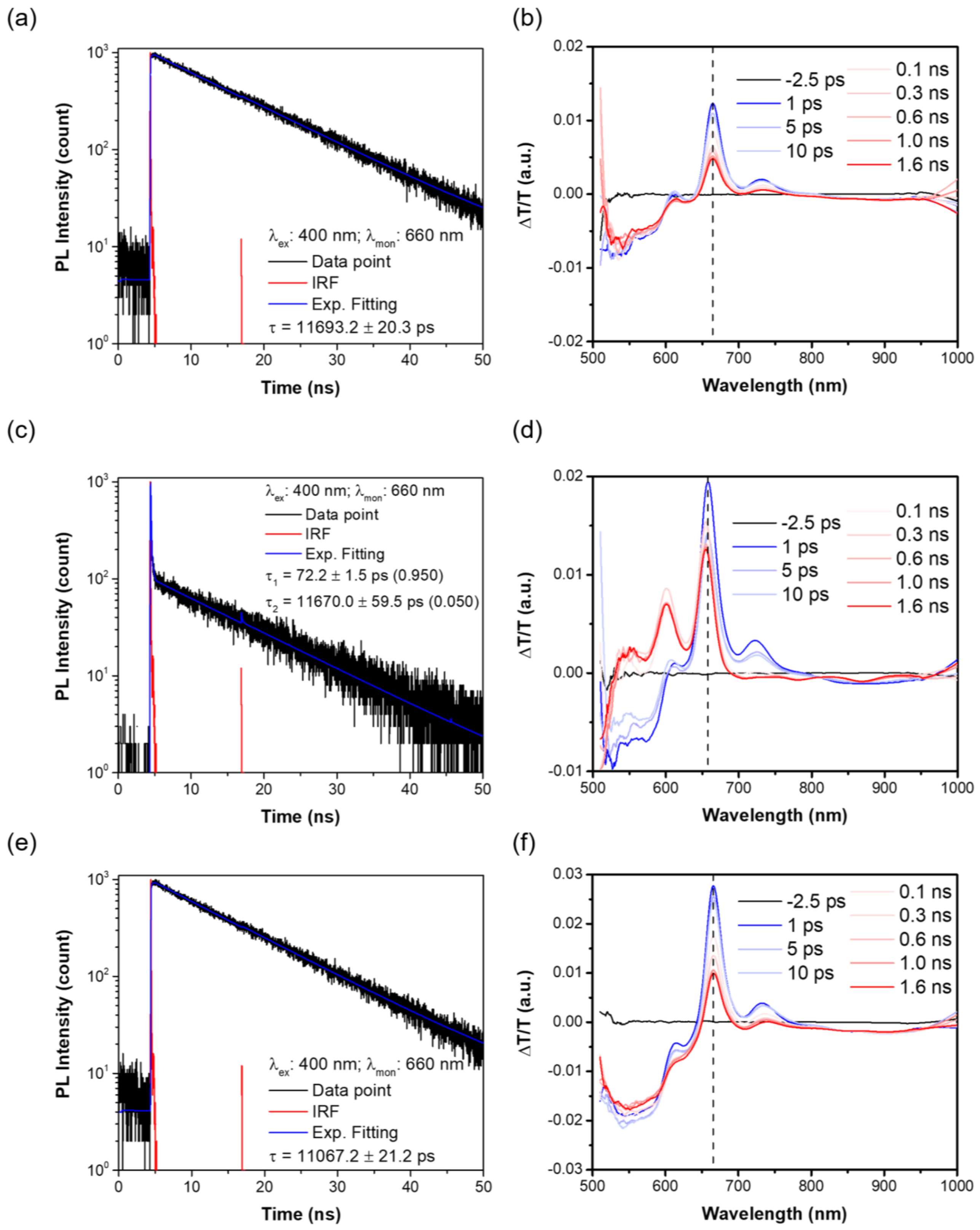

Figure S9. Fluorescence lifetime measurements (a, c, e) conducted at ~10 μM and fs-TA measurements (b, d, f) conducted at ~100 μM in THF for **PhTIPSPc**, **FlePc2**, and **FlePhPc2**, respectively. All the measurements were conducted at ~298K.

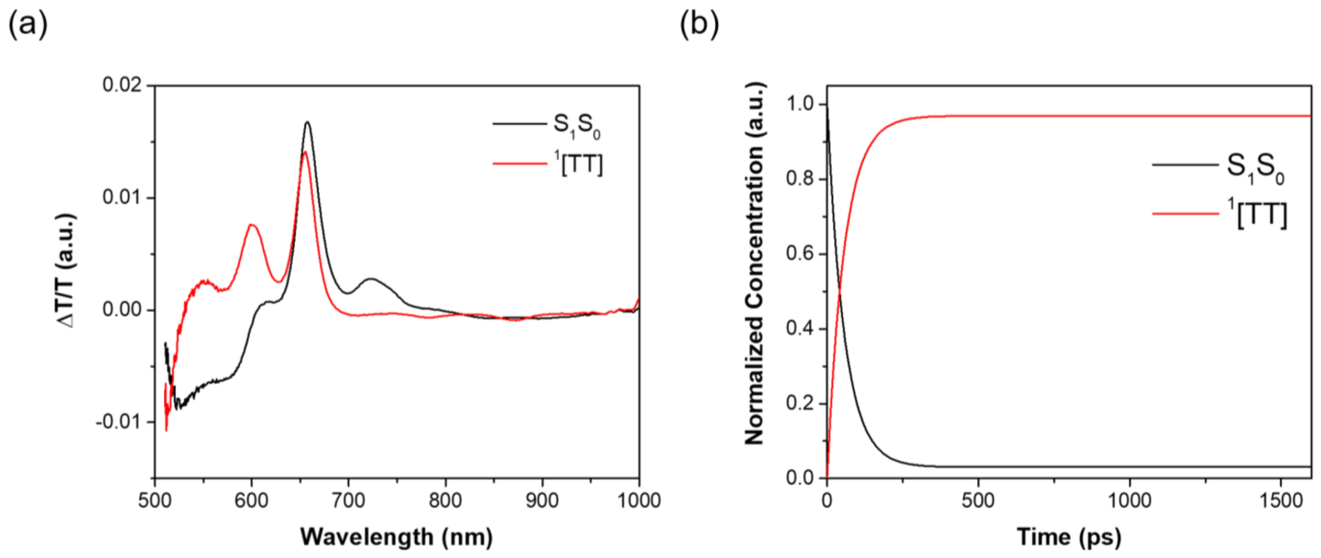

Figure S10. (a) and (b) present the results of global analysis of fs-TA spectra for **FlePc2** in THF, based on the simplified kinetic model shown in Figure 5(e). (a) displays the species-associated spectra, while (b) shows the corresponding time-dependent concentration profiles.

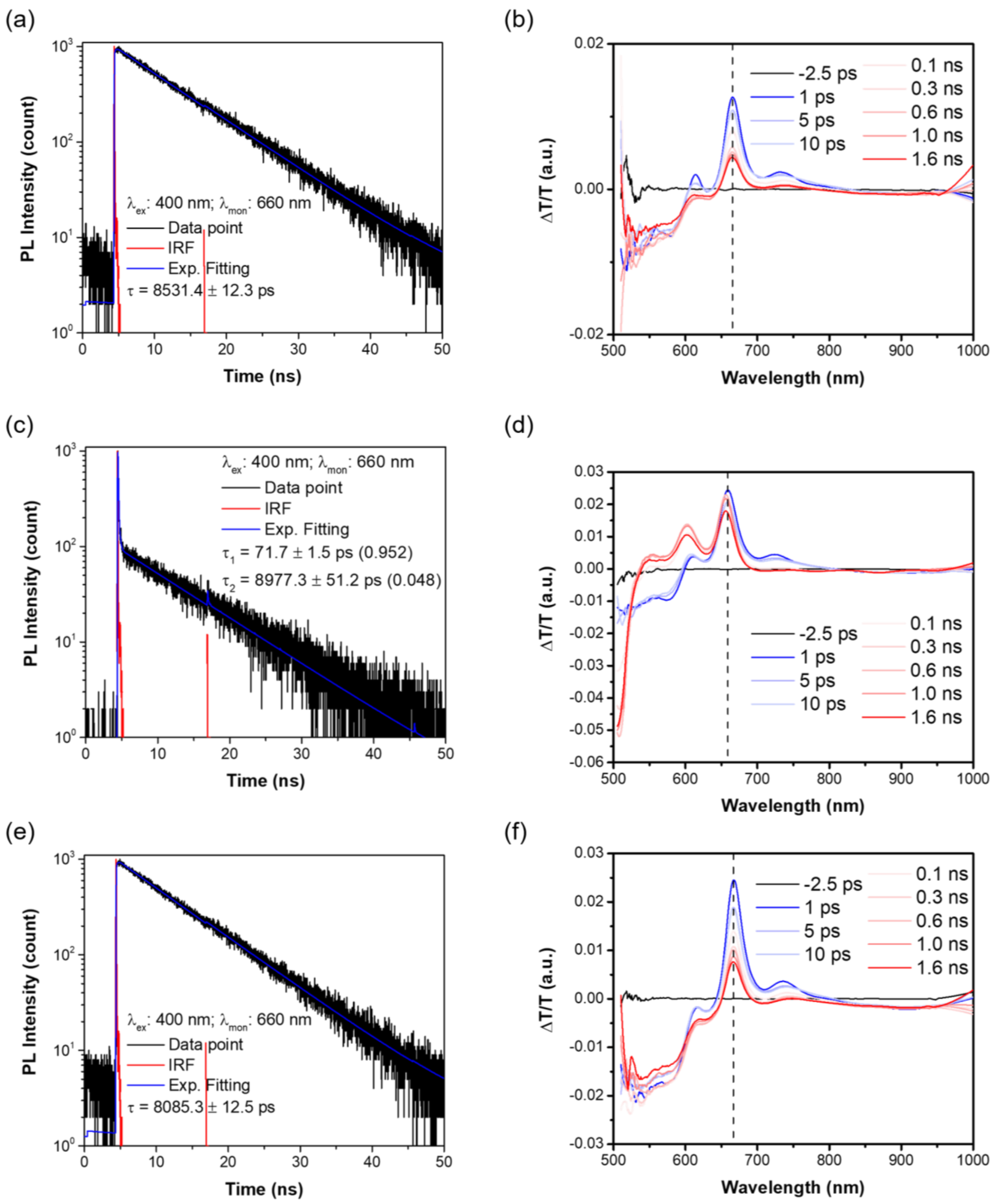

Figure S11. Fluorescence lifetime measurements (a, c, e) conducted at ~10 μM and fs-TA measurements (b, d, f) conducted at ~100 μM in DCM for **PhTIPSPc**, **FlePc2**, and **FlePhPc2**, respectively. All the measurements were conducted at ~298K.

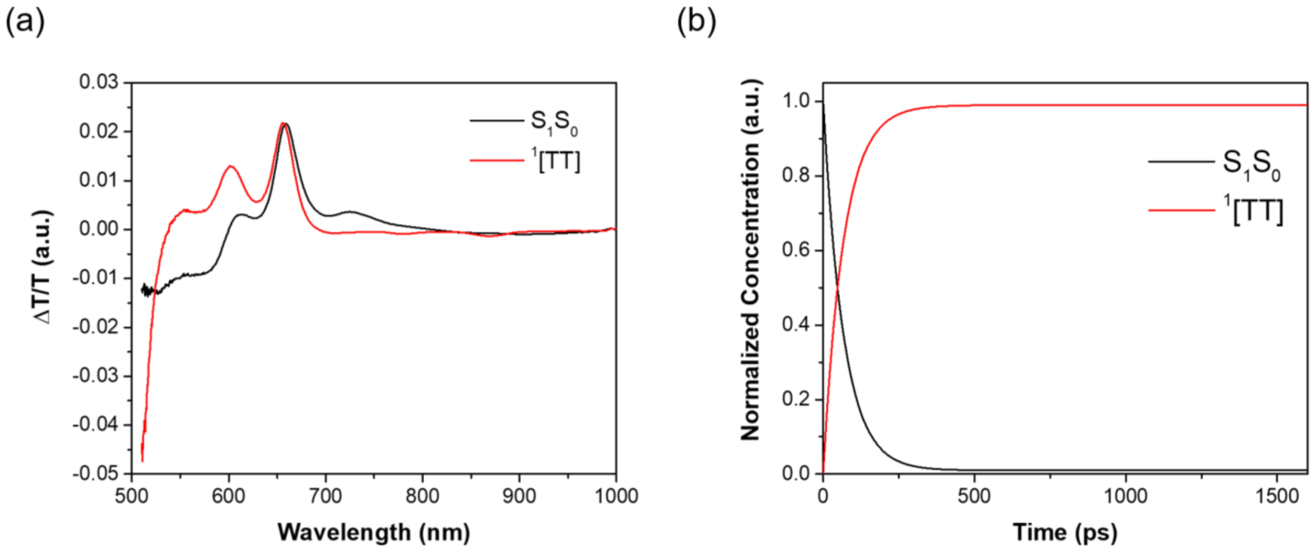

Figure S12. (a) and (b) present the results of global analysis of fs-TA spectra for **FlePc2** in DCM, based on the simplified kinetic model shown in Figure 5(e). (a) displays the species-associated spectra, while (b) shows the corresponding time-dependent concentration profiles.

Table S3. Kinetic parameters derived from global analysis of the fs-TA data for **FlePc2**, based on the simplified kinetic model shown in Figure 5(e).

Solvent	k_{fiss} ($\times10^{10}$ s^{-1})	k_{fus} ($\times10^{8}$ s^{-1})	K_{eq}
Tol	1.24 ± 0.03	0.914 ± 0.002	136.0 ± 3.3
THF	1.98 ± 0.05	4.88 ± 0.03	40.6 ± 1.0
DCM	1.47 ± 0.04	1.46 ± 0.04	101.1 ± 3.9

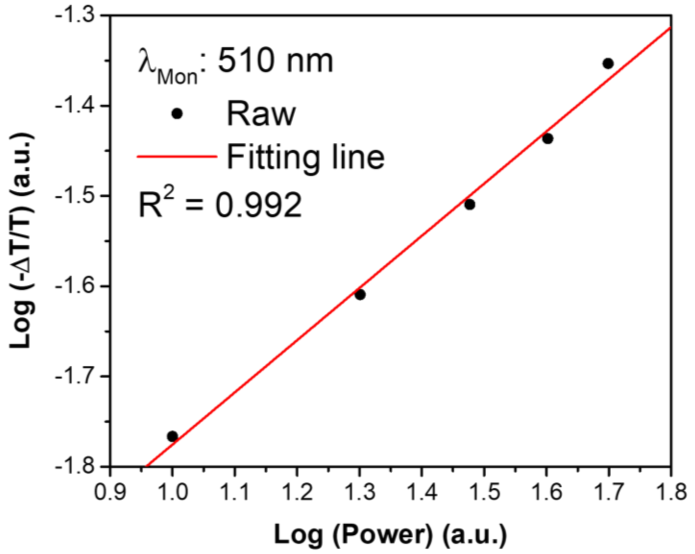

Figure S13. Power dependence of the triplet pair signal of **FlePc2** at 510 nm in fs-TA measurements. The measurements were conducted at ~298K.

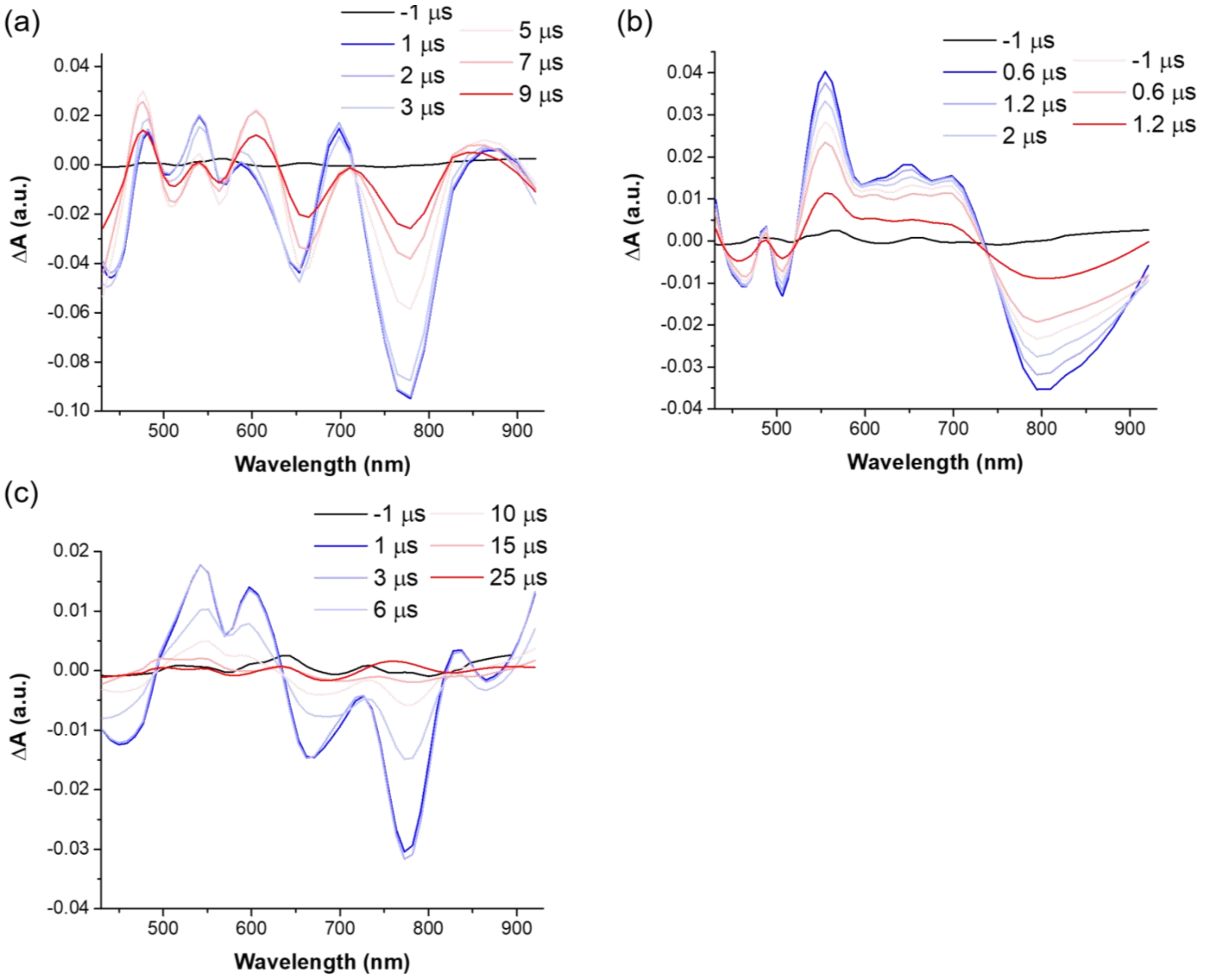

Figure S14. Two-dimensional nanosecond transient absorption spectra of (a) **PhTIPSPc**, (b) **FlePc2**, and (c) **FlePhPc2** in degassed toluene (~100 μM). Notably, the positive absorption signal observed in 510–520 nm region is attributed to the T_1 state of pentacene, as discussed in the main text. All the measurements were conducted at ~298K.

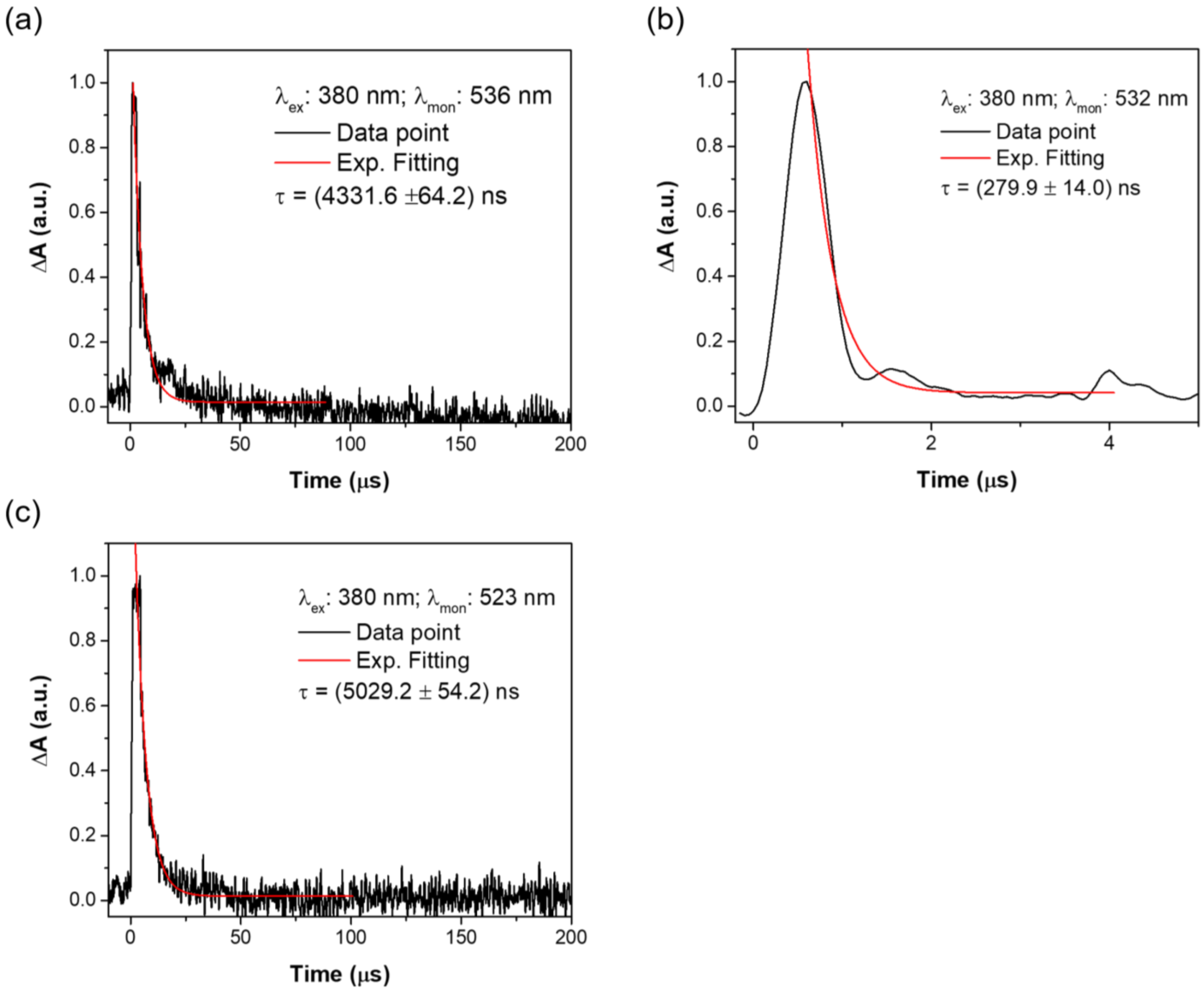

Figure S15. Selected single-wavelength kinetic traces obtained from ns-TA spectroscopy, monitored at the characteristic T_1 state absorption wavelength for (a) **PhTIPSPc**, (b) **FlePc2**, and (c) **FlePhPc2** in degassed toluene (~100 μM). All the measurements were conducted at ~298K.

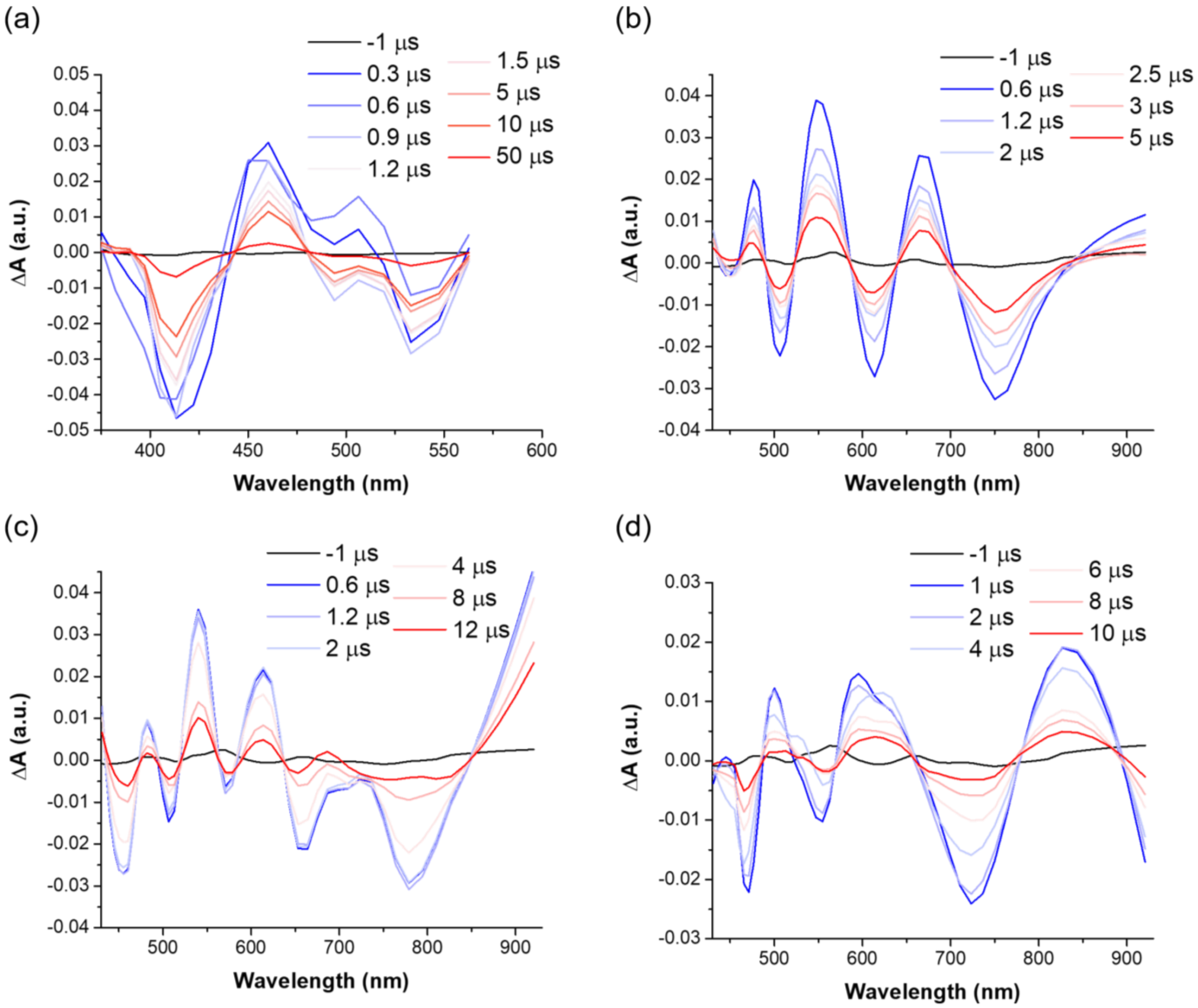

Figure S16. Two-dimensional nanosecond transient absorption spectra of (a) **PtOEP** and sensitization experiments of (b) **PhTIPSPc**, (c) **FlePc2**, and (d) **FlePhPc2** in degassed toluene. In the sensitization experiments, the concentration of PtOEP was 500 μM, while that of each reported compound was 100 μM. Notably, the ns-TA spectra of the reported compounds differ significantly from that of PtOEP alone, confirming the occurrence of the energy transfer process. The absorption feature observed at 510–520 nm is attributed to the T_1 state of pentacene, as discussed in the main text. All the measurements were conducted at ~298K.

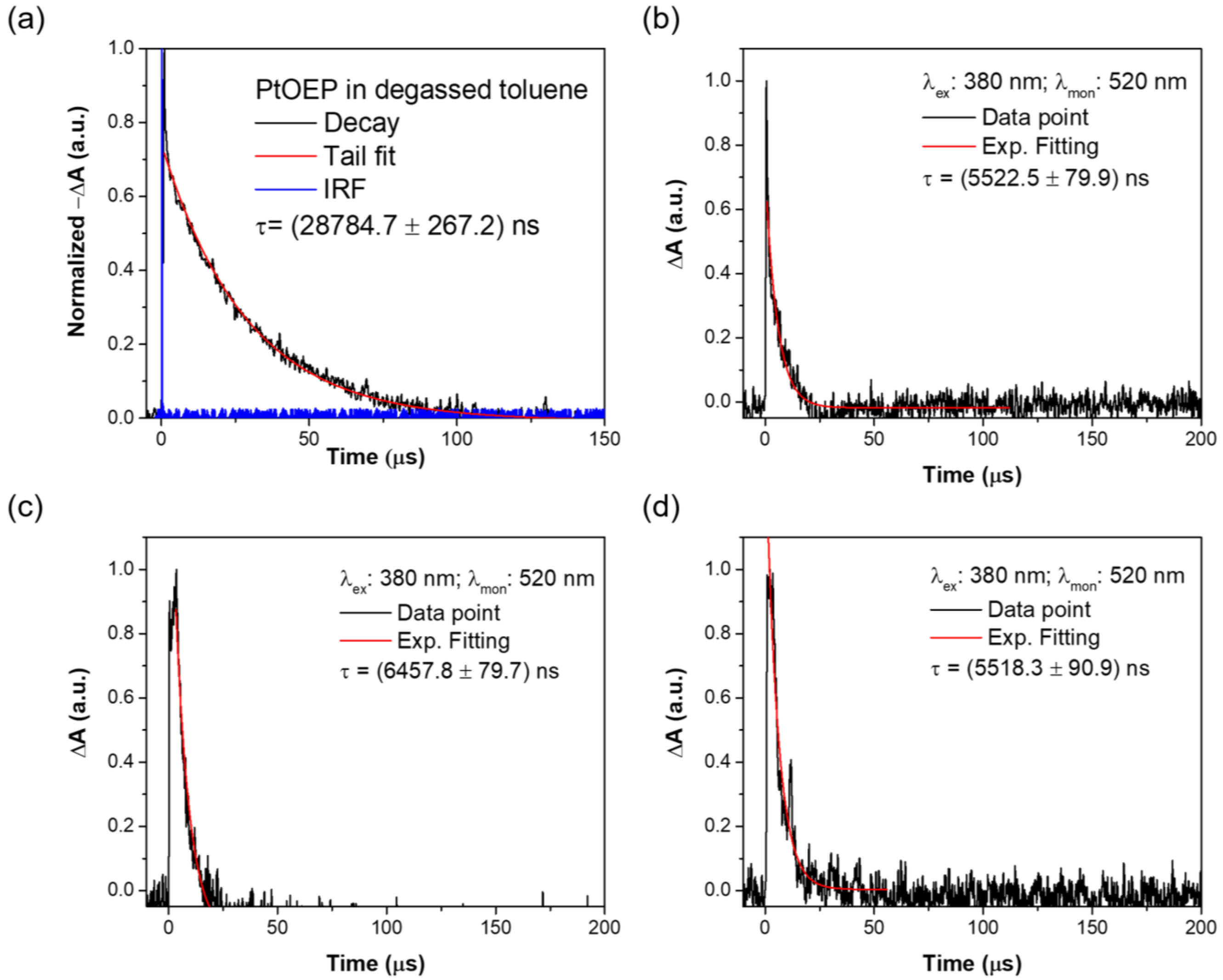

Figure S17. Selected single-wavelength kinetic traces obtained from ns-TA spectroscopy, monitored at the characteristic T_1 state absorption wavelength of (a) PtOEP and sensitization experiment of (b) **PhTIPSPc**, (c) **FlePc2**, and (d) **FlePhPc2** in degassed toluene. Notably, the positive absorption signal observed in 510–520 nm region is attributed to the T_1 state of pentacene, as discussed in the main text. All the measurements were conducted at ~298K.

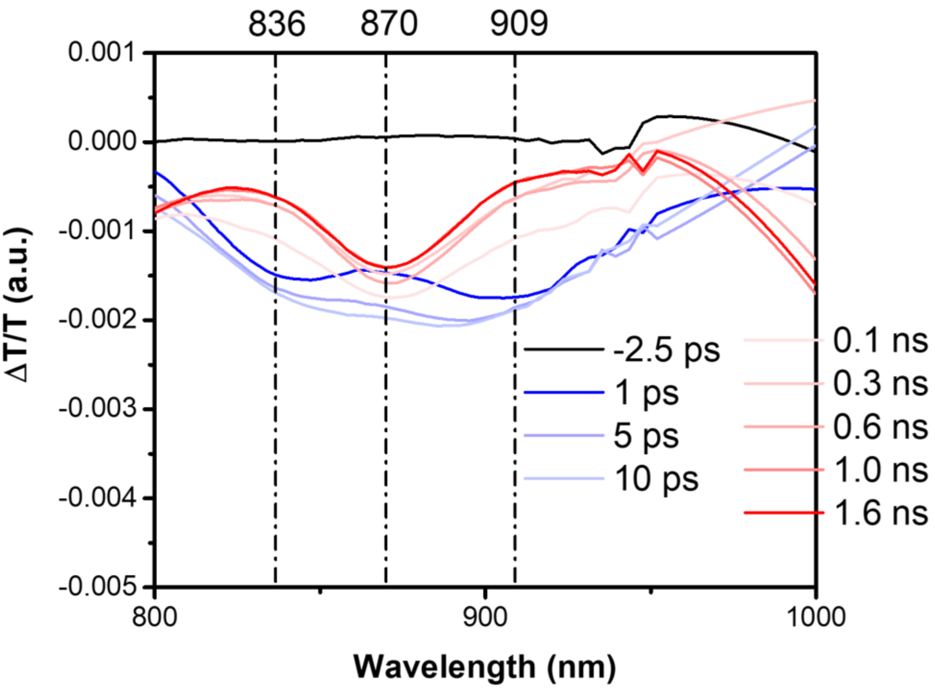

Figure S18. Fs-TA measurements of **FlePc2** performed in toluene at a concentration of ~100 μM, with selected probe wavelengths labeled in the figure. Notably, no spectral signatures characteristic of pentacene radical anions or cations were observed[22, 23], indicating that inter- or intramolecular electron transfer does not occur during the SF process. The measurements were conducted at ~298K.

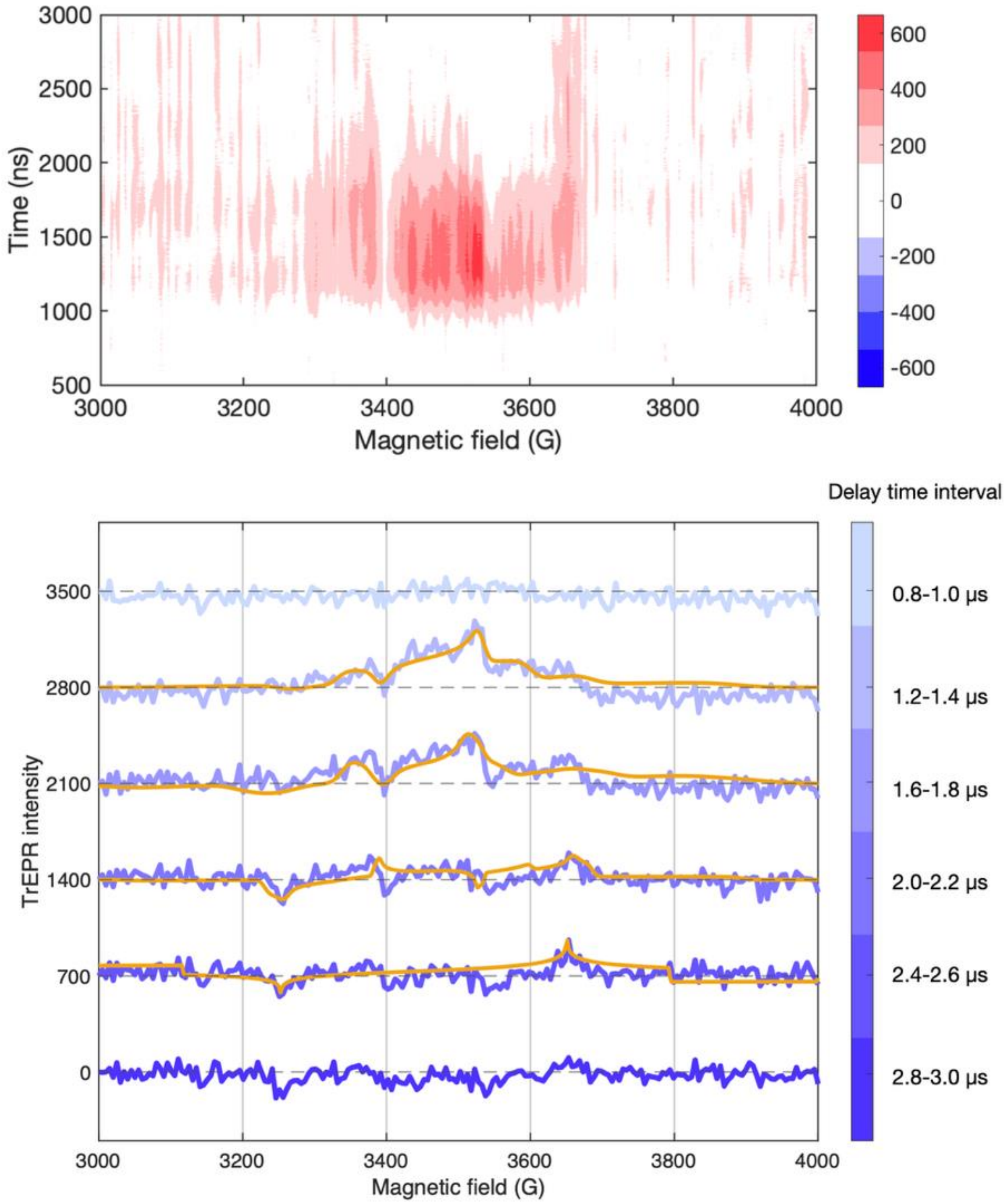

Figure S19. Continuous-wave transient EPR (trEPR) of **FlePc2** (~0.2 mM in toluene) shows early-time dominance of the correlated quintet ^{5}TT with a delayed growth of a weaker ^{3}TT component. **Top:** Contour plot of X-band (9.7 GHz) trEPR spectra recorded at 140 K. **Bottom:** trEPR spectra averaged over selected delay windows; experimental traces (blue) are overlaid with simulations (orange). The early spectra (1.2–1.4 and 1.6–1.8 μs) display the characteristic ^{5}TT pattern, confirming quintet formation with a lifetime of at least ~0.6 μs at 140 K. Quantitative fits yield ^{3}TT/^{5}TT ratios of 0, 0.06, and 0.24 for the 1.2–1.4, 1.6–1.8, and 2.0–2.2 μs windows, corresponding to 0% and ~6% ^{3}TT contributions at early times and a ~19% ^{3}TT contribution at later times; the 2.4–2.6 μs spectrum is well reproduced by ^{3}TT alone.

We note that trEPR was performed at 140 K using polarization-sensitive CW detection, whereas FS-ESE (shown in Figures 6 and 7) was measured at 80 K in a frozen glass; the higher temperature and different detection modality can reveal a small thermally activated ^{3}TT leakage channel that is negligible and not echo-detectable under the FS-ESE conditions. Thus, both methods consistently indicate that the initial spin-active photoproduct is predominantly ^{5}TT, with dissociation or relaxation into lower-multiplicity triplet states strongly suppressed. Because trEPR has lower signal-to-noise than FS-ESE, the fits are less constrained; uncertainties in the extracted ^{3}TT fractions are estimated to be ~5%. Simulations use the same Hamiltonian framework as FS-ESE with slightly adjusted quintet ZFS parameters, (D, E) = (1200, 50) MHz; relative quintet sublevel populations are [0, 0, 0.16, 0.30, 0.54] for Q_{+2} to Q_{-2}, and triplet polarizations are [0, 0.2, 0.8] for T_{+1} to T_{-1}.

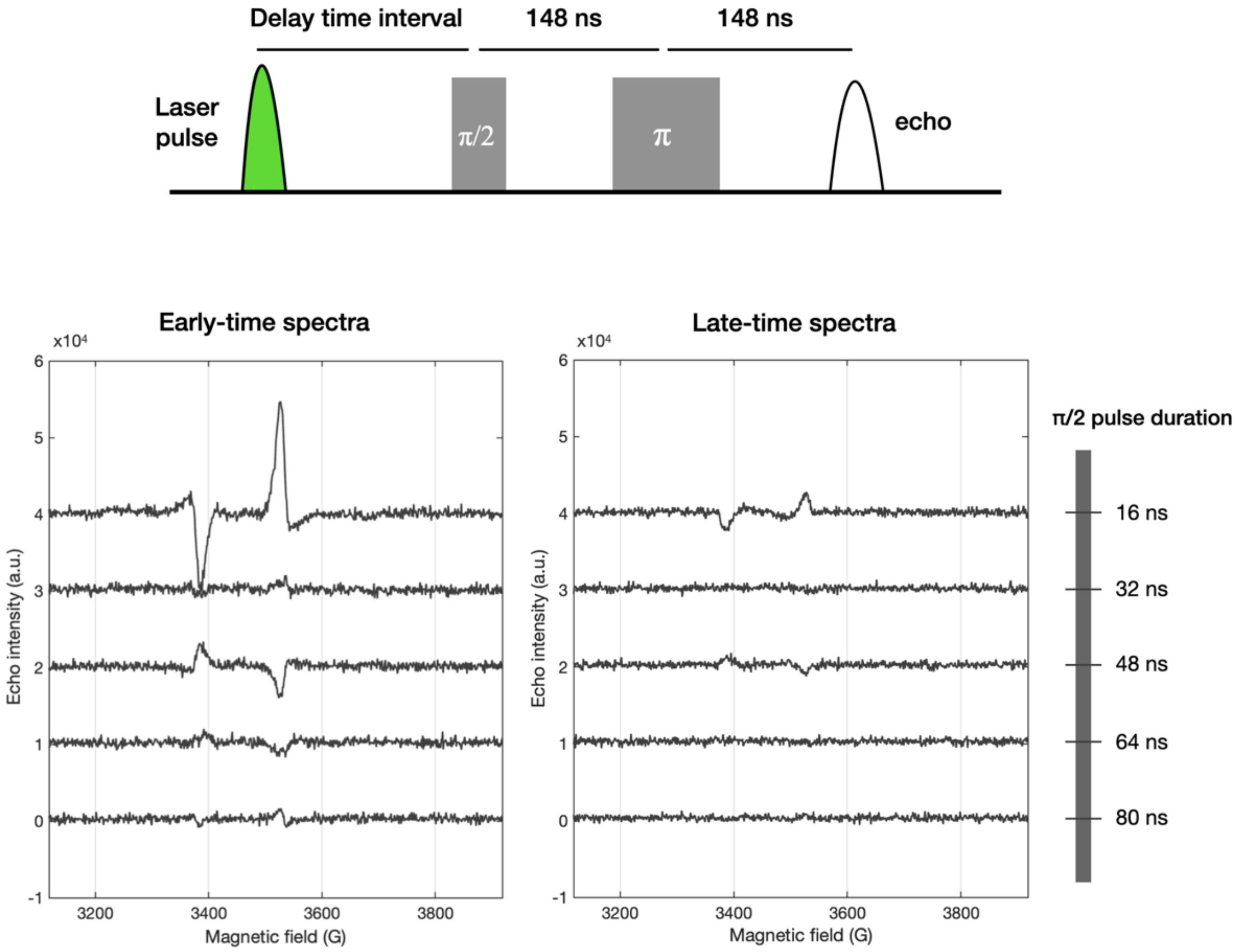

Figure S20. Transient FS-ESE measurements of **FlePc2** (0.3 mM in toluene) at 140 K confirm that the spin-echo signal is dominated by ^{5}TT and show no detectable triplet-echo contribution. **Top:** Pulse sequence for transient echo detection with variable delay time intervals and π/2 pulse lengths. **Bottom:** Transient FS-ESE spectra collected in the early (1.2–1.4 μs) and later (2.0–2.2 μs) time windows corresponding to those analyzed in Figure S19. In both windows, the spectra display only the characteristic ^{5}TT transitions, consistent with quintet

formation and the sublevel-population evolution inferred from trEPR. To test for a potential minor triplet states (^{3}TT or T_1), FS-ESE scans were repeated using a series of $\pi/2$ pulse lengths; because triplets and quintets have distinct Rabi frequencies, this protocol enhances sensitivity to triplet echoes if present. Aside from the expected pulse-length-dependent modulation of the quintet peaks, no additional features attributable to triplet states were observed at either delay. These transient FS-ESE results therefore corroborate that ^{5}TT is the predominant spin-active photoproduct in **FlePc2**, with relaxation into the triplet states manifold negligible under echo-detection conditions.

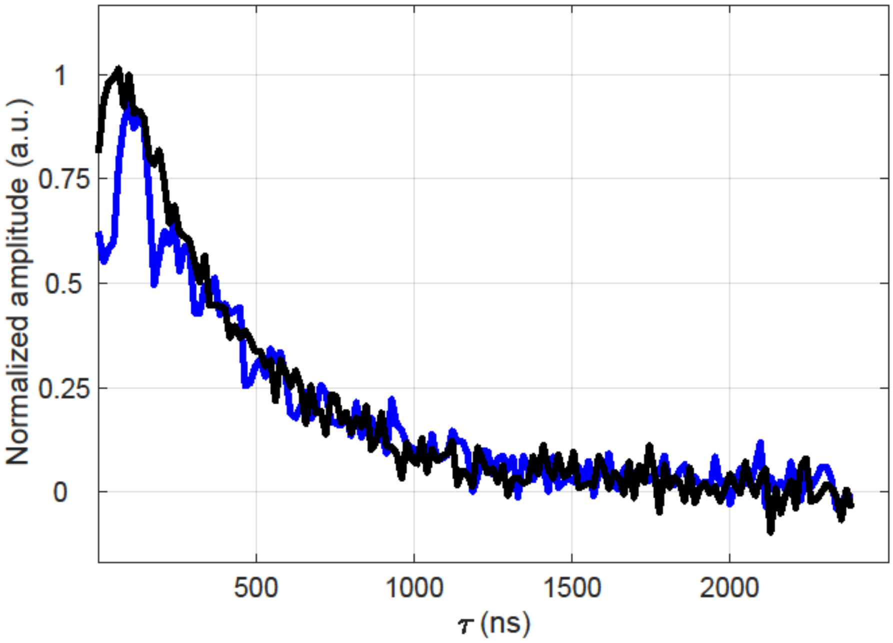

Figure S21. Measurements of phase-memory time T_m with the ESE experiment. Phase-memory decay was measured using a Carr–Purcell pulse sequence with two-step phase cycling ($\pi/2_x - \tau - [\pi - \tau -$ echo$]$ repeated). Echo intensities are plotted as a function of the interpulse delay τ. Measurements were performed at the two dominant resonance fields identified in the FS-ESE spectrum, 3280 G (blue) and 3509 G (black), at temperature 80 K. Analysis of the decay traces yielded nearly identical values of phase-memory time T_m, both within 481 ± 20 ns. It should be noted that the extracted T_m values represent the phase-memory (coherence) time of the ^{5}TT state rather than a thermalization constant; thus, while sublevel populations may undergo redistribution at 80 K, the identical decay behaviors observed at 3280 G and 3509 G reflect common coherence decay pathways of the same ^{5}TT species.

Discussion about the Rationale of Performing the Experiment at 80 K Rather than Commonly Used ≤ 20 K

While ≤20 K measurements are often used to capture the earliest spin-polarization patterns before thermal equilibration, our focus is the complementary thermally active regime around 80 K. At this temperature, k_BT (T = 80 K) far exceeds the Zeeman and ZFS level splittings (≲1 K combined), so phonon-assisted spin–lattice relaxation is energetically allowed. Despite this, the correlated ^{5}TT remains detectable with a finite $T_m \approx 0.48$ μs and exhibits a non-Boltzmann sublevel distribution, inconsistent with simple thermal equilibrium. Moreover, the echo signal weakens and vanishes by ≈130 K, and yet trEPR at 140 K (Figure S19) still reveals a ^{5}TT on the ~0.6 μs timescale — both trends confirming that relaxation channels are active, but the ^{5}TT population survives. Thus, our 80–140 K data demonstrate that the ^{5}TT is intrinsically robust in the presence of spin–lattice relaxation, a property directly relevant to functional operation, while the ≤20 K regime provides complementary mechanistic snapshots of the initial polarization.

Discussion about Non-Boltzmann Distribution of Sublevel States in the ^{5}TT State

At 80 K, the Zeeman and ZFS splittings are tiny compared to k_BT (for X-band, $g\mu_B B/k_B \approx 0.47$ K; D ~ 1.5 GHz ≈ 0.07 K), so thermal equilibrium would predict nearly flat sublevel populations ($\frac{p_{m+1}}{p_m} = \exp(-\Delta E/k_B T) \approx \exp(-0.47/80) \approx 0.994 \approx 1$). In contrast, our FS-ESE spectra can only be reproduced when a pronounced, non-flat sublevel distribution is used, ruling out a Boltzmann model. The physical origin of this non-Boltzmann distribution is the spin-selective formation dynamics of the quintet: photoexcitation produces a correlated ^{1}TT state, and anisotropic exchange/dipolar interactions mediate ^{1}TT ↔ ^{5}TT mixing that preferentially populates specific m_s sublevels depending on molecular orientation and field direction. Because inter-sublevel spin–lattice relaxation is slower than the multiexciton lifetime in our time-gated window at 80 K, these initially generated populations persist and are detected as a non-Boltzmann polarization. Moreover, the S = 2 nutation fingerprint and the nearly identical T_m values at distinct resonance fields (3280 G and 3509 G) indicate a single coherent ^{5}TT whose detected polarization is set by non-equilibrium formation dynamics within the time-gated window rather than by spin–lattice thermalization.

Section 4. Quantum Chemical Calculations

Table S4. Theoretically calculated bond lengths for the reported compounds. The asterisk (*) indicates the ground-state bond lengths of the monomeric **PhTIPSPc**, while the pound sign (#) corresponds to those of the fluorene bridge in its ground state.

Molecule	Label	Bond length at S_0(Å)	Bond length at S_1S_0 (Å)
Fluorene	B1	1.391#	1.438
	B2	1.392#	1.372
	B3	1.395#	1.419
	B4	1.395#	1.408
	B5	1.387#	1.373
	B6	1.511#	1.514
**PhTIPSPc**	B1	1.442*	1.418
	B2	1.442*	1.427
	B3	1.441*	1.446
	B4	1.440*	1.446
	B5	1.443*	1.428
	B6	1.434*	1.410
**FlePc2**	B1	1.434	1.434*
	B2	1.443	1.443*
	B3	1.442	1.441*
	B4	1.440	1.440*
	B5	1.440	1.440*
	B6	1.442	1.443*
	B7	1.391	1.391#
	B8	1.392	1.392#
	B9	1.395	1.395#
	B10	1.394	1.394#
	B11	1.387	1.386#
	B12	1.532	1.531#
	B13	1.442	1.417
	B14	1.440	1.425

	B15	1.440	1.445
	B16	1.442	1.447
	B17	1.443	1.428
	B18	1.434	1.410
**FlePhPc2**	B1	1.434	1.434*
	B2	1.443	1.443*
	B3	1.441	1.441*
	B4	1.440	1.440*
	B5	1.442	1.442*
	B6	1.442	1.442*
	B7	1.391	1.391#
	B8	1.392	1.392#
	B9	1.395	1.395#
	B10	1.394	1.394#
	B11	1.386	1.386#
	B12	1.531	1.531#
	B13	1.442	1.418
	B14	1.442	1.427
	B15	1.440	1.446
	B16	1.441	1.446
	B17	1.443	1.428
	B18	1.434	1.410

Table S5. Theoretical calculation data of the energy level for the reported compounds at their S_1S_0 geometry.

Molecule	States	RAS-2SF(4,4)/ eV	RAS-2SF(4,5)/ eV	RAS-2SF(6,5)/ eV	RAS-2SF(6,6)/ eV	RAS-2SF(6,7)/ eV
	^{1}[TT]	1.81	1.81	1.81	1.81	1.80
**FlePc2**	S_1S_0	3.23	3.20	3.15	3.12	3.13
	S_0S_1	3.47	3.48	3.48	3.49	3.46
	^{1}[TT]	1.80	1.80	1.79	1.79	1.78
**FlePhPc2**	S_1S_0	3.22	3.17	3.16	3.12	3.13
	S_0S_1	3.45	3.46	3.43	3.43	3.38

Table S6. Parameter values for evaluating FCWD for singlet fission and triplet-triplet annihilation (TTA). Details of the calculations are provided in reference.[13, 14]

Molecule	Parameters	$S_1S_0 \rightarrow S_0$	Singlet fission			Triplet-triplet annihilation		
			$S_0 \rightarrow T_1$	$S_1S_0 \rightarrow T_1$	FCWD/ ev^{-1}	$T_1 \rightarrow S_0$	$T_1 \rightarrow S_1S_0$	FCWD/ ev^{-1}
**FlePc2**	E_0/ cm^{-1}	15384	6936[a]	8448[b]	2.28	6936[a]	8448[b]	0.0096
	ħω/ cm^{-1}	1340	1340	1340		1340	1340	
	fwhm/ cm^{-1}	800	800	800		800	800	
	λ/ cm^{-1} [c]	718.0	1267.6	549.6		1354.6	137.6	
	S (calculate)	0.536	0.946	0.410		1.011	0.103	
**FlePhPc2**	E_0/ cm^{-1}	15174	6936[a]	8238[b]	2.41	6936[a]	8238[b]	0.0137
	ħω/ cm^{-1}	1357	1357	1357		1357	1357	
	fwhm/ cm^{-1}	800	800	800		800	800	
	λ/ cm^{-1} [c]	700.2	1252.0	551.8		1338.6	139.3	
	S (calculate)	0.516	0.923	0.407		0.986	0.103	

a. Experimental values from reference [24].

b. Difference of the E_0 values in S_1S_0 and T_1.

c. Calculated reorganization energies with TDDFT-TDA with *ω*B97x-D functional and 6-31G(d,p) basis set.

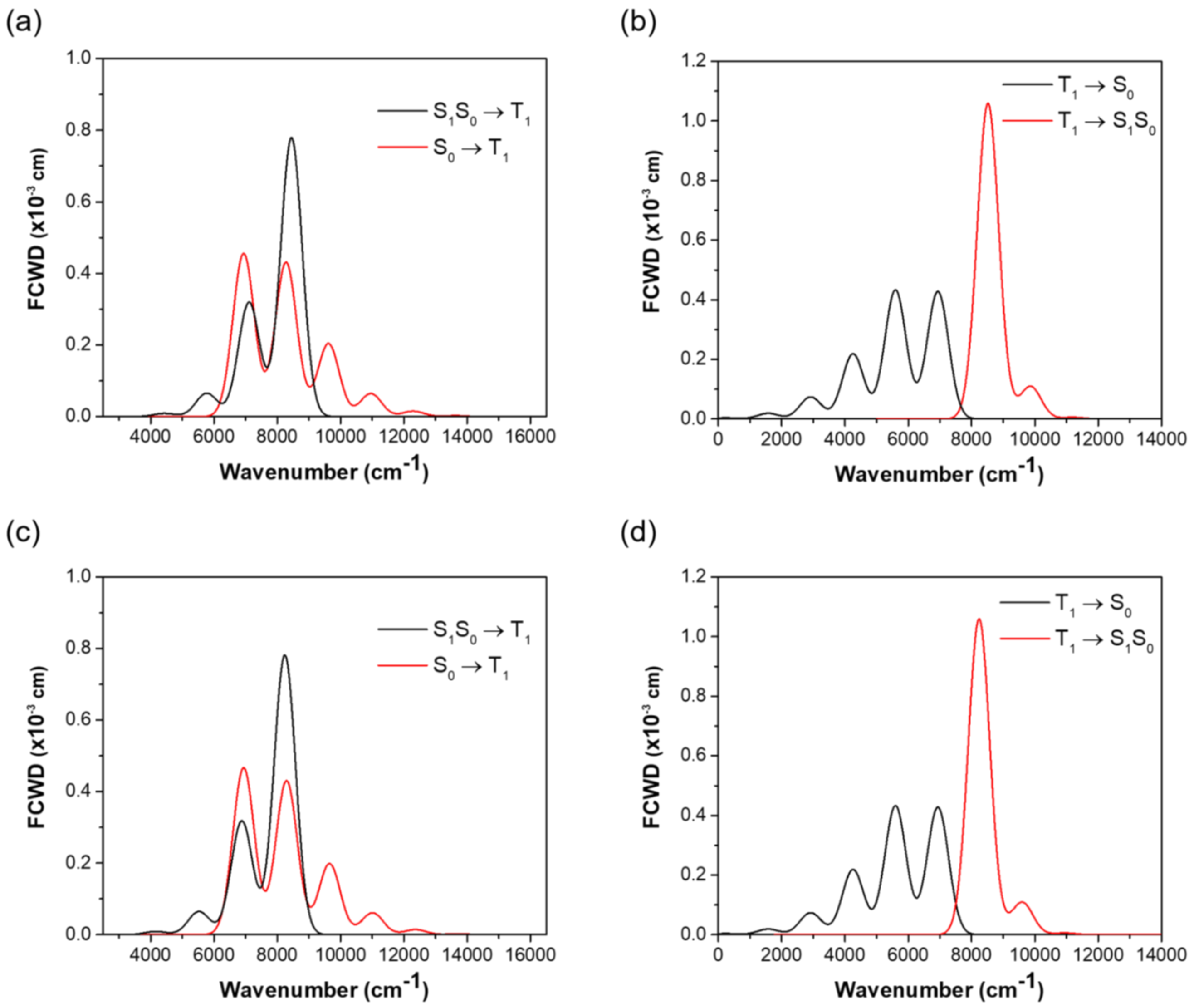

Figure S22. The FCWD functions shown for (a) and (b) **FlePc2**, and (c) and (d) **FlePhPc2**. Panels (a) and (c) correspond to $S_1S_0 \rightarrow T_1$/ $S_0 \rightarrow T_1$ transitions, representing SF between two pentacene chromophores. Panels (b) and (d) depict $T_1 \rightarrow S_0$/ $T_1 \rightarrow S_1S_0$ transitions associated with TTA. Details of the calculations are provided in reference [13].

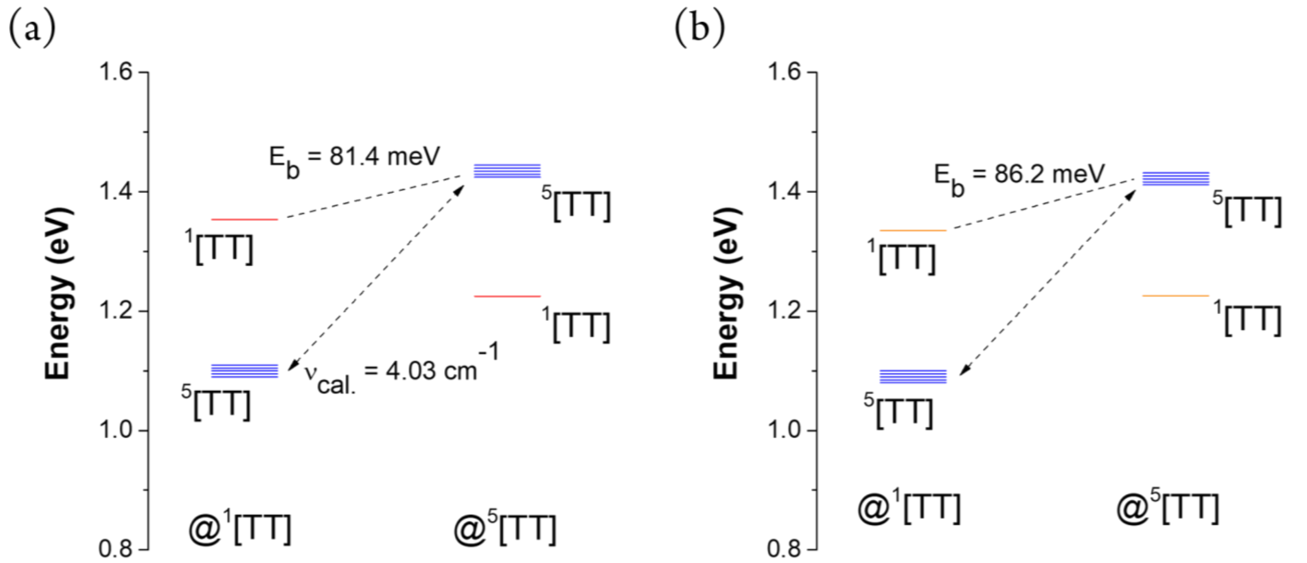

Figure S23. Energy levels for (a) **FlePc2** and (b) **FlePhPc2** of the multiexcitonic states based on the optimized ^{1}TT and ^{5}TT geometries. The observed energy difference is attributed to low-frequency torsional motions in the pentacenes and fluorene bridges.

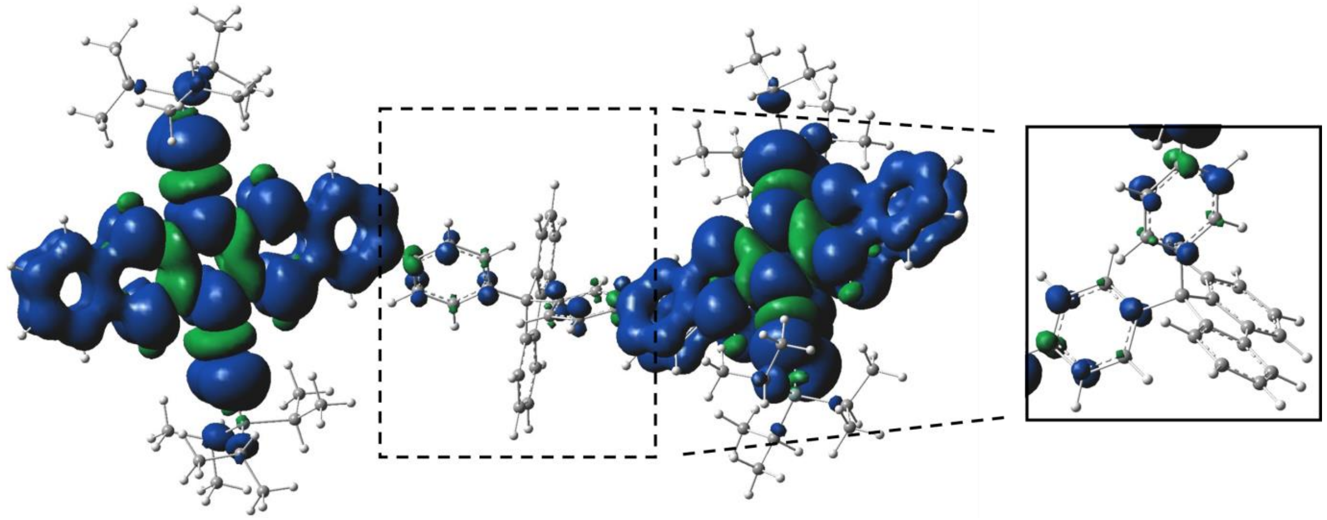

Figure S24. Spin density distribution of the **FlePhPc2** of ^{5}TT and its enlarged view, obtained by $\omega B97$x-D/6-31G(d,p). Isovalue for spin density is 0.0004.

Table S7. The optimized S_0S_0 structure of **FlePc2** based on ωB97X-D/6-31G** level of theory.

Si	-6.84138800	-4.25672500	3.29254300
Si	-6.82637100	5.15511700	-3.72179400
C	0.34345700	-2.59281200	-2.51511500
H	0.39388900	-1.56729600	-2.86618000
C	0.46335200	-3.65032700	-3.41632400
H	0.60129700	-3.44182800	-4.47250800
C	0.40847900	-4.97229900	-2.97381400
H	0.50701300	-5.78320700	-3.68823600
C	0.22544500	-5.25783500	-1.62334400
H	0.17983500	-6.28663500	-1.27929800
C	0.10132200	-4.20159800	-0.72661700
C	0.16439100	-2.87529300	-1.16926600
C	-0.00001000	-1.89972800	0.00022600
C	-1.24499700	-1.04349300	-0.24343300
C	-2.45764600	-1.38324600	0.26581800
H	-2.56633300	-2.24691400	0.91439400
C	-3.63655800	-0.62916100	-0.04351700
C	-4.86384900	-0.98827400	0.47121700
H	-4.93438500	-1.84965800	1.12363200
C	-6.03707500	-0.26969100	0.16846200
C	-7.29165300	-0.65681800	0.69594100
C	-8.46131700	0.07129400	0.38634800
C	-9.72109400	-0.29804800	0.90304600
H	-9.78062700	-1.16550500	1.55419900
C	-10.86666000	0.41427500	0.60123200
C	-12.15448900	0.04590400	1.12132600
H	-12.21937700	-0.82285100	1.76999100
C	-13.26280200	0.76581200	0.80953500

H	-14.23036900	0.47803600	1.20803000
C	-13.17265900	1.91187400	-0.04729900
H	-14.07365300	2.46966600	-0.28148400
C	-11.97694000	2.29770400	-0.56215200
H	-11.90315600	3.16431000	-1.21311800
C	-10.77627700	1.56791600	-0.26159200
C	-9.54790100	1.94008900	-0.77467500
H	-9.47756200	2.81211200	-1.41770400
C	-8.37104200	1.21983000	-0.48016600
C	-7.11594800	1.59880700	-1.01460800
C	-5.94280300	0.87512100	-0.69935400
C	-4.67619300	1.23870500	-1.21247500
H	-4.60636000	2.10922300	-1.85760400
C	-3.53709800	0.51643400	-0.90965100
C	-2.23756500	0.85364900	-1.42201800
H	-2.14396000	1.71996000	-2.07074500
C	-1.14780400	0.10945500	-1.10493000
H	-0.17231600	0.39014100	-1.49094300
C	-7.33295100	-1.79908100	1.54648600
C	-7.24066600	-2.78333500	2.25856300
C	-4.94640200	-4.41633100	3.29992600
H	-4.71425000	-5.25849900	3.96900400
C	-4.28475000	-3.15931200	3.88585700
H	-4.53686100	-2.26652700	3.30289300
H	-3.19340400	-3.25885600	3.87836800
H	-4.59855200	-2.97176100	4.91821200
C	-4.36345200	-4.74895600	1.91825500
H	-4.69791600	-5.72710500	1.55842700
H	-3.26694700	-4.76721800	1.95322200

H	-4.65734100	-4.00882100	1.16534100
C	-7.62949800	-5.76813900	2.45830600
H	-7.02854000	-5.90969800	1.54838300
C	-9.08086400	-5.54999200	2.00831300
H	-9.43924200	-6.41068000	1.43128400
H	-9.17719200	-4.65980500	1.37968400
H	-9.75430000	-5.42878300	2.86280400
C	-7.49522700	-7.03978000	3.30852100
H	-8.11993800	-6.98074800	4.20695400
H	-6.46349100	-7.21657800	3.63220100
H	-7.82085300	-7.92262000	2.74623700
C	-7.44927100	-3.89401300	5.05503500
H	-7.10139500	-2.86896100	5.24821000
C	-8.97946400	-3.89009300	5.17111800
H	-9.44604400	-3.25641500	4.40971000
H	-9.29572900	-3.52011900	6.15340500
H	-9.38601800	-4.90154400	5.06024300
C	-6.81925800	-4.80504300	6.11924000
H	-7.10268800	-5.85285900	5.97419300
H	-7.15365300	-4.51474600	7.12222000
H	-5.72589900	-4.75434900	6.11037700
C	-7.03378100	2.71846900	-1.88911500
C	-6.96017500	3.66794400	-2.64816900
C	-5.40336100	4.86939000	-4.95643700
H	-5.86128100	4.31627200	-5.78926900
C	-4.27012500	3.99378100	-4.40045400
H	-3.78981500	4.45500900	-3.53044900
H	-3.49362500	3.83968000	-5.15920500
H	-4.63966000	3.01130100	-4.09291900

C	-4.85312900	6.18709400	-5.52460900
H	-5.63321500	6.79770300	-5.99038300
H	-4.09106900	5.99245400	-6.28809200
H	-4.38296800	6.79478000	-4.74382400
C	-6.48789000	6.62338200	-2.56604400
H	-6.40674900	7.51353400	-3.20697100
C	-5.16338100	6.45590600	-1.80651400
H	-5.16742900	5.53703100	-1.20922000
H	-5.00150800	7.29512400	-1.11982700
H	-4.30172500	6.41229800	-2.47941800
C	-7.64137900	6.84710900	-1.57681900
H	-8.58141300	7.08684500	-2.08279200
H	-7.41365100	7.67655500	-0.89697100
H	-7.81061100	5.95480100	-0.96345100
C	-8.46548500	5.35010500	-4.67320800
H	-8.35444200	4.70633900	-5.55779100
C	-8.68179700	6.78730500	-5.17277700
H	-8.79805800	7.48837800	-4.33910100
H	-9.59043700	6.85356600	-5.78233100
H	-7.84953100	7.14404400	-5.78790800
C	-9.69222800	4.84605800	-3.89792500
H	-9.58959300	3.78985700	-3.63264000
H	-10.60165200	4.95551700	-4.50076700
H	-9.84548800	5.40786800	-2.96996800
Si	6.84082400	-4.25673000	-3.29285100
Si	6.82691500	5.15472700	3.72197300
C	-0.34346900	-2.59261800	2.51562000
H	-0.39386200	-1.56707400	2.86660900
C	-0.46337100	-3.65006300	3.41690900

H	-0.60128800	-3.44148300	4.47308100
C	-0.40853900	-4.97206900	2.97449600
H	-0.50707800	-5.78292200	3.68898000
C	-0.22553400	-5.25771000	1.62404400
H	-0.17994800	-6.28653700	1.28007500
C	-0.10139900	-4.20154200	0.72723700
C	-0.16443500	-2.87520300	1.16978800
C	1.24501200	-1.04352800	0.24381400
C	2.45760600	-1.38330000	-0.26555200
H	2.56620800	-2.24694900	-0.91416800
C	3.63656500	-0.62926000	0.04370200
C	4.86379700	-0.98838800	-0.47116100
H	4.93424500	-1.84974600	-1.12361900
C	6.03707200	-0.26985400	-0.16848500
C	7.29158600	-0.65700100	-0.69609800
C	8.46130600	0.07104800	-0.38657000
C	9.72102200	-0.29831600	-0.90340100
H	9.78046400	-1.16574100	-1.55460500
C	10.86664200	0.41394800	-0.60165300
C	12.15441100	0.04555600	-1.12188100
H	12.21920800	-0.82316700	-1.77059700
C	13.26277900	0.76540600	-0.81015300
H	14.23030000	0.47761500	-1.20875000
C	13.17275600	1.91142700	0.04674900
H	14.07379200	2.46917300	0.28088200
C	11.97709800	2.29727700	0.56172900
H	11.90340500	3.16385100	1.21274500
C	10.77638100	1.56754900	0.26123900
C	9.54806400	1.93974400	0.77444900

H	9.47781500	2.81173800	1.41752800
C	8.37115100	1.21954400	0.48000900
C	7.11611900	1.59854500	1.01458000
C	5.94291800	0.87492000	0.69939400
C	4.67636400	1.23852500	1.21264300
H	4.60661800	2.10901900	1.85781400
C	3.53721800	0.51630400	0.90989000
C	2.23773900	0.85354000	1.42238300
H	2.14421800	1.71982900	2.07115300
C	1.14792600	0.10939400	1.10536100
H	0.17248100	0.39009200	1.49147100
C	7.33275400	-1.79922100	-1.54670800
C	7.24032500	-2.78342500	-2.25883500
C	4.94581700	-4.41611400	-3.30016100
H	4.71354200	-5.25824300	-3.96924500
C	4.28428700	-3.15900900	-3.88604900
H	4.53652600	-2.26626000	-3.30308500
H	3.19292900	-3.25842600	-3.87851700
H	4.59806800	-2.97148300	-4.91841500
C	4.36287900	-4.74869400	-1.91847600
H	4.69724600	-5.72688600	-1.55867500
H	3.26636900	-4.76683400	-1.95340500
H	4.65687500	-4.00860400	-1.16556000
C	7.62879200	-5.76826500	-2.45870000
H	7.02786500	-5.90977300	-1.54874800
C	9.08020900	-5.55031300	-2.00878000
H	9.43850900	-6.41105900	-1.43179100
H	9.17668300	-4.66015200	-1.38013500
H	9.75361400	-5.42916900	-2.86330400

C	7.49431400	-7.03986600	-3.30894300
H	8.11898100	-6.98088500	-4.20741000
H	6.46253700	-7.21652500	-3.63256900
H	7.81986200	-7.92276200	-2.74670300
C	7.44867000	-3.89403200	-5.05536000
H	7.10091100	-2.86893100	-5.24848400
C	8.97885800	-3.89029500	-5.17151600
H	9.44555100	-3.25670100	-4.41010700
H	9.29512100	-3.52032300	-6.15380400
H	9.38529400	-4.90179900	-5.06069800
C	6.81849600	-4.80495000	-6.11956500
H	7.10180200	-5.85280600	-5.97456400
H	7.15288300	-4.51466400	-7.12255100
H	5.72514300	-4.75412100	-6.11065200
C	7.03407300	2.71816700	1.88914900
C	6.96056900	3.66760800	2.64825700
C	5.40404100	4.86895800	4.95676100
H	5.86203900	4.31575100	5.78949100
C	4.27070100	3.99344200	4.40084300
H	3.79030000	4.45476300	3.53093800
H	3.49428900	3.83930800	5.15967800
H	4.64015900	3.01097300	4.09318000
C	4.85393700	6.18663900	5.52510900
H	5.63410400	6.79716800	5.99085100
H	4.09195000	5.99197000	6.28865800
H	4.38372000	6.79441700	4.74442800
C	6.48833900	6.62306000	2.56633700
H	6.40732400	7.51318600	3.20731500
C	5.16372300	6.45568000	1.80697400

H	5.16764500	5.53682800	1.20964500
H	5.00180300	7.29493200	1.12033800
H	4.30215200	6.41209300	2.47998900
C	7.64170900	6.84678000	1.57697000
H	8.58182300	7.08643500	2.08283000
H	7.41393500	7.67627600	0.89719900
H	7.81080900	5.95449600	0.96353000
C	8.46614000	5.34964400	4.67321300
H	8.35517200	4.70586900	5.55779900
C	8.68256000	6.78682800	5.17277900
H	8.79877100	7.48790700	4.33910100
H	9.59126200	6.85304300	5.78224700
H	7.85036800	7.14359700	5.78799400
C	9.69277900	4.84556600	3.89778600
H	9.59008200	3.78937000	3.63250300
H	10.60227600	4.95499300	4.50052500
H	9.84595000	5.40738000	2.96981600

Table S8. The optimized S_1S_0 structure of **FlePc2** based on ωB97X-D/6-31G** level of theory.

Si	6.87483800	-4.33761800	-3.13729700
Si	6.86592200	5.28161700	3.56143900
C	-0.27769800	-2.55600700	2.38795500
H	-0.32641100	-1.53929200	2.76492000
C	-0.36965500	-3.63869200	3.26181600
H	-0.48781300	-3.46115900	4.32601000
C	-0.30937900	-4.94759700	2.78155000
H	-0.38476400	-5.77870400	3.47527800
C	-0.14773400	-5.19475300	1.42081900
H	-0.09467100	-6.21335900	1.04869700
C	-0.05237100	-4.11313000	0.55097900
C	-0.12385900	-2.80120100	1.03206100
C	0.00703800	-1.78953300	-0.11008000
C	1.23952200	-0.92042200	0.14970600
C	2.48002100	-1.29514900	-0.32054100
H	2.58792200	-2.17979900	-0.94080100
C	3.63689900	-0.55367100	-0.00609400
C	4.90364200	-0.94428400	-0.48301500
H	4.97299500	-1.82923300	-1.10390700
C	6.06042200	-0.24202600	-0.18116800
C	7.33680900	-0.66428100	-0.66975900
C	8.52459800	0.06271100	-0.36093600
C	9.76939900	-0.33537400	-0.83198400
H	9.83424900	-1.22692300	-1.44991300
C	10.95065100	0.38012500	-0.53489500
C	12.21894000	-0.02414400	-1.01243400
H	12.28699100	-0.91637200	-1.62826700
C	13.34943900	0.69872600	-0.70362200

H	14.31687900	0.37803000	-1.07602500
C	13.25768900	1.85644800	0.09619400
H	14.15515900	2.41823100	0.33373300
C	12.03634400	2.27451400	0.57546000
H	11.96122500	3.16503100	1.19334500
C	10.85818800	1.55281100	0.27531200
C	9.59052400	1.95457200	0.75312500
H	9.52129700	2.85056900	1.36317700
C	8.43107700	1.24638500	0.46601500
C	7.15429500	1.65725300	0.96636000
C	5.96245400	0.93699700	0.64880800
C	4.71037200	1.33167700	1.11297900
H	4.63645800	2.22422200	1.72759500
C	3.53435100	0.60920300	0.81103400
C	2.25377200	0.98044100	1.28239000
H	2.15725000	1.86525200	1.90541700
C	1.14064100	0.23602400	0.96274900
H	0.16590700	0.54401900	1.32903800
C	7.37705400	-1.82457600	-1.47313600
C	7.28079100	-2.83521000	-2.15389600
C	4.97696900	-4.45405800	-3.19765700
H	4.74551300	-5.31240400	-3.84614300
C	4.36231800	-3.20283500	-3.84390800
H	4.61557700	-2.29761000	-3.28084600
H	3.26916300	-3.27880600	-3.87080800
H	4.71471700	-3.05460400	-4.87024300
C	4.34532400	-4.72871000	-1.82478800
H	4.64137300	-5.70446800	-1.42681300
H	3.25013300	-4.71777100	-1.89191500

H	4.63836800	-3.97510100	-1.08491900
C	7.59611300	-5.83456100	-2.21815300
H	6.96449300	-5.92078300	-1.32223300
C	9.03867700	-5.63657900	-1.73246100
H	9.35472300	-6.48028000	-1.10750700
H	9.14075600	-4.72210000	-1.14062200
H	9.74098900	-5.57171800	-2.56964600
C	7.45139800	-7.13701400	-3.01841100
H	8.10338400	-7.13280700	-3.89921300
H	6.42498400	-7.29989800	-3.36551400
H	7.73583900	-8.00365200	-2.41029700
C	7.54458000	-4.07107600	-4.89574700
H	7.23422900	-3.04589800	-5.14381800
C	9.07683400	-4.11772600	-4.96491100
H	9.54012700	-3.46576400	-4.21701500
H	9.43323800	-3.80015900	-5.95185500
H	9.44856000	-5.13506400	-4.79881300
C	6.91788300	-5.00947000	-5.93791600
H	7.16146400	-6.05809800	-5.73707000
H	7.29260900	-4.77612900	-6.94157800
H	5.82711400	-4.92333200	-5.96674200
C	7.07206500	2.79160500	1.80010700
C	6.99776500	3.76636300	2.53298400
C	5.42501600	5.04438300	4.78621300
H	5.86682900	4.50402900	5.63596800
C	4.28676800	4.17097400	4.23728300
H	3.82009800	4.62017700	3.35362800
H	3.50132200	4.04101100	4.99143100
H	4.64828100	3.17848500	3.95292900

C	4.88600100	6.38125700	5.31903000
H	5.66899500	6.99152900	5.78033200
H	4.11288600	6.21353000	6.07787500
H	4.43270200	6.97788800	4.51993700
C	6.55705600	6.72654500	2.36653700
H	6.48118600	7.63287800	2.98519300
C	5.23855700	6.55734400	1.59733700
H	5.23648300	5.62287100	1.02465200
H	5.09550900	7.38025800	0.88701800
H	4.36928300	6.54324700	2.26166100
C	7.72337400	6.91053500	1.38438600
H	8.66166100	7.14908400	1.89420300
H	7.51449400	7.72642600	0.68233800
H	7.88581200	6.00098600	0.79493400
C	8.49507000	5.48914100	4.52872600
H	8.36502100	4.87378100	5.43077000
C	8.72214000	6.93817600	4.98748300
H	8.85907200	7.61206800	4.13477100
H	9.62276500	7.01215800	5.60806900
H	7.88568200	7.32366800	5.57910900
C	9.72560100	4.94792700	3.78527800
H	9.61302400	3.88547700	3.55046600
H	10.62869300	5.06485000	4.39632100
H	9.89715500	5.47986800	2.84292500
Si	-6.72220400	-4.34641600	3.27388000
Si	-7.05552000	5.15162400	-3.61675500
C	0.35509900	-2.40540500	-2.64574000
H	0.39493300	-1.36980800	-2.96710800
C	0.48718300	-3.43472700	-3.57738900

H	0.62497200	-3.19400300	-4.62665000
C	0.44633900	-4.76950000	-3.17374900
H	0.55408900	-5.55801800	-3.91149000
C	0.26814000	-5.09657200	-1.83197100
H	0.23643900	-6.13541900	-1.51786300
C	0.13435400	-4.06852800	-0.90444100
C	0.17932100	-2.72890400	-1.30886500
C	-1.26055600	-0.95733700	-0.31796800
C	-2.45504800	-1.32867900	0.21179000
H	-2.53190700	-2.20068400	0.85361800
C	-3.65703300	-0.59904300	-0.06729000
C	-4.86551800	-0.99176900	0.46693900
H	-4.90347200	-1.86086700	1.11177300
C	-6.06088800	-0.29803500	0.19343400
C	-7.29618400	-0.72016700	0.73915700
C	-8.48792400	-0.01589100	0.45967700
C	-9.72898600	-0.42001700	0.99517800
H	-9.75649200	-1.29592000	1.63707900
C	-10.89597600	0.26921500	0.72306400
C	-12.16503600	-0.13459400	1.26256900
H	-12.19791300	-1.01197500	1.90195600
C	-13.29513000	0.56318000	0.97995500
H	-14.24822500	0.24868000	1.39285300
C	-13.24725400	1.72072700	0.13527200
H	-14.16496500	2.26031200	-0.07549900
C	-12.07053100	2.13981000	-0.39708500
H	-12.02872900	3.01526100	-1.03898100
C	-10.84816400	1.43439000	-0.12754300
C	-9.63842400	1.84044200	-0.65894600

H	-9.59965500	2.72103700	-1.29291000
C	-8.44028400	1.14399300	-0.39486600
C	-7.20453800	1.55735800	-0.94863400
C	-6.00954900	0.85749800	-0.66366400
C	-4.76184900	1.25518000	-1.19758300
H	-4.72510800	2.13306400	-1.83552300
C	-3.60102200	0.55640300	-0.92414700
C	-2.31988800	0.92727700	-1.45919700
H	-2.25882700	1.80087100	-2.10199700
C	-1.20760600	0.20519600	-1.17119000
H	-0.24727800	0.51287000	-1.57366400
C	-7.29647500	-1.87353900	1.57555400
C	-7.17135800	-2.86506800	2.27232900
C	-4.82461200	-4.46617300	3.23854600
H	-4.55983200	-5.31510600	3.88656300
C	-4.17735200	-3.20576700	3.83325900
H	-4.46182300	-2.30823700	3.27292300
H	-3.08459600	-3.28100400	3.79976100
H	-4.47172800	-3.04396900	4.87560900
C	-4.26572800	-4.76056200	1.83833400
H	-4.58599100	-5.73960300	1.46818200
H	-3.16864300	-4.75425500	1.84840700
H	-4.59342900	-4.01411700	1.10590300
C	-7.49736500	-5.85958700	2.43061600
H	-6.91273000	-5.97434600	1.50642200
C	-8.96176000	-5.66332000	2.01392000
H	-9.31480700	-6.52144200	1.42983300
H	-9.08869600	-4.76503200	1.40247300
H	-9.61972700	-5.56984900	2.88381100

C	-7.32061700	-7.14188400	3.25672100
H	-7.92750200	-7.10979300	4.16862800
H	-6.27911900	-7.30336400	3.55623000
H	-7.64058900	-8.02181200	2.68668500
C	-7.30058500	-4.02600300	5.05437700
H	-6.97011600	-2.99728300	5.25828500
C	-8.82780700	-4.05659400	5.20174900
H	-9.32341700	-3.42003300	4.46137600
H	-9.13120400	-3.71042200	6.19669200
H	-9.21518500	-5.07448600	5.08137300
C	-6.63003600	-4.94163000	6.08949900
H	-6.89543900	-5.99240500	5.93232800
H	-6.94899400	-4.67508700	7.10402200
H	-5.53835200	-4.86862900	6.05885600
C	-7.16493600	2.68815100	-1.81167900
C	-7.12916600	3.64771000	-2.56066200
C	-5.65621500	4.91616200	-4.88835900
H	-6.12031500	4.36213300	-5.71715500
C	-4.48908600	4.06200100	-4.37033900
H	-3.99884900	4.52498200	-3.50682900
H	-3.72788000	3.93524300	-5.14938100
H	-4.82736100	3.06745900	-4.06548500
C	-5.15201900	6.25392700	-5.45230000
H	-5.95755700	6.85001100	-5.89285400
H	-4.40289000	6.08765600	-6.23504000
H	-4.67963400	6.86401300	-4.67471500
C	-6.72433400	6.61217700	-2.44921600
H	-6.68002000	7.51239000	-3.07962500
C	-5.37838800	6.46697400	-1.72359600

H	-5.34615800	5.54051600	-1.13900200
H	-5.22024600	7.30078600	-1.02949300
H	-4.53231700	6.45309300	-2.41724000
C	-7.85888600	6.79459500	-1.43004000
H	-8.81638200	7.01749500	-1.91038500
H	-7.63525400	7.62058700	-0.74465500
H	-7.99148900	5.89051700	-0.82495900
C	-8.72100900	5.32082500	-4.52650900
H	-8.61557400	4.69355900	-5.42354900
C	-8.98369600	6.75979400	-4.99795500
H	-9.09741500	7.44479700	-4.15069200
H	-9.90766300	6.81306800	-5.58532800
H	-8.17494600	7.14644800	-5.62620500
C	-9.91659800	4.77528500	-3.73087600
H	-9.78263300	3.71784000	-3.48521100
H	-10.84248900	4.87215300	-4.31034600
H	-10.06097000	5.31871200	-2.79060300

Table S9. The optimized [1]TT structure of **FlePc2** based on ωB97X-D/6-31G** level of theory.

Si	-6.80055000	4.29629200	-3.32260300
Si	-6.91868000	-5.17673300	3.60988600
C	0.31249800	2.55662000	2.51851000
H	0.35717700	1.53059400	2.86909400
C	0.42219700	3.61372300	3.42143400
H	0.54752800	3.40497200	4.47913300
C	0.37294600	4.93589100	2.97862300
H	0.46312800	5.74652500	3.69446300
C	0.20591800	5.22210000	1.62619200
H	0.16438600	6.25107200	1.28213500
C	0.09257700	4.16619100	0.72758400
C	0.15010700	2.83986200	1.17084400
C	0.00000600	1.86396200	-0.00027600
C	-1.24910100	1.00956200	0.22975600
C	-2.46021200	1.36215900	-0.28619700
H	-2.55747600	2.23130900	-0.92931000
C	-3.64081000	0.61554400	0.00928900
C	-4.87019000	0.98813000	-0.51331800
H	-4.92835000	1.85552200	-1.15905900
C	-6.04517300	0.27816200	-0.22552900
C	-7.29853100	0.68067800	-0.76165200
C	-8.48079500	-0.04445600	-0.46645700
C	-9.72791300	0.33765300	-0.98860900
H	-9.77710800	1.21113100	-1.63260200
C	-10.88952500	-0.37189200	-0.70241600
C	-12.16417000	0.01093500	-1.22896000
H	-12.21785700	0.88577600	-1.87049000
C	-13.28474000	-0.70496700	-0.93250400

H	-14.24598500	-0.40506000	-1.33727400
C	-13.20971900	-1.85429400	-0.08857800
H	-14.11508400	-2.40937500	0.13490800
C	-12.01594600	-2.25265200	0.43309700
H	-11.95333800	-3.12543100	1.07701500
C	-10.81434100	-1.52945900	0.14775100
C	-9.58376600	-1.91458000	0.66916200
H	-9.52540700	-2.79271800	1.30511600
C	-8.40548400	-1.20175800	0.39104400
C	-7.15205400	-1.59554200	0.93490900
C	-5.96581100	-0.87499100	0.63368400
C	-4.71205500	-1.25198100	1.15201700
H	-4.65317700	-2.12796000	1.79097500
C	-3.55649300	-0.53332700	0.86390200
C	-2.26976100	-0.88430900	1.38391900
H	-2.18759800	-1.75529800	2.02797800
C	-1.16747900	-0.14467900	1.08097200
H	-0.19845000	-0.43597800	1.47523000
C	-7.32449700	1.82733700	-1.60065400
C	-7.21937000	2.81766400	-2.30416700
C	-4.90439900	4.44124100	-3.31279400
H	-4.65992400	5.28748400	-3.97224700
C	-4.24759600	3.18426700	-3.90426200
H	-4.51174700	2.28839800	-3.33143600
H	-3.15559700	3.27504300	-3.88666700
H	-4.55403200	3.00829200	-4.94085300
C	-4.33072000	4.75687700	-1.92328000
H	-4.65995600	5.73468400	-1.55774900
H	-3.23384900	4.76604800	-1.94869800

H	-4.63733000	4.01275300	-1.17942900
C	-7.58422800	5.80691100	-2.48263700
H	-6.99033400	5.93613900	-1.56626900
C	-9.04121500	5.59650300	-2.04735000
H	-9.39788400	6.45504500	-1.46608200
H	-9.15022100	4.70175900	-1.42730700
H	-9.70797700	5.48802500	-2.90875900
C	-7.43248900	7.08454100	-3.32086100
H	-8.04962800	7.03797400	-4.22523600
H	-6.39657600	7.25592000	-3.63392700
H	-7.75620200	7.96516400	-2.75401300
C	-7.39647600	3.95264500	-5.09302700
H	-7.05433700	2.92673800	-5.29185000
C	-8.92564200	3.96073500	-5.22176100
H	-9.40311700	3.32436700	-4.46940600
H	-9.23635600	3.60098600	-6.20960500
H	-9.32582700	4.97418600	-5.10607800
C	-6.75119700	4.86781500	-6.14446000
H	-7.02839100	5.91640300	-5.99311000
H	-7.07934600	4.58814400	-7.15250900
H	-5.65833600	4.80923600	-6.12704000
C	-7.08571100	-2.71987000	1.79876500
C	-7.02691300	-3.67718300	2.55078700
C	-5.50771200	-4.91707900	4.86377500
H	-5.96962000	-4.36621300	5.69590200
C	-4.35863200	-4.04901000	4.32885600
H	-3.87221300	-4.50841800	3.46131100
H	-3.59016000	-3.90886600	5.09840500
H	-4.71412600	-3.06037000	4.02444400

C	-4.97863500	-6.24560400	5.42678600
H	-5.77066000	-6.85085900	5.87919000
H	-4.22263000	-6.06602100	6.19991400
H	-4.50716400	-6.85228500	4.64599500
C	-6.58063500	-6.63579700	2.44235800
H	-6.51727100	-7.53368300	3.07444800
C	-5.24487300	-6.47456800	1.70146300
H	-5.23126500	-5.54913100	1.11451900
H	-5.08351500	-7.30782200	1.00742700
H	-4.39141200	-6.44802800	2.38560700
C	-7.72384300	-6.83609400	1.43628900
H	-8.67301900	-7.07005800	1.92773800
H	-7.49693200	-7.66111900	0.75081600
H	-7.87497000	-5.93563400	0.83015800
C	-8.57057200	-5.36751500	4.54004700
H	-8.46317000	-4.73707000	5.43461800
C	-8.80757800	-6.80904300	5.01728200
H	-8.92277400	-7.49714400	4.17273400
H	-9.72320300	-6.87372300	5.61645800
H	-7.98573100	-7.18351000	5.63583400
C	-9.78314100	-4.84037600	3.75782100
H	-9.66713200	-3.78162200	3.50873700
H	-10.70062600	-4.94929900	4.34841700
H	-9.93090900	-5.38755900	2.82024800
Si	6.80039100	4.29647300	3.32221400
Si	6.91887000	-5.17721300	-3.60935700
C	-0.31250400	2.55599700	-2.51923000
H	-0.35718700	1.52988500	-2.86956200
C	-0.42222500	3.61287500	-3.42241300

H	-0.54757000	3.40386200	-4.48005900
C	-0.37298200	4.93515200	-2.97993000
H	-0.46317900	5.74560900	-3.69596900
C	-0.20594100	5.22169700	-1.62757100
H	-0.16441600	6.25075500	-1.28376800
C	-0.09258100	4.16601000	-0.72870400
C	-0.15010000	2.83957200	-1.17163600
C	1.24911400	1.00950800	-0.23011400
C	2.46020800	1.36215900	0.28584000
H	2.55746100	2.23139900	0.92883300
C	3.64080600	0.61547400	-0.00948300
C	4.87016700	0.98809900	0.51313900
H	4.92831400	1.85557200	1.15877300
C	6.04514900	0.27806200	0.22550800
C	7.29848600	0.68060000	0.76166500
C	8.48074600	-0.04460300	0.46662400
C	9.72784300	0.33752700	0.98881300
H	9.77702300	1.21107200	1.63271600
C	10.88945300	-0.37207900	0.70276400
C	12.16407600	0.01077000	1.22934500
H	12.21774900	0.88567600	1.87078800
C	13.28464600	-0.70519300	0.93303100
H	14.24587400	-0.40526900	1.33782700
C	13.20964600	-1.85460500	0.08921900
H	14.11500900	-2.40973200	-0.13415600
C	12.01589300	-2.25298600	-0.43248700
H	11.95330100	-3.12583000	-1.07631900
C	10.81428900	-1.52973200	-0.14728700
C	9.58373600	-1.91487500	-0.66873100

H	9.52539200	-2.79307700	-1.30459900
C	8.40545600	-1.20199400	-0.39075700
C	7.15204800	-1.59580300	-0.93465400
C	5.96580600	-0.87519100	-0.63357200
C	4.71206700	-1.25222100	-1.15191700
H	4.65320100	-2.12828200	-1.79076400
C	3.55650600	-0.53351000	-0.86394500
C	2.26978900	-0.88454500	-1.38396400
H	2.18763800	-1.75562500	-2.02790200
C	1.16750800	-0.14485600	-1.08116500
H	0.19848900	-0.43619800	-1.47541900
C	7.32443500	1.82734600	1.60054700
C	7.21928600	2.81774900	2.30394900
C	4.90424800	4.44149600	3.31212400
H	4.65971000	5.28780700	3.97146600
C	4.24731300	3.18459700	3.90360800
H	4.51152100	2.28866700	3.33090500
H	3.15532100	3.27540900	3.88583800
H	4.55358600	3.00870800	4.94026200
C	4.33078500	4.75702700	1.92249700
H	4.66011400	5.73478800	1.55692600
H	3.23391100	4.76624300	1.94775200
H	4.63747700	4.01282300	1.17875900
C	7.58424600	5.80699500	2.48223900
H	6.99050200	5.93615500	1.56576400
C	9.04129700	5.59650700	2.04720300
H	9.39808000	6.45498200	1.46590700
H	9.15037700	4.70169800	1.42726500
H	9.70791900	5.48809600	2.90872800

C	7.43240800	7.08470600	3.32032100
H	8.04940400	7.03820800	4.22479700
H	6.39644900	7.25614100	3.63320700
H	7.75623200	7.96526900	2.75344200
C	7.39605000	3.95292500	5.09274800
H	7.05381800	2.92705500	5.29159600
C	8.92519800	3.96092600	5.22170000
H	9.40274100	3.32448300	4.46945100
H	9.23574800	3.60121700	6.20961000
H	9.32546500	4.97434400	5.10601400
C	6.75068000	4.86821200	6.14402300
H	7.02796700	5.91677100	5.99263800
H	7.07866300	4.58859100	7.15213800
H	5.65781800	4.80970600	6.12644800
C	7.08573000	-2.72021100	-1.79840900
C	7.02698300	-3.67759100	-2.55034700
C	5.50805600	-4.91763800	-4.86343600
H	5.97007100	-4.36684000	-5.69554900
C	4.35892300	-4.04951100	-4.32872200
H	3.87239700	-4.50884300	-3.46119800
H	3.59054300	-3.90942200	-5.09837300
H	4.71439300	-3.06085100	-4.02435000
C	4.97902800	-6.24619700	-5.42641100
H	5.77109900	-6.85149800	-5.87867300
H	4.22311900	-6.06666100	-6.19964400
H	4.50745400	-6.85281200	-4.64563100
C	6.58067900	-6.63620100	-2.44177700
H	6.51738700	-7.53412800	-3.07381700
C	5.24483000	-6.47492100	-1.70105300

H	5.23115400	-5.54944600	-1.11416900
H	5.08338600	-7.30813000	-1.00698300
H	4.39145000	-6.44842100	-2.38530000
C	7.72376700	-6.83643900	-1.43555900
H	8.67300000	-7.07043600	-1.92687900
H	7.49677100	-7.66142000	-0.75006100
H	7.87482300	-5.93594200	-0.82946500
C	8.57087500	-5.36805800	-4.53930200
H	8.46359100	-4.73765300	-5.43391600
C	8.80792200	-6.80961100	-5.01644200
H	8.92300600	-7.49767500	-4.17184900
H	9.72362100	-6.87432900	-5.61550200
H	7.98614800	-7.18409600	-5.63508000
C	9.78335200	-4.84090000	-3.75694900
H	9.66732100	-3.78213500	-3.50791600
H	10.70090800	-4.94985000	-4.34742900
H	9.93100400	-5.38805000	-2.81933800

Table S10. The optimized ^{5}TT structure of **FlePc2** based on ωB97X-D/6-31G** level of theory.

Si	-6.78143800	-4.40168500	3.14968700
Si	-7.07001600	5.25211000	-3.51855700
C	0.30406200	-2.40034600	-2.51838300
H	0.34343100	-1.37285600	-2.86592700
C	0.41393000	-3.45604100	-3.42272500
H	0.53755900	-3.24631800	-4.48040500
C	0.36608100	-4.77891200	-2.98116200
H	0.45531400	-5.58853500	-3.69827800
C	0.20128500	-5.06741700	-1.62890400
H	0.16035900	-6.09695400	-1.28646000
C	0.09026600	-4.01265200	-0.72844600
C	0.14596500	-2.68613000	-1.17113400
C	-0.00000100	-1.70949000	0.00006900
C	-1.25441500	-0.86141300	-0.22985500
C	-2.47830500	-1.25899800	0.26148100
H	-2.56083400	-2.14764300	0.87979000
C	-3.65213100	-0.53580200	-0.02948500
C	-4.91038000	-0.95283000	0.47266900
H	-4.95065800	-1.84087900	1.09174600
C	-6.07404300	-0.27277800	0.19462500
C	-7.34734700	-0.72583900	0.71364600
C	-8.56499900	-0.00699600	0.42966600
C	-9.78133900	-0.42566000	0.92610000
H	-9.81990000	-1.31956700	1.54272000
C	-10.98835600	0.27693300	0.65715700
C	-12.23588300	-0.15012800	1.16291100
H	-12.27533700	-1.04534600	1.77699500
C	-13.38433100	0.55555900	0.88285100

H	-14.33795500	0.21863100	1.27625000
C	-13.32852700	1.71575700	0.08578100
H	-14.23944300	2.26479400	-0.12978200
C	-12.12456400	2.15275600	-0.41898900
H	-12.07572400	3.04608700	-1.03555300
C	-10.93250700	1.44667600	-0.14615800
C	-9.67349800	1.87062000	-0.65415900
H	-9.63242900	2.76880500	-1.26352800
C	-8.50851600	1.18126600	-0.39457600
C	-7.23696600	1.61992600	-0.92965100
C	-6.01319300	0.91030300	-0.63237000
C	-4.78990400	1.32973700	-1.11840200
H	-4.74346000	2.22444700	-1.73262300
C	-3.58686800	0.62423900	-0.83961400
C	-2.32537400	1.02256000	-1.33537300
H	-2.25799200	1.91108400	-1.95703800
C	-1.19311200	0.29900100	-1.04003400
H	-0.23168000	0.62844200	-1.42212300
C	-7.35261400	-1.88525700	1.50700900
C	-7.22897900	-2.89919000	2.18136000
C	-4.88179500	-4.49057600	3.16403400
H	-4.62148500	-5.35048900	3.79926500
C	-4.27062200	-3.23510600	3.80542100
H	-4.55257800	-2.32919600	3.25738200
H	-3.17623000	-3.29362300	3.80250000
H	-4.59778600	-3.10159200	4.84204400
C	-4.28043000	-4.74427300	1.77357500
H	-4.57301300	-5.72045600	1.37404300
H	-3.18432600	-4.71876400	1.81396000

H	-4.60157800	-3.98797700	1.04835500
C	-7.50492300	-5.90179100	2.23908400
H	-6.89455400	-5.97471800	1.32734300
C	-8.96180100	-5.72078800	1.79064400
H	-9.28210000	-6.56583900	1.16978400
H	-9.09151900	-4.80536700	1.20574900
H	-9.64360500	-5.66915100	2.64549400
C	-7.32303700	-7.20655100	3.02798600
H	-7.95288700	-7.21585800	3.92469800
H	-6.28624000	-7.35753600	3.34852900
H	-7.61101400	-8.07352600	2.42214400
C	-7.41432000	-4.14791300	4.92280600
H	-7.10947200	-3.12017200	5.16738000
C	-8.94405100	-4.21150000	5.02733200
H	-9.43203400	-3.56279300	4.29244800
H	-9.28061700	-3.90047000	6.02322600
H	-9.30839100	-5.23243100	4.86712800
C	-6.75375600	-5.08279100	5.94709600
H	-6.99128500	-6.13323600	5.74845500
H	-7.10735400	-4.85641300	6.95990800
H	-5.66354600	-4.98557900	5.95071200
C	-7.19203400	2.75017500	-1.76017300
C	-7.15091900	3.72799500	-2.49426800
C	-5.65753700	5.04327700	-4.78002100
H	-6.11091700	4.49746400	-5.62014300
C	-4.48962800	4.18943900	-4.26331600
H	-4.00807800	4.64500700	-3.39105800
H	-3.72214200	4.07581500	-5.03819000
H	-4.82461300	3.18963700	-3.97232000

C	-5.15708500	6.39157200	-5.32171900
H	-5.96235000	6.98755100	-5.76289100
H	-4.39907400	6.24040900	-6.09895900
H	-4.69662100	6.99498200	-4.53186000
C	-6.75491500	6.69197600	-2.32052100
H	-6.71193900	7.60421500	-2.93352900
C	-5.41330400	6.54152300	-1.58811500
H	-5.37834200	5.60352100	-1.02232100
H	-5.26640400	7.36195300	-0.87582000
H	-4.56217400	6.54818500	-2.27560700
C	-7.89782900	6.84782600	-1.30631200
H	-8.85369600	7.07209300	-1.78923500
H	-7.68518500	7.66302900	-0.60470600
H	-8.02781800	5.93204700	-0.71851000
C	-8.72672900	5.43688700	-4.44178300
H	-8.60944200	4.83120800	-5.35208500
C	-8.99060000	6.88541100	-4.88233000
H	-9.11848500	7.54957200	-4.02065600
H	-9.90743000	6.94813800	-5.47986600
H	-8.17608200	7.29091400	-5.49090800
C	-9.92847900	4.86866500	-3.67173400
H	-9.79337200	3.80615500	-3.44972400
H	-10.84875600	4.97629200	-4.25820400
H	-10.08425800	5.38922800	-2.72035400
Si	6.78125200	-4.40166800	-3.14978400
Si	7.07016700	5.25203500	3.51857100
C	-0.30405200	-2.40031100	2.51853300
H	-0.34344000	-1.37281600	2.86606000
C	-0.41388600	-3.45599400	3.42289400

H	-0.53751100	-3.24625500	4.48057100
C	-0.36600400	-4.77887100	2.98135400
H	-0.45520900	-5.58848400	3.69848400
C	-0.20120800	-5.06739500	1.62909900
H	-0.16025300	-6.09693600	1.28667300
C	-0.09022200	-4.01264200	0.72862200
C	-0.14595600	-2.68611400	1.17128700
C	1.25441500	-0.86141200	0.22997200
C	2.47829000	-1.25900400	-0.26139400
H	2.56079700	-2.14764800	-0.87970700
C	3.65212700	-0.53581800	0.02954700
C	4.91035900	-0.95285700	-0.47263800
H	4.95061300	-1.84090600	-1.09171600
C	6.07403500	-0.27282000	-0.19461600
C	7.34732100	-0.72589900	-0.71366400
C	8.56499100	-0.00708800	-0.42969000
C	9.78131700	-0.42577500	-0.92613800
H	9.81985200	-1.31967800	-1.54276500
C	10.98835300	0.27678600	-0.65719800
C	12.23586600	-0.15030100	-1.16296400
H	12.27529500	-1.04551600	-1.77705500
C	13.38433400	0.55535600	-0.88290600
H	14.33794700	0.21840700	-1.27631300
C	13.32856300	1.71554900	-0.08582600
H	14.23949400	2.26456100	0.12973700
C	12.12461400	2.15257400	0.41895500
H	12.07580000	3.04590000	1.03552700
C	10.93253800	1.44652500	0.14612600
C	9.67354300	1.87049400	0.65414000

H	9.63250100	2.76867600	1.26351600
C	8.50854200	1.18117000	0.39456300
C	7.23700700	1.61985600	0.92965300
C	6.01321800	0.91025600	0.63238900
C	4.78994300	1.32970200	1.11844800
H	4.74352400	2.22440600	1.73268000
C	3.58689300	0.62421900	0.83968200
C	2.32541300	1.02254800	1.33547200
H	2.25805200	1.91106900	1.95714400
C	1.19313700	0.29900000	1.04015600
H	0.23171700	0.62844500	1.42227000
C	7.35254500	-1.88530700	-1.50704200
C	7.22886000	-2.89921600	-2.18141800
C	4.88160200	-4.49045600	-3.16417600
H	4.62126100	-5.35034000	-3.79943200
C	4.27051100	-3.23494100	-3.80555500
H	4.55250600	-2.32905400	-3.25749600
H	3.17611500	-3.29339800	-3.80265700
H	4.59770400	-3.10143000	-4.84216900
C	4.28018900	-4.74415100	-1.77373800
H	4.57270900	-5.72035900	-1.37422100
H	3.18408800	-4.71858100	-1.81415000
H	4.60136000	-3.98788900	-1.04849200
C	7.50463500	-5.90183300	-2.23919700
H	6.89424400	-5.97474200	-1.32746800
C	8.96151500	-5.72092400	-1.79072600
H	9.28175300	-6.56600500	-1.16987600
H	9.09127500	-4.80552100	-1.20581200
H	9.64333800	-5.66931000	-2.64556200

C	7.32268700	-7.20656700	-3.02812800
H	7.95255100	-7.21589300	-3.92482900
H	6.28588600	-7.35748500	-3.34869200
H	7.61060300	-8.07357100	-2.42229800
C	7.41418600	-4.14789800	-4.92288500
H	7.10940300	-3.12013400	-5.16744500
C	8.94391500	-4.21157300	-5.02738100
H	9.43192100	-3.56290800	-4.29247500
H	9.28052000	-3.90054300	-6.02326300
H	9.30819200	-5.23252800	-4.86719000
C	6.75358800	-5.08271700	-5.94720700
H	6.99105000	-6.13318000	-5.74858200
H	7.10722100	-4.85634100	-6.96000800
H	5.66338400	-4.98544000	-5.95084500
C	7.19211100	2.75010200	1.76018000
C	7.15102500	3.72791600	2.49428500
C	5.65767000	5.04325700	4.78002400
H	6.11102500	4.49743700	5.62015400
C	4.48974000	4.18945200	4.26331200
H	4.00821300	4.64503000	3.39104700
H	3.72224300	4.07585500	5.03817900
H	4.82469700	3.18963800	3.97232400
C	5.15725200	6.39157100	5.32170600
H	5.96253100	6.98753100	5.76288000
H	4.39923100	6.24043600	6.09894100
H	4.69681200	6.99498800	4.53183900
C	6.75512600	6.69190800	2.32052600
H	6.71217200	7.60415000	2.93353100
C	5.41351900	6.54149500	1.58810600

H	5.37853500	5.60349400	1.02231200
H	5.26665100	7.36192900	0.87580900
H	4.56238100	6.54818200	2.27558800
C	7.89805700	6.84771900	1.30633000
H	8.85392400	7.07196100	1.78926300
H	7.68544500	7.66292300	0.60471700
H	8.02802600	5.93193200	0.71853500
C	8.72687900	5.43675400	4.44181000
H	8.60956500	4.83107600	5.35210900
C	8.99079100	6.88526900	4.88236500
H	9.11870100	7.54943000	4.02069600
H	9.90761900	6.94796500	5.47990800
H	8.17628100	7.29079200	5.49094000
C	9.92861700	4.86849800	3.67176900
H	9.79347900	3.80599300	3.44975500
H	10.84889200	4.97609400	4.25824800
H	10.08442100	5.38905900	2.72039300

Table S11. The optimized S_0S_0 structure of **FlePhPc2** based on ωB97X-D/6-31G** level of theory.

Si	9.25153400	-5.24738400	1.40246800
Si	10.99758500	5.30836400	-3.51447300
C	-0.26134500	2.36965200	5.35746400
H	-0.29410600	2.74840600	4.34053800
C	-0.35437200	3.24178200	6.44103600
H	-0.46388300	4.30720600	6.26581100
C	-0.30342600	2.75784700	7.74866500
H	-0.37427300	3.45058500	8.58116600
C	-0.15907700	1.39511500	7.99436400
H	-0.11606600	1.02060200	9.01267900
C	-0.07063100	0.52597000	6.91201100
C	-0.12465100	1.01175000	5.60064500
C	0.00441600	-0.12838000	4.58674000
C	4.81393800	1.02144200	1.47470100
C	5.77076400	0.06964400	1.27599300
H	5.63959700	-0.93283500	1.67490200
C	6.95652300	0.34098800	0.51804100
C	7.92757400	-0.62160500	0.32111500
H	7.79480300	-1.60958800	0.74897200
C	9.09671700	-0.35713700	-0.42239500
C	10.09367100	-1.34327600	-0.61178600
C	11.25874400	-1.06424900	-1.36215400
C	12.26219800	-2.03620500	-1.56107400
H	12.12562800	-3.02085200	-1.12355400
C	13.40178500	-1.76430900	-2.29482500
C	14.42914400	-2.74746400	-2.50129800
H	14.29724000	-3.73148000	-2.06074500
C	15.53739100	-2.45349500	-3.22880900

H	16.30702600	-3.20393500	-3.37804000
C	15.70761300	-1.15307900	-3.80827900
H	16.60292600	-0.94372400	-4.38477400
C	14.76315700	-0.19219900	-3.63972200
H	14.88761500	0.79529500	-4.07511300
C	13.57374200	-0.45531200	-2.87784800
C	12.59873700	0.50609500	-2.69012400
H	12.72251300	1.48926400	-3.13377700
C	11.43372100	0.24463100	-1.93853900
C	10.44240100	1.23426900	-1.73803100
C	9.27364800	0.95313800	-0.99392500
C	8.26939800	1.92565400	-0.79112000
H	8.40089400	2.90946500	-1.23115800
C	7.13252100	1.65144000	-0.05610900
C	6.10548500	2.63044800	0.16825600
H	6.23872800	3.62434600	-0.24947900
C	5.00255000	2.33014800	0.89791800
H	4.25028800	3.09145000	1.07944300
C	9.90711800	-2.62650500	-0.02457100
C	9.69719100	-3.70306700	0.50473700
C	7.57255900	-4.91044000	2.22646500
H	7.29629900	-5.83012300	2.76316900
C	6.48683300	-4.62912600	1.17664500
H	6.73606900	-3.74475600	0.57878800
H	5.51881700	-4.44150200	1.65614800
H	6.35442900	-5.46475700	0.48198600
C	7.64227400	-3.76814400	3.24973100
H	8.31884100	-3.99697400	4.07876200
H	6.65276100	-3.56970200	3.67902300

H	7.99323700	-2.83920200	2.78529700
C	10.59376600	-5.54394200	2.71221100
H	10.41183500	-4.76199700	3.46336000
C	12.02470900	-5.34225300	2.19314900
H	12.74712100	-5.42073600	3.01414700
H	12.14894300	-4.35967900	1.72845700
H	12.29452000	-6.09931600	1.44974800
C	10.44266500	-6.90396200	3.40908900
H	10.67565900	-7.72598300	2.72302000
H	9.42845000	-7.06651100	3.79026700
H	11.13144600	-6.98668400	4.25773500
C	9.06546800	-6.62912800	0.11375700
H	8.45298400	-6.17228700	-0.67693900
C	10.40199600	-7.03943500	-0.51910100
H	10.96168100	-6.17421300	-0.88949600
H	10.24150800	-7.71968400	-1.36358800
H	11.03607600	-7.56501100	0.20369400
C	8.30520200	-7.85650100	0.63867700
H	8.85465100	-8.35937300	1.44126700
H	8.15799400	-8.59031300	-0.16241000
H	7.31681100	-7.59472500	1.02957100
C	10.62860400	2.53442800	-2.28608000
C	10.79329900	3.65068800	-2.74409600
C	10.14017800	6.59146400	-2.39708200
H	10.89498400	6.85413800	-1.64174800
C	8.92076800	6.03760000	-1.64455000
H	8.13593900	5.70414900	-2.33238500
H	8.48365000	6.80773800	-0.99784500
H	9.19394400	5.18552600	-1.01542000

C	9.78100100	7.87804800	-3.15709400
H	10.65044000	8.32984700	-3.64523900
H	9.36303600	8.62761800	-2.47518200
H	9.03023100	7.68967600	-3.93223600
C	10.19700700	5.20358200	-5.23318700
H	10.31909300	6.19394800	-5.69581200
C	8.69457800	4.89695200	-5.14386300
H	8.51785200	3.95063500	-4.62009100
H	8.25740300	4.80628200	-6.14518300
H	8.14197000	5.67917100	-4.61482700
C	10.89701900	4.16710000	-6.12475200
H	11.94614700	4.41797900	-6.30775300
H	10.40134200	4.09697900	-7.10013900
H	10.86658300	3.17178000	-5.66707000
C	12.86131600	5.69238200	-3.61027400
H	13.10933700	6.12417200	-2.62981400
C	13.18350400	6.75202100	-4.67578700
H	12.95796000	6.38833000	-5.68418800
H	14.24811600	7.01224900	-4.65476500
H	12.61838000	7.67763100	-4.52680500
C	13.73988900	4.44513600	-3.79078500
H	13.59387000	3.73300500	-2.97346100
H	14.80112200	4.72034000	-3.81257800
H	13.51787400	3.92404300	-4.72858200
Si	-9.21429800	5.21692000	1.58819800
Si	-11.04381600	-5.15247900	-3.68518000
C	0.27544100	-2.67112500	5.19198000
H	0.30754900	-2.98278600	4.15255600
C	0.37132000	-3.61220700	6.21587700

H	0.48221300	-4.66366700	5.97099900
C	0.32166000	-3.21514800	7.55245800
H	0.39486400	-3.96084900	8.33763900
C	0.17612600	-1.87163700	7.88713100
H	0.13461400	-1.56464200	8.92785500
C	0.08481400	-0.93353300	6.86415100
C	0.13696500	-1.33230100	5.52372300
C	-4.81556100	-1.09423200	1.42921400
C	-5.76701800	-0.12931200	1.27247900
H	-5.62892900	0.85459200	1.71319600
C	-6.95559100	-0.36197000	0.50588900
C	-7.92051700	0.61409700	0.34842600
H	-7.78076200	1.58373000	0.81449200
C	-9.09230900	0.38678700	-0.40330700
C	-10.08211500	1.38669400	-0.55436500
C	-11.25057000	1.14443100	-1.31232100
C	-12.24684600	2.13065300	-1.47376300
H	-12.10195100	3.09777600	-1.00126400
C	-13.38987600	1.89431300	-2.21444400
C	-14.40977200	2.89233600	-2.38311100
H	-14.26920400	3.85877000	-1.90768300
C	-15.52165800	2.63356700	-3.11841400
H	-16.28554300	3.39495000	-3.23893900
C	-15.70333800	1.35622300	-3.74396600
H	-16.60140500	1.17484100	-4.32563600
C	-14.76624800	0.38246000	-3.61216000
H	-14.89940200	-0.58771700	-4.08250200
C	-13.57329800	0.60861400	-2.84400600
C	-12.60537200	-0.36637800	-2.69346900

H	-12.73744400	-1.33189100	-3.17216400
C	-11.43678400	-0.14110400	-1.93579500
C	-10.45230300	-1.14456500	-1.77452100
C	-9.27966800	-0.89951000	-1.02388300
C	-8.28130100	-1.88549100	-0.86184900
H	-8.42013000	-2.85043200	-1.33978200
C	-7.14087800	-1.64734600	-0.11992600
C	-6.11922800	-2.64073900	0.06121100
H	-6.25958400	-3.61591300	-0.39641800
C	-5.01256400	-2.37683500	0.79914900
H	-4.26386500	-3.14883400	0.94709900
C	-9.88490500	2.64654800	0.07817800
C	-9.66749800	3.70292200	0.64389900
C	-7.54168600	4.84342100	2.40911300
H	-7.26193200	5.74350100	2.97640200
C	-6.45272600	4.58833000	1.35593300
H	-6.70514700	3.72524100	0.72900200
H	-5.48840900	4.37863200	1.83372800
H	-6.31121700	5.44484500	0.68907700
C	-7.62421300	3.66915400	3.39454700
H	-8.30465700	3.87494100	4.22644600
H	-6.63855700	3.45098600	3.82307600
H	-7.97763400	2.75794500	2.89792400
C	-10.56057800	5.48661100	2.89968800
H	-10.38620700	4.68458900	3.63122300
C	-11.99021300	5.30624100	2.36921800
H	-12.71603000	5.36846000	3.18860800
H	-12.11789600	4.33633900	1.87953000
H	-12.25211700	6.08323600	1.64373000

C	-10.40583000	6.82734800	3.63230200
H	-10.63244200	7.66797200	2.96695700
H	-9.39240200	6.97524000	4.02145600
H	-11.09752900	6.89117000	4.48020700
C	-9.01250000	6.63294700	0.33964400
H	-8.39833800	6.19426400	-0.45998700
C	-10.34299800	7.06846700	-0.28898900
H	-10.90509300	6.21704600	-0.68668800
H	-10.17391400	7.77120800	-1.11314300
H	-10.97849400	7.57709300	0.44458800
C	-8.24777300	7.84059600	0.90233400
H	-8.79928800	8.32544400	1.71448800
H	-8.09035800	8.59462100	0.12224400
H	-7.26379500	7.56176800	1.29254900
C	-10.64981000	-2.42277200	-2.36835700
C	-10.82408000	-3.52131500	-2.86410600
C	-10.19515100	-6.47596600	-2.60901600
H	-10.95026800	-6.75345100	-1.85929500
C	-8.96823800	-5.95639100	-1.84432500
H	-8.18221600	-5.61026300	-2.52448900
H	-8.53670800	-6.74952800	-1.22213800
H	-9.23130300	-5.12081200	-1.18931300
C	-9.85083200	-7.74309500	-3.40758700
H	-10.72611000	-8.17186700	-3.90594300
H	-9.43830900	-8.51640200	-2.74926700
H	-9.10046300	-7.53950400	-4.17925800
C	-10.24633300	-5.00178600	-5.40186600
H	-10.37894900	-5.97606200	-5.89476400
C	-8.74085400	-4.71209000	-5.30749900

H	-8.55379200	-3.78438700	-4.75479600
H	-8.30562900	-4.59399400	-6.30680800
H	-8.19408600	-5.51567000	-4.80507800
C	-10.93882200	-3.93146200	-6.25869300
H	-11.99098200	-4.16609200	-6.44575100
H	-10.44552500	-3.83619400	-7.23315200
H	-10.89702200	-2.95107700	-5.77067500
C	-12.91090300	-5.51824800	-3.78818200
H	-13.15976200	-5.97960600	-2.82148400
C	-13.24468800	-6.54019100	-4.88647800
H	-13.01895800	-6.14586600	-5.88324700
H	-14.31134900	-6.79225900	-4.87064300
H	-12.68681700	-7.47473100	-4.76935300
C	-13.77983600	-4.25882800	-3.92557200
H	-13.62558600	-3.57457600	-3.08624600
H	-14.84333200	-4.52464500	-3.95279600
H	-13.55637100	-3.70962500	-4.84681900
C	-3.59267700	-0.84329800	2.22826900
C	-2.34774700	-1.33078500	1.81690400
C	-1.20106400	-1.07385400	2.55518300
C	-1.25412500	-0.32145900	3.73222800
C	-2.49674000	0.15568700	4.14969800
C	-3.64519500	-0.10169700	3.41164600
H	-2.26853800	-1.89455800	0.89211400
H	-0.24564200	-1.44576700	2.19828800
H	-2.57266700	0.72665000	5.06877900
H	-4.60297000	0.26146000	3.77201000
C	2.34647100	1.23248100	1.88134800
C	3.59396100	0.72855700	2.26391200

C	3.65161800	-0.07261500	3.40782300
C	2.50561000	-0.37353900	4.13263000
C	1.26006400	0.11888400	3.74210600
C	1.20205200	0.93308200	2.60742100
H	2.26287700	1.84318900	0.98725400
H	4.61138900	-0.44974800	3.74790900
H	2.58514700	-0.99191500	5.02024700
H	0.24465700	1.32006900	2.27254500

Table S12. The optimized S_1S_0 structure of **FlePhPc2** based on ωB97X-D/6-31G** level of theory.

Si	9.25018900	-5.22753100	1.43637300
Si	11.05352700	5.28334300	-3.52057500
C	-0.26725600	2.37639400	5.33411200
H	-0.30518900	2.75743900	4.31822500
C	-0.35741800	3.24594500	6.41999500
H	-0.46977600	4.31152500	6.24758400
C	-0.30004500	2.75921600	7.72631400
H	-0.36872800	3.44996000	8.56064400
C	-0.15211200	1.39621500	7.96837600
H	-0.10419700	1.01953000	8.98566600
C	-0.06652900	0.52962800	6.88375300
C	-0.12689900	1.01821500	5.57370200
C	0.00037700	-0.11950600	4.55678200
C	4.79862200	1.05272500	1.43712600
C	5.77650200	0.09087900	1.24753000
H	5.64292700	-0.90778400	1.65522700
C	6.94398800	0.36285600	0.50565400
C	7.94617000	-0.61278500	0.31095800
H	7.80767700	-1.59661100	0.74756900
C	9.10209100	-0.35562800	-0.41396100
C	10.11389200	-1.35029500	-0.59420800
C	11.29783700	-1.08007000	-1.34603000
C	12.28471200	-2.04091700	-1.52914800
H	12.14828100	-3.02325300	-1.08541100
C	13.45743900	-1.78366100	-2.27335000
C	14.46304800	-2.76079900	-2.45874400
H	14.32890800	-3.74201400	-2.01239200
C	15.59326800	-2.47576500	-3.19151600

H	16.35774000	-3.23392600	-3.32654900
C	15.76449300	-1.20070500	-3.76931900
H	16.65950600	-0.98699400	-4.34443600
C	14.80251900	-0.22929400	-3.60581800
H	14.93088000	0.75491800	-4.04772100
C	13.63139900	-0.49265000	-2.85859000
C	12.62644600	0.48232900	-2.67028500
H	12.75640700	1.46202800	-3.12086200
C	11.47767900	0.22938000	-1.93249100
C	10.47235700	1.22890400	-1.73725600
C	9.28414600	0.95579500	-0.99531400
C	8.29332500	1.91581500	-0.81288700
H	8.42570100	2.89729100	-1.25888500
C	7.12184600	1.65577000	-0.07138100
C	6.11341100	2.62679500	0.12770200
H	6.24718700	3.61734500	-0.29802200
C	4.98745100	2.33456000	0.86066500
H	4.23925400	3.10340300	1.02576000
C	9.92272300	-2.61774900	-0.00529500
C	9.70562900	-3.69363000	0.53167400
C	7.57033500	-4.87787800	2.25453400
H	7.28947700	-5.79198200	2.79839700
C	6.48780600	-4.60118800	1.20029700
H	6.74306800	-3.72419600	0.59405400
H	5.51970700	-4.40398100	1.67600400
H	6.35256200	-5.44278700	0.51344200
C	7.64246900	-3.72736900	3.26829900
H	8.31648100	-3.95198500	4.10056400
H	6.65291100	-3.52100700	3.69401600

H	7.99893100	-2.80412300	2.79665000
C	10.58596800	-5.52733600	2.75349300
H	10.40431000	-4.74195900	3.50107100
C	12.01962000	-5.33346200	2.23923600
H	12.73895300	-5.41270000	3.06294100
H	12.14910300	-4.35267600	1.77210000
H	12.28871600	-6.09378700	1.49882500
C	10.42646900	-6.88414900	3.45462900
H	10.66030000	-7.70961700	2.77302300
H	9.40969300	-7.04175100	3.83104100
H	11.11048500	-6.96593500	4.30730700
C	9.05933700	-6.62003100	0.15837900
H	8.44996500	-6.16666700	-0.63660700
C	10.39501900	-7.04047000	-0.46936300
H	10.95797900	-6.17999600	-0.84586300
H	10.23326800	-7.72692700	-1.30865400
H	11.02645200	-7.56245400	0.25835700
C	8.29298300	-7.84007400	0.69146500
H	8.83943800	-8.34015900	1.49776700
H	8.14259700	-8.57865000	-0.10473900
H	7.30562500	-7.57085800	1.07992800
C	10.66497900	2.51529800	-2.28291800
C	10.83715400	3.63282600	-2.74538700
C	10.18044500	6.57504900	-2.42440600
H	10.92475100	6.84177600	-1.66013000
C	8.95048100	6.02601800	-1.68582000
H	8.17520300	5.68907300	-2.38279100
H	8.50463700	6.79971200	-1.04927400
H	9.21509400	5.17710200	-1.04870300

C	9.83250400	7.85728500	-3.19676700
H	10.70872100	8.30525500	-3.67624900
H	9.40641700	8.61148200	-2.52494700
H	9.09175800	7.66506800	-3.98055900
C	10.28025100	5.17641900	-5.25298600
H	10.41020000	6.16617800	-5.71494700
C	8.77669600	4.87008900	-5.18682900
H	8.59237400	3.92460700	-4.66412000
H	8.35495000	4.77786700	-6.19467900
H	8.21586600	5.65301700	-4.66750800
C	10.99407000	4.13814800	-6.13120100
H	12.04601200	4.38863200	-6.29812900
H	10.51390600	4.06568200	-7.11422300
H	10.95633400	3.14401800	-5.67136800
C	12.91902700	5.66715900	-3.59096300
H	13.15206400	6.10195600	-2.60815900
C	13.25805300	6.72314300	-4.65476200
H	13.04702800	6.35672000	-5.66531500
H	14.32253500	6.98257800	-4.61887300
H	12.69163100	7.64967600	-4.51685500
C	13.79950700	4.41892700	-3.75365500
H	13.63826900	3.70894900	-2.93723800
H	14.86133800	4.69292500	-3.75797300
H	13.59292200	3.89604900	-4.69402900
Si	-9.23758900	5.21558800	1.59648100
Si	-11.06920900	-5.14929100	-3.68536100
C	0.27737700	-2.66320200	5.15534700
H	0.30545000	-2.97263900	4.11514100
C	0.37897800	-3.60634300	6.17680000

H	0.49034900	-4.65710400	5.92917900
C	0.33440000	-3.21222300	7.51443100
H	0.41203600	-3.95949300	8.29768200
C	0.18823600	-1.86966000	7.85261500
H	0.15058200	-1.56498700	8.89416500
C	0.09120700	-0.92949300	6.83206800
C	0.13838100	-1.32529100	5.49057400
C	-4.82810900	-1.08743100	1.41314700
C	-5.78200700	-0.12412100	1.26147200
H	-5.64434000	0.85926500	1.70348600
C	-6.97282400	-0.35781300	0.49868000
C	-7.94024600	0.61659000	0.34632500
H	-7.80078100	1.58572300	0.81352300
C	-9.11430500	0.38817100	-0.40152900
C	-10.10660100	1.38637200	-0.54742800
C	-11.27741100	1.14293500	-1.30136800
C	-12.27622000	2.12743300	-1.45761500
H	-12.13144000	3.09415400	-0.98426000
C	-13.42158600	1.88991400	-2.19430000
C	-14.44406000	2.88618000	-2.35768000
H	-14.30357100	3.85221600	-1.88142300
C	-15.55822100	2.62627100	-3.08912600
H	-16.32403100	3.38633400	-3.20569000
C	-15.73979400	1.34945200	-3.71578100
H	-16.63971900	1.16713400	-4.29427700
C	-14.80031700	0.37733700	-3.58891300
H	-14.93337300	-0.59244200	-4.06010000
C	-13.60488600	0.60475200	-2.82500000
C	-12.63447800	-0.36854300	-2.67957200

H	-12.76649700	-1.33362700	-3.15914600
C	-11.46344900	-0.14205000	-1.92604000
C	-10.47634200	-1.14377000	-1.77005300
C	-9.30139400	-0.89750700	-1.02345300
C	-8.30043500	-1.88172000	-0.86677600
H	-8.43909300	-2.84612100	-1.34584700
C	-7.15777500	-1.64250300	-0.12865200
C	-6.13348100	-2.63414700	0.04706200
H	-6.27352200	-3.60879900	-0.41177300
C	-5.02472100	-2.36931500	0.78150800
H	-4.27394900	-3.14005700	0.92544900
C	-9.90941800	2.64572100	0.08612300
C	-9.69178600	3.70175800	0.65238500
C	-7.56161000	4.84385200	2.41128400
H	-7.28132800	5.74379100	2.97853500
C	-6.47584100	4.59146700	1.35417400
H	-6.72901700	3.72853000	0.72733500
H	-5.50955900	4.38289700	1.82848300
H	-6.33800000	5.44880200	0.68760500
C	-7.63903600	3.66846600	3.39579600
H	-8.31713000	3.87230300	4.23009100
H	-6.65165500	3.45144700	3.82092600
H	-7.99259800	2.75719800	2.89937600
C	-10.57961000	5.48223800	2.91293100
H	-10.40120500	4.68014600	3.64342400
C	-12.01083100	5.29969600	2.38751100
H	-12.73381100	5.36040800	3.20951600
H	-12.13866200	4.32977400	1.89790700
H	-12.27660300	6.07653300	1.66326000

C	-10.42452500	6.82285500	3.64570000
H	-10.65498300	7.66344700	2.98164000
H	-9.40995200	6.97228800	4.03126900
H	-11.11326300	6.88502500	4.49613200
C	-9.04226600	6.63291700	0.34841000
H	-8.43036400	6.19578600	-0.45380000
C	-10.37567100	7.06700200	-0.27505900
H	-10.93787900	6.21509600	-0.67156500
H	-10.21060100	7.77077600	-1.09914400
H	-11.00932000	7.57395800	0.46126800
C	-8.27728400	7.84123300	0.90932200
H	-8.82663500	8.32468200	1.72377300
H	-8.12368500	8.59606800	0.12925600
H	-7.29153800	7.56352300	1.29587100
C	-10.67357400	-2.42142800	-2.36515700
C	-10.84783700	-3.51944300	-2.86207600
C	-10.21077900	-6.47329900	-2.61761500
H	-10.96050600	-6.75420300	-1.86377200
C	-8.98024600	-5.95264700	-1.85949100
H	-8.19946900	-5.60280500	-2.54377900
H	-8.54278400	-6.74629900	-1.24212200
H	-9.24118600	-5.11941400	-1.20064700
C	-9.86862800	-7.73787500	-3.42116100
H	-10.74602100	-8.16745100	-3.91508000
H	-9.45038400	-8.51174900	-2.76713200
H	-9.12346100	-7.53095000	-4.19697600
C	-10.28320300	-4.99302500	-5.40682900
H	-10.41689600	-5.96646000	-5.90109100
C	-8.77776900	-4.70028000	-5.32151000

H	-8.58916000	-3.77339900	-4.76795600
H	-8.34923300	-4.57903700	-6.32333100
H	-8.22604700	-5.50378500	-4.82441000
C	-10.98350200	-3.92226000	-6.25673200
H	-12.03630900	-4.15879500	-6.43763400
H	-10.49664900	-3.82365500	-7.23409600
H	-10.94075400	-2.94290000	-5.76673900
C	-12.93614200	-5.51888100	-3.77693100
H	-13.17760900	-5.98282100	-2.80959300
C	-13.27487600	-6.53926200	-4.87516600
H	-13.05671900	-6.14227300	-5.87256100
H	-14.34082200	-6.79384700	-4.85277700
H	-12.71407800	-7.47275100	-4.76378200
C	-13.80880000	-4.26115900	-3.90593000
H	-13.65036200	-3.57814600	-3.06637600
H	-14.87185700	-4.52931600	-3.92642600
H	-13.59286800	-3.70973300	-4.82764300
C	-3.60298300	-0.83558300	2.20848300
C	-2.35850800	-1.31974400	1.79185100
C	-1.20986900	-1.06229100	2.52689800
C	-1.26049900	-0.31270600	3.70583700
C	-2.50257200	0.16126800	4.12846300
C	-3.65297300	-0.09663300	3.39363100
H	-2.28136000	-1.88137800	0.86559000
H	-0.25496200	-1.43175100	2.16606000
H	-2.57649500	0.73001400	5.04908200
H	-4.61024000	0.26391800	3.75792700
C	2.33096300	1.25209100	1.84700600
C	3.58104500	0.74972700	2.22422800

C	3.64302100	-0.05776400	3.36392600
C	2.50015000	-0.36116200	4.09246900
C	1.25282900	0.13224300	3.70885300
C	1.18991300	0.94991700	2.57674600
H	2.24393000	1.86299800	0.95348900
H	4.60336300	-0.43729600	3.69985200
H	2.58345300	-0.98333200	4.97704900
H	0.23080200	1.33613200	2.24597000

Table S13. The optimized [1]TT structure of **FlePhPc2** based on ωB97X-D/6-31G** level of theory.

C	19.17572300	11.89501200	-3.38195900
C	20.13509700	10.93806800	-3.57655100
H	20.00089400	9.93773100	-3.17321500
C	21.31680200	11.20537100	-4.33133000
C	22.29369200	10.23535700	-4.52575800
H	22.15700700	9.24967700	-4.09363600
C	23.45900000	10.49428500	-5.26540600
C	24.45828500	9.50028900	-5.45043900
C	25.62990800	9.77384700	-6.20447800
C	26.62523100	8.80123400	-6.39743500
H	26.48644300	7.81851300	-5.95616900
C	27.77414900	9.06531500	-7.13637200
C	28.79262000	8.08002800	-7.33590000
H	28.65754400	7.09815800	-6.89143100
C	29.90656800	8.36663700	-8.06635600
H	30.67258900	7.61162200	-8.21093000
C	30.08016900	9.65864900	-8.64846500
H	30.97546300	9.86535100	-9.22592800
C	29.13414400	10.62499300	-8.48236800
H	29.26175500	11.61036900	-8.92172300
C	27.94951200	10.36645100	-7.72247000
C	26.96935600	11.33569200	-7.53596300
H	27.09713600	12.31654600	-7.98372900
C	25.80902600	11.08047900	-6.78653200
C	24.81607100	12.07827100	-6.58932900
C	23.64021700	11.80237900	-5.84231300
C	22.64341400	12.77506700	-5.64684300
H	22.77740100	13.75703700	-6.09037200

C	21.49633800	12.50814400	-4.90829900
C	20.47706600	13.48873200	-4.69243200
H	20.61316900	14.48047100	-5.11444400
C	19.36774800	13.19514100	-3.96120600
H	18.61839500	13.96066300	-3.78544700
C	24.26825600	8.22353600	-4.85938800
C	24.05537500	7.14878700	-4.32525300
C	25.00650800	13.37250600	-7.14014900
C	25.17523800	14.48752400	-7.60239200
Si	23.60722000	5.60851600	-3.42196300
C	21.92865900	5.95099900	-2.59947800
H	21.65097000	5.03382100	-2.05923800
C	20.84349500	6.22990600	-3.65050900
H	21.09417300	7.11165000	-4.25164700
H	19.87570100	6.42082600	-3.17187600
H	20.70994500	5.39184400	-4.34200800
C	21.99999000	7.09716800	-1.58066300
H	22.67626900	6.87065500	-0.75076400
H	21.01077500	7.29868800	-1.15214200
H	22.35222500	8.02378000	-2.04878200
C	24.94884200	5.31436300	-2.11100000
H	24.76764800	6.09868800	-1.36216000
C	26.38014600	5.51283500	-2.63031900
H	27.10228200	5.43569900	-1.80895200
H	26.50576200	6.49395500	-3.09770200
H	26.64915300	4.75338400	-3.37157400
C	24.79605100	3.95655600	-1.41017100
H	25.02840600	3.13227700	-2.09374300
H	23.78154900	3.79619300	-1.02883400

H	25.48447400	3.87562500	-0.56106500
C	23.41944400	4.22255800	-4.70594600
H	22.80759000	4.67745500	-5.49824900
C	24.75550800	3.80843700	-5.33729600
H	25.31625800	4.67169500	-5.71065200
H	24.59424400	3.12546700	-6.17943400
H	25.38892400	3.28460000	-4.61266300
C	22.65758100	2.99793900	-4.17692700
H	23.20638100	2.49701000	-3.37268700
H	22.50940800	2.26167500	-4.97557900
H	21.66953600	3.26228900	-3.78690100
Si	25.38462200	16.14119300	-8.37973600
C	24.53067400	17.43164300	-7.26817200
H	25.28606600	17.69537400	-6.51379000
C	23.30944100	16.88465200	-6.51357600
H	22.52371700	16.55078600	-7.20018700
H	22.87456900	17.65880000	-5.87015800
H	23.57982800	16.03432800	-5.88088800
C	24.17546800	18.71598900	-8.03382000
H	25.04632300	19.16307900	-8.52377300
H	23.75963500	19.46973800	-7.35522100
H	23.42425400	18.52651900	-8.80826200
C	24.58420000	16.03154600	-10.09827400
H	24.70917100	17.01964800	-10.56495100
C	23.08088900	15.72951000	-10.00806000
H	22.90137000	14.78581800	-9.48051100
H	22.64369100	15.63601600	-11.00911000
H	22.53034300	16.51539600	-9.48232600
C	25.28144000	14.98939600	-10.98538400

H	26.33132700	15.23646000	-11.16916400
H	24.78579300	14.91668300	-11.96059600
H	25.24801800	13.99604900	-10.52363000
C	27.24952700	16.51925800	-8.47656600
H	27.49853700	16.95414000	-7.49772200
C	27.57515600	17.57378500	-9.54610300
H	27.34874500	17.20688600	-10.55314500
H	28.64054700	17.83086200	-9.52582700
H	27.01281600	18.50167500	-9.40082800
C	28.12446800	15.26873900	-8.65193900
H	27.97605100	14.56019800	-7.83193100
H	29.18651500	15.54072400	-8.67439100
H	27.90126900	14.74468000	-9.58779600
C	17.95515100	11.60424400	-2.59275500
C	18.01218100	10.80684800	-1.44617800
H	18.97153500	10.42992100	-1.10463000
C	16.86593800	10.50925500	-0.72031500
H	16.94503500	9.89383600	0.16939400
C	15.62070300	11.00093900	-1.11272000
C	15.56325000	11.81100200	-2.25040000
H	14.60604300	12.19699100	-2.58696900
C	16.70786500	12.10692200	-2.97757600
H	16.62494600	12.71383300	-3.87429800
C	14.36469000	10.75692200	-0.26768100
C	14.23538300	11.90060700	0.74217500
C	14.28847900	11.41938600	2.05526100
C	14.19957000	12.29231500	3.13452600
H	14.24185500	11.92134900	4.15417100
C	14.05571300	13.65421600	2.88398400

H	13.98450600	14.34987200	3.71401900
C	14.00574000	14.13360800	1.57464600
H	13.89664500	15.19845100	1.39564800
H	14.06726600	13.63287200	-0.52410100
C	14.09922900	13.25768700	0.49417500
C	14.49657400	9.55625600	0.67358000
C	14.44366600	9.95969600	2.01257300
C	14.53420700	9.02516000	3.03888800
H	14.49209700	9.33579700	4.07850700
C	14.67976400	7.68047000	2.70900000
H	14.75237600	6.93750100	3.49682400
C	14.73020600	7.27874800	1.37384400
H	14.84111400	6.22642300	1.13270800
C	14.63507500	8.21625900	0.34660700
H	14.66778600	7.90096800	-0.69170000
C	13.10643900	10.56096600	-1.12200700
C	11.86381300	11.04035300	-0.70718100
H	11.78763400	11.61519200	0.20945800
C	10.71564300	10.78054900	-1.44488900
H	9.75799200	11.14597600	-1.08643700
C	10.76839800	10.03373900	-2.62497400
C	12.01348100	9.54480800	-3.03410200
H	12.09266500	8.97787600	-3.95695100
C	13.15984000	9.80405200	-2.29615000
H	14.11536400	9.43068500	-2.65125900
C	9.54537000	9.77805100	-3.42242200
C	8.59096900	10.74619400	-3.58296200
H	8.73145500	11.73143200	-3.14591800
C	7.40671200	10.51053700	-4.34450000

C	6.43546200	11.49211600	-4.50724600
H	6.57866300	12.46316600	-4.04501400
C	5.26763700	11.26337000	-5.25301200
C	4.27501600	12.26920500	-5.40769900
C	3.10002100	12.02557800	-6.16685200
C	2.11130100	13.01029200	-6.33032600
H	2.25801000	13.97935800	-5.86226500
C	0.95889500	12.77511300	-7.07358700
C	-0.05273200	13.77295600	-7.24334800
H	0.09057800	14.74116100	-6.77226600
C	-1.17036300	13.51482900	-7.97878500
H	-1.93108400	14.27914200	-8.10066800
C	-1.35482300	12.24066500	-8.59589600
H	-2.25293500	12.05654300	-9.17661800
C	-0.41558800	11.26320100	-8.45883400
H	-0.55146500	10.29128000	-8.92486500
C	0.77265500	11.49202000	-7.69508700
C	1.74627600	10.51104400	-7.53812800
H	1.61060500	9.54393200	-8.01270300
C	2.91023200	10.73717700	-6.78504500
C	3.89689400	9.72759400	-6.61917100
C	5.07667400	9.97424800	-5.86821400
C	6.06821200	8.99033900	-5.70553800
H	5.92740100	8.02337300	-6.17890400
C	7.21869500	9.22778300	-4.96238900
C	8.23312500	8.23522800	-4.78159900
H	8.09053900	7.25860700	-5.23552100
C	9.34584200	8.49895700	-4.04430800
H	10.09205700	7.72463800	-3.89585600

C	4.47521000	13.52779400	-4.78210700
C	4.69562300	14.58683100	-4.22035400
C	3.69566300	8.45028200	-7.20460000
C	3.51773200	7.34882800	-7.69504200
Si	5.15291900	16.10343200	-3.28234700
C	6.82697500	15.73054300	-2.46414200
H	7.10948100	16.63238100	-1.90103600
C	7.91290000	15.46971400	-3.51902900
H	7.65767300	14.60443000	-4.14179000
H	8.87814400	15.26061500	-3.04286400
H	8.05386100	16.32332400	-4.18971800
C	6.74509500	14.56023900	-1.47395900
H	6.06718400	14.77037600	-0.64108300
H	7.73150600	14.34206300	-1.04718600
H	6.38886800	13.64770400	-1.96613700
C	3.81018300	16.37947900	-1.96852200
H	3.98519800	15.57973400	-1.23465800
C	2.37896600	16.19942600	-2.49480200
H	1.65531100	16.26539300	-1.67379700
H	2.24861100	15.22810900	-2.98097200
H	2.11634100	16.97439500	-3.22218400
C	3.96879300	17.72251600	-1.24096700
H	3.74199300	18.56117200	-1.90873000
H	4.98336800	17.87011300	-0.85470200
H	3.27918100	17.79041500	-0.39168300
C	5.35347900	17.51499200	-4.53619700
H	5.96476700	17.07277000	-5.33607900
C	4.02193500	17.95055900	-5.16259100
H	3.45734600	17.09875600	-5.55592200

H	4.18987700	18.65029400	-5.98952800
H	3.38928600	18.46263500	-4.42895400
C	6.12162600	18.72329800	-3.97957300
H	5.57330400	19.21144300	-3.16722200
H	6.27769300	19.47467600	-4.76248200
H	7.10641800	18.44427900	-3.59155200
Si	3.29299800	5.71490200	-8.50901100
C	4.14232600	4.39392000	-7.43020700
H	3.38826800	4.11931800	-6.67836700
C	5.37098600	4.91484700	-6.66924300
H	6.15604700	5.25832200	-7.35187000
H	5.80302800	4.12325500	-6.04545100
H	5.10978900	5.75262600	-6.01630400
C	4.48437500	3.12405800	-8.22541700
H	3.60810200	2.69462900	-8.72144500
H	4.89696600	2.35234500	-7.56527400
H	5.23399500	3.32460400	-8.99860600
C	4.08639000	5.85720400	-10.22837100
H	3.95212800	4.88071000	-10.71640800
C	5.59225600	6.14642900	-10.13867000
H	5.78104300	7.07679900	-9.59106700
H	6.02544100	6.25917500	-11.13948200
H	6.13961200	5.34510000	-9.63330500
C	3.39266100	6.92390700	-11.08867200
H	2.34010900	6.68872300	-11.27275600
H	3.88419800	7.01468300	-12.06445000
H	3.43564300	7.90648000	-10.60515700
C	1.42496900	5.35228500	-8.60530000
H	1.17767600	4.89715400	-7.63525200

C	1.08654300	4.32445600	-9.69664700
H	1.31098500	4.71220100	-10.69628300
H	0.01936500	4.07485400	-9.67699400
H	1.64258300	3.38940900	-9.57497400
C	0.55807100	6.61251400	-8.74794600
H	0.71589200	7.30151600	-7.91318200
H	-0.50599000	6.34855600	-8.77066500
H	0.78001800	7.15573800	-9.67308900

Table S14. The optimized ^{5}TT structure of **FlePhPc2** based on ωB97X-D/6-31G** level of theory.

Si	9.25347600	-5.24744300	1.42682300
Si	11.08509900	5.28640100	-3.50422800
C	-0.27421100	2.39881600	5.31822500
H	-0.30770000	2.77329600	4.29974000
C	-0.37247500	3.27483300	6.39819600
H	-0.48685900	4.33901900	6.21858700
C	-0.32052800	2.79640900	7.70781100
H	-0.39551200	3.49214500	8.53744900
C	-0.16988800	1.43538500	7.95912100
H	-0.12606900	1.06517100	8.97897600
C	-0.07622000	0.56234300	6.88034800
C	-0.13131000	1.04256600	5.56697500
C	0.00416400	-0.10098300	4.55777000
C	4.81194800	1.06322800	1.44740900
C	5.78350300	0.09811400	1.25259500
H	5.64560100	-0.90169100	1.65626400
C	6.95034300	0.37036500	0.51130000
C	7.95451600	-0.61486600	0.31019600
H	7.81063400	-1.59970400	0.74288800
C	9.10090000	-0.35744700	-0.40975200
C	10.11807700	-1.36972600	-0.59631400
C	11.31239200	-1.09909900	-1.36221900
C	12.28135600	-2.06192700	-1.55103400
H	12.14145700	-3.04550700	-1.11123300
C	13.45984200	-1.80926200	-2.30587400
C	14.45408900	-2.79363700	-2.49874900
H	14.31455500	-3.77541800	-2.05515300
C	15.58239200	-2.51466000	-3.23645500

H	16.34053100	-3.27799300	-3.37862600
C	15.75921500	-1.23973300	-3.80940200
H	16.65241500	-1.02951100	-4.38878100
C	14.80466500	-0.26318900	-3.63592100
H	14.93645100	0.72226100	-4.07417100
C	13.63839400	-0.52445500	-2.88358000
C	12.63454600	0.46238500	-2.68326400
H	12.77017100	1.44308700	-3.12993700
C	11.49780700	0.21168200	-1.94483400
C	10.49133300	1.23004600	-1.73864500
C	9.28893300	0.95512100	-0.98630900
C	8.31463600	1.91543300	-0.80251700
H	8.45121700	2.89890000	-1.24278500
C	7.13350900	1.65736300	-0.05626000
C	6.13426600	2.63446200	0.14621200
H	6.27406000	3.62584100	-0.27574900
C	5.00718600	2.34588600	0.87834800
H	4.26249800	3.11757700	1.04692300
C	9.92460500	-2.63124900	-0.01168000
C	9.70650700	-3.70938000	0.52402500
C	7.57334500	-4.90145500	2.24467900
H	7.29303500	-5.81757000	2.78536100
C	6.49119600	-4.62223600	1.19067600
H	6.74480400	-3.74157800	0.58913400
H	5.52249600	-4.42922000	1.66661300
H	6.35813500	-5.46089200	0.49981200
C	7.64389500	-3.75411600	3.26218900
H	8.31813800	-3.98036200	4.09381100
H	6.65404100	-3.55069000	3.68830700

H	7.99836700	-2.82861300	2.79351700
C	10.59079700	-5.54444100	2.74196000
H	10.40883000	-4.75981900	3.49028000
C	12.02404700	-5.34882100	2.22699800
H	12.74358300	-5.42792900	3.05045400
H	12.15322700	-4.36774900	1.76046300
H	12.29361400	-6.10845300	1.48613000
C	10.43322100	-6.90195500	3.44229500
H	10.66654800	-7.72668700	2.75962100
H	9.41714800	-7.06054900	3.82015900
H	11.11857900	-6.98393600	4.29377900
C	9.06652500	-6.63485800	0.14380700
H	8.45757300	-6.17992300	-0.65068200
C	10.40346600	-7.05225400	-0.48348200
H	10.96682500	-6.19050600	-0.85640800
H	10.24314700	-7.73595300	-1.32521000
H	11.03401500	-7.57637300	0.24343000
C	8.30093700	-7.85738900	0.67232600
H	8.84668900	-8.35855400	1.47846500
H	8.15333000	-8.59418600	-0.12594400
H	7.31242600	-7.59068200	1.05953700
C	10.68996700	2.51213700	-2.27417300
C	10.86738100	3.63285100	-2.73134800
C	10.23192500	6.57784900	-2.39304800
H	10.98570400	6.83717900	-1.63554100
C	9.00611200	6.03372900	-1.64385200
H	8.22122100	5.70507800	-2.33393300
H	8.57233500	6.80781600	-0.99963200
H	9.27090700	5.18043100	-1.01278900

C	9.88395400	7.86509600	-3.15713000
H	10.75804400	8.31009100	-3.64319600
H	9.46856900	8.61888100	-2.47829300
H	9.13465600	7.68000700	-3.93448300
C	10.29004900	5.18508200	-5.22625500
H	10.42027000	6.17435300	-5.68904800
C	8.78538600	4.88822400	-5.14222400
H	8.60068100	3.94327000	-4.61871400
H	8.35119800	4.79997500	-6.14506100
H	8.23591400	5.67413300	-4.61541900
C	10.98653800	4.14344100	-6.11447800
H	12.03795500	4.38724800	-6.29376400
H	10.49403400	4.07582400	-7.09165100
H	10.94788000	3.14864800	-5.65622200
C	12.95173800	5.65791400	-3.59459500
H	13.19940100	6.08843400	-2.61348800
C	13.28454200	6.71491700	-4.65944900
H	13.05962000	6.35249300	-5.66844200
H	14.35086900	6.96775200	-4.63519700
H	12.72542300	7.64447400	-4.51247100
C	13.82251900	4.40476600	-3.77175600
H	13.66817500	3.69331000	-2.95535900
H	14.88568400	4.67271900	-3.78902500
H	13.60093100	3.88552300	-4.71068400
Si	-9.22578900	5.22448400	1.57194500
Si	-11.12069100	-5.16485200	-3.63568500
C	0.28670800	-2.63995400	5.17355600
H	0.32121200	-2.95561800	4.13542100
C	0.38606500	-3.57643700	6.20132900

H	0.50211100	-4.62835000	5.96079800
C	0.33326500	-3.17424500	7.53625500
H	0.40922200	-3.91642900	8.32450500
C	0.18104500	-1.83010000	7.86535900
H	0.13704500	-1.51911000	8.90479600
C	0.08628700	-0.89657600	6.83850400
C	0.14164300	-1.30045600	5.49974800
C	-4.81041600	-1.11227300	1.40541100
C	-5.77918400	-0.13802700	1.24546900
H	-5.63724000	0.84681300	1.68318700
C	-6.94808300	-0.38149900	0.49719200
C	-7.94900400	0.61337600	0.32914500
H	-7.80085400	1.58351600	0.79261500
C	-9.09721200	0.38329600	-0.39726200
C	-10.11002000	1.40531700	-0.55232700
C	-11.30699100	1.16292500	-1.32372300
C	-12.27160400	2.13544300	-1.48293900
H	-12.12621500	3.10534300	-1.01542300
C	-13.45275000	1.91017100	-2.24233100
C	-14.44244400	2.90453600	-2.40515900
H	-14.29713600	3.87264700	-1.93420200
C	-15.57352500	2.65220700	-3.14818000
H	-16.32807100	3.42302400	-3.26715500
C	-15.75788900	1.39490300	-3.75662600
H	-16.65328500	1.20562800	-4.33982800
C	-14.80791500	0.40911800	-3.61289400
H	-14.94550100	-0.56286400	-4.07857800
C	-13.63886000	0.64320000	-2.85598200
C	-12.63935900	-0.35371800	-2.68623100

H	-12.78040300	-1.32058200	-3.16055900
C	-11.49986200	-0.12955000	-1.94359600
C	-10.49757100	-1.15791900	-1.76964600
C	-9.29171200	-0.91016100	-1.01347200
C	-8.32034200	-1.87902600	-0.86278000
H	-8.46155200	-2.84759000	-1.33356400
C	-7.13651200	-1.64869000	-0.11191200
C	-6.13991700	-2.63508000	0.05525200
H	-6.28393000	-3.61140000	-0.39915300
C	-5.01024500	-2.37437900	0.79364500
H	-4.26706900	-3.15305400	0.93449400
C	-9.90938900	2.64881800	0.06740900
C	-9.68591300	3.71097200	0.63204900
C	-7.54891300	4.84963500	2.38352800
H	-7.26497300	5.74996000	2.94833200
C	-6.46632500	4.59230900	1.32440900
H	-6.72329800	3.72867700	0.70004300
H	-5.49941000	4.38225800	1.79673900
H	-6.32801700	5.44785200	0.65563700
C	-7.62723000	3.67646800	3.37056300
H	-8.30275000	3.88386000	4.20606100
H	-6.63945900	3.45740500	3.79371300
H	-7.98462100	2.76524700	2.87675700
C	-10.56357500	5.49842900	2.89164600
H	-10.38570700	4.69732600	3.62332700
C	-11.99687400	5.31960900	2.37070700
H	-12.71730600	5.38462400	3.19461400
H	-12.12949900	4.34904400	1.88362600
H	-12.26208100	6.09568100	1.64544200

C	-10.40187200	6.84006000	3.62110900
H	-10.63143100	7.68005000	2.95598900
H	-9.38571100	6.98686900	4.00347500
H	-11.08795700	6.90623700	4.47338200
C	-9.02940400	6.64035000	0.32180800
H	-8.42159100	6.20064300	-0.48207200
C	-10.36320600	7.07892400	-0.29761200
H	-10.92988900	6.22886300	-0.69171300
H	-10.19821100	7.78153800	-1.12270000
H	-10.99261700	7.58867900	0.44040400
C	-8.25819600	7.84622400	0.87953200
H	-8.80284500	8.33197400	1.69575700
H	-8.10481100	8.60013700	0.09852900
H	-7.27201000	7.56520500	1.26253300
C	-10.70439600	-2.42379900	-2.33957000
C	-10.88923500	-3.53112700	-2.82551200
C	-10.26953600	-6.48631500	-2.55875000
H	-11.02085500	-6.75778300	-1.80304400
C	-9.03615900	-5.96709300	-1.80431100
H	-8.25309400	-5.62749700	-2.49116300
H	-8.60364100	-6.75843900	-1.18054500
H	-9.29204700	-5.12696200	-1.15227800
C	-9.93410600	-7.75834300	-3.35330500
H	-10.81361600	-8.18657700	-3.84459500
H	-9.52034200	-8.52987000	-2.69367600
H	-9.18752000	-7.56093200	-4.13022800
C	-10.33438300	-5.02869200	-5.35928500
H	-10.47324500	-6.00622100	-5.84399600
C	-8.82747100	-4.74292500	-5.27704500

H	-8.63403100	-3.81142200	-4.73297800
H	-8.39837300	-4.63415500	-6.28006000
H	-8.27984900	-5.54406900	-4.77169500
C	-11.02929200	-3.96264300	-6.21937200
H	-12.08326500	-4.19561400	-6.39801000
H	-10.54198200	-3.87578300	-7.19762700
H	-10.98161800	-2.97883400	-5.73878100
C	-12.98998200	-5.52355200	-3.72412400
H	-13.23446800	-5.97554800	-2.75190500
C	-13.33502600	-6.55346900	-4.81140600
H	-13.11359800	-6.16892300	-5.81294600
H	-14.40270300	-6.80051500	-4.78692900
H	-12.78071200	-7.48948500	-4.68939500
C	-13.85463200	-4.26179700	-3.86676700
H	-13.69138000	-3.57039000	-3.03503300
H	-14.91938700	-4.52336600	-3.88378800
H	-13.63571800	-3.72211300	-4.79471500
C	-3.58765400	-0.84438400	2.19998100
C	-2.33972300	-1.32508900	1.79029300
C	-1.19506900	-1.06054700	2.52899800
C	-1.25281400	-0.30397600	3.70315800
C	-2.49796600	0.16942000	4.11720000
C	-3.64455300	-0.09629000	3.37896400
H	-2.25826000	-1.89008000	0.86649900
H	-0.23748900	-1.42857700	2.17389300
H	-2.57731800	0.74477200	5.03324600
H	-4.60371700	0.26568800	3.73705000
C	2.34174300	1.25838800	1.84760400
C	3.59127100	0.76251200	2.23347000

C	3.65154000	-0.03579700	3.37911700
C	2.50655300	-0.33780600	4.10533700
C	1.25936800	0.14899300	3.71320900
C	1.19850300	0.95852100	2.57529100
H	2.25728600	1.86325200	0.94968300
H	4.61196600	-0.41031100	3.72048400
H	2.58814800	-0.95358000	4.99456600
H	0.23963900	1.33992800	2.23823200

Table S15. The optimized S_0 structure of **PhTIPSPc** based on ωB97X-D/6-31G** level of theory.

Si	-5.19388300	-2.38683000	-0.00645400
Si	6.16131100	0.78692700	-0.17508700
C	1.49694100	-6.92215000	-0.00520300
C	0.50944300	-5.99017100	0.00238700
H	-0.53583300	-6.28555900	0.01827000
C	0.82140300	-4.58764300	-0.01032900
C	-0.16339600	-3.61791600	-0.00059200
H	-1.20819100	-3.91311500	0.01769100
C	0.14922500	-2.24202700	-0.01369600
C	-0.85988400	-1.25098700	-0.00315700
C	-0.52995800	0.12448100	-0.01754900
C	-1.52513100	1.12512700	-0.00648800
H	-2.56737500	0.82251600	0.02314900
C	-1.20423000	2.46941100	-0.03113800
C	-2.21305200	3.48833200	-0.02452700
H	-3.25314300	3.17687500	0.02255500
C	-1.89329400	4.81437700	-0.04677900
C	-0.50289500	5.19684400	-0.07236400
H	-0.25524100	6.25292500	-0.11415200
C	0.48574000	4.26897000	-0.07780200
H	1.52860200	4.57138700	-0.11097200
C	0.18203000	2.86528500	-0.05700700
C	1.16760800	1.89804800	-0.06412900
H	2.21192200	2.19468600	-0.07590800
C	0.85462700	0.52122900	-0.04985500
C	1.86326600	-0.46930800	-0.06809700
C	1.53360200	-1.84484200	-0.04233500
C	2.52884400	-2.84499300	-0.04634800

H	3.57130100	-2.54148300	-0.05569600
C	2.20833400	-4.18944300	-0.03188700
C	3.21724300	-5.21240600	-0.03883500
H	4.25975600	-4.90735900	-0.05631700
C	2.87498100	-6.52641100	-0.02624000
H	3.64491500	-7.29122700	-0.03252600
C	-2.22922900	-1.63817100	0.01490100
C	-3.40615500	-1.95060500	0.02465400
C	-5.30174000	-4.25058200	-0.34768600
H	-6.37124100	-4.50534500	-0.38406100
C	-4.65737600	-5.05650900	0.79021800
H	-3.59496100	-4.80572900	0.89377300
H	-4.72457000	-6.13293800	0.59373100
H	-5.13460500	-4.86662100	1.75682200
C	-4.67450300	-4.62883700	-1.69664100
H	-5.18100600	-4.14624200	-2.53807900
H	-4.72547000	-5.71174600	-1.86000300
H	-3.61818100	-4.33824000	-1.73399300
C	-5.97767100	-1.39241100	-1.42397100
H	-5.63165500	-1.90058000	-2.33532300
C	-5.48920700	0.06124100	-1.50913300
H	-5.91980500	0.56165600	-2.38461300
H	-4.39990700	0.11343400	-1.59516200
H	-5.78365300	0.63914600	-0.62664500
C	-7.51184400	-1.46086700	-1.40751200
H	-7.92201500	-0.90673400	-0.55594800
H	-7.88245700	-2.48988600	-1.34385500
H	-7.93219500	-1.01443700	-2.31607900
C	-5.89228600	-2.00522500	1.71764000

H	-5.16669500	-2.46623800	2.40329900
C	-5.92568200	-0.50261500	2.02826300
H	-4.95716000	-0.02619500	1.84361000
H	-6.18882600	-0.32661900	3.07771600
H	-6.67558400	0.01135400	1.41648000
C	-7.25875600	-2.65474200	1.98363900
H	-8.03612900	-2.24076200	1.33340500
H	-7.57426000	-2.47823800	3.01860400
H	-7.23972300	-3.73794000	1.82628500
C	3.22992900	-0.07584800	-0.12228300
C	4.39772400	0.26430700	-0.18172500
C	7.17953100	-0.54746000	-1.07642300
H	7.11066800	-0.28066300	-2.14101100
C	6.60788300	-1.96551300	-0.92733700
H	6.59594200	-2.29069700	0.11875600
H	7.21450600	-2.68711200	-1.48745000
H	5.58264100	-2.02375100	-1.30412300
C	8.66566400	-0.50890800	-0.68660400
H	9.11339000	0.47675600	-0.84884800
H	9.24081900	-1.23036100	-1.27843100
H	8.81046900	-0.76567500	0.36846400
C	6.66140100	0.96345700	1.64823300
H	7.71582300	1.27635300	1.65419800
C	6.55190900	-0.37135400	2.40022600
H	5.52912100	-0.76266200	2.35761500
H	6.81236300	-0.24314600	3.45744400
H	7.21850100	-1.13526800	1.98884100
C	5.83573900	2.04367800	2.36278800
H	5.98245500	3.03553600	1.92479700

H	6.11448500	2.10769100	3.42117400
H	4.76523100	1.81340000	2.31709200
C	6.29483300	2.43573900	-1.12069000
H	6.39052100	2.14618600	-2.17720400
C	7.55918100	3.22300400	-0.74178200
H	7.53312500	3.54370800	0.30536300
H	7.65250900	4.12610200	-1.35600500
H	8.47279300	2.63669400	-0.88279600
C	5.04201900	3.31728400	-1.00492000
H	4.15363200	2.79837000	-1.37630100
H	5.16274800	4.23818500	-1.58783000
H	4.84599300	3.61037000	0.03240100
H	1.25023800	-7.97894200	0.00449500
C	-4.11714800	5.69985500	-0.79026400
C	-2.94584800	5.85967600	-0.04128800
C	-2.79399900	7.02488700	0.71893300
C	-3.78758500	7.99711900	0.73668300
C	-4.95042100	7.82372200	-0.00830100
C	-5.11135200	6.67128700	-0.77200000
H	-4.23619100	4.81571100	-1.40934700
H	-1.90033300	7.15919900	1.32125500
H	-3.65550000	8.88999300	1.33963600
H	-5.72520500	8.58355300	0.00395100
H	-6.01010200	6.53187900	-1.36450600

Table S16. The optimized S_1 structure of **PhTIPSPc** based on ωB97X-D/6-31G** level of theory.

Si	-5.16687600	-2.41060600	-0.01407800
Si	6.14903300	0.84349600	-0.17992200
C	1.60362900	-6.94060200	0.00158600
C	0.59606800	-6.00234400	0.00524100
H	-0.44536200	-6.31153400	0.01633300
C	0.89709300	-4.62050800	-0.00515600
C	-0.11066600	-3.63103000	-0.00040100
H	-1.15148300	-3.94176500	0.01175100
C	0.18311800	-2.27313100	-0.01085200
C	-0.84425000	-1.27976700	-0.00163000
C	-0.53281200	0.11569300	-0.01155300
C	-1.52533500	1.08749700	0.00324700
H	-2.56542100	0.77636200	0.03222900
C	-1.22827600	2.46880700	-0.01995000
C	-2.24095600	3.45041100	-0.01576000
H	-3.27749600	3.12668700	0.02745700
C	-1.94021100	4.80187800	-0.03707800
C	-0.57801500	5.19441100	-0.05838400
H	-0.33381100	6.25120800	-0.10049000
C	0.43092600	4.26061700	-0.06102300
H	1.46981200	4.57719000	-0.09009500
C	0.13905700	2.87738600	-0.04320600
C	1.14646600	1.89030200	-0.05061700
H	2.18689600	2.20183600	-0.06120100
C	0.85227300	0.53082300	-0.04164500
C	1.87822700	-0.46161700	-0.06206800
C	1.56768400	-1.85771100	-0.03477300
C	2.56085700	-2.82841600	-0.03274800

H	3.60094700	-2.51531800	-0.03809500
C	2.26471700	-4.20982200	-0.02001100
C	3.27763500	-5.19639100	-0.02338300
H	4.31680200	-4.87953300	-0.03613600
C	2.95423700	-6.53477800	-0.01290600
H	3.74013800	-7.28300700	-0.01608900
C	-2.19757400	-1.67653800	0.01179700
C	-3.37738600	-1.99391300	0.01673400
C	-5.30577300	-4.25701800	-0.43093200
H	-6.37940200	-4.49420800	-0.46630300
C	-4.66231100	-5.11893800	0.66559000
H	-3.59542900	-4.88738300	0.76820800
H	-4.74680600	-6.18524300	0.42517100
H	-5.12707600	-4.96294900	1.64426300
C	-4.69781900	-4.58777700	-1.80094700
H	-5.20445300	-4.06174100	-2.61587000
H	-4.76772900	-5.66188000	-2.00958900
H	-3.63747500	-4.31200700	-1.83633400
C	-5.95836500	-1.34912900	-1.37948500
H	-5.63593100	-1.82681700	-2.31552900
C	-5.44731600	0.09869900	-1.41707600
H	-5.89108700	0.64254500	-2.25959100
H	-4.35978700	0.13534400	-1.52990900
H	-5.70862500	0.64404900	-0.50376500
C	-7.49299700	-1.39359200	-1.34010400
H	-7.88062500	-0.86419900	-0.46271400
H	-7.87953300	-2.41818500	-1.30745600
H	-7.92020600	-0.90794500	-2.22513800
C	-5.84673200	-2.09226700	1.73166200

H	-5.12089900	-2.59012500	2.39066600
C	-5.85665100	-0.60324500	2.10314600
H	-4.88268200	-0.13387600	1.92928000
H	-6.10874200	-0.46609000	3.16113300
H	-6.60399800	-0.05430100	1.51914700
C	-7.21901400	-2.73459400	1.98457800
H	-7.99747600	-2.28365100	1.36087500
H	-7.52149500	-2.59786400	3.02948700
H	-7.21602900	-3.81037300	1.78173600
C	3.22796400	-0.05601200	-0.11999600
C	4.39520400	0.29908200	-0.18267400
C	7.18472800	-0.47949200	-1.07927600
H	7.10844100	-0.21719500	-2.14447600
C	6.63335900	-1.90477200	-0.92376100
H	6.63032200	-2.22700100	0.12334600
H	7.24719700	-2.61984800	-1.48451500
H	5.60726200	-1.97730000	-1.29592500
C	8.67138900	-0.41872900	-0.69464300
H	9.10460000	0.57250200	-0.86228200
H	9.25477900	-1.13435300	-1.28561600
H	8.82339700	-0.66918300	0.36093800
C	6.65640700	1.03364800	1.64132700
H	7.70643100	1.36130900	1.64050600
C	6.56912100	-0.29970800	2.39845000
H	5.55170000	-0.70536500	2.36103400
H	6.83208100	-0.16427400	3.45421800
H	7.24476400	-1.05556700	1.98685500
C	5.81887800	2.10461200	2.35571600
H	5.94893600	3.09671400	1.91296600

H	6.10164500	2.17709700	3.41255300
H	4.75168000	1.85831500	2.31567000
C	6.26207000	2.49220300	-1.12969200
H	6.36049500	2.20057100	-2.18537900
C	7.51637800	3.29696900	-0.75445600
H	7.48705800	3.62095800	0.29158000
H	7.59775000	4.19910700	-1.37189800
H	8.43732400	2.72191500	-0.89406600
C	4.99805600	3.35767400	-1.01544600
H	4.11614300	2.82489300	-1.38276500
H	5.10528700	4.27773900	-1.60242800
H	4.80020800	3.65243400	0.02112000
H	1.36041800	-7.99807500	0.00972500
C	-4.18592000	5.62388100	-0.78425300
C	-3.01735500	5.82044900	-0.03897800
C	-2.89203600	6.99865100	0.70626000
C	-3.90557500	7.95004000	0.70852300
C	-5.06370900	7.74149200	-0.03489600
C	-5.19996400	6.57451200	-0.78123300
H	-4.28788200	4.72832500	-1.38987100
H	-2.00302000	7.15820100	1.30917100
H	-3.79355000	8.85355900	1.29953200
H	-5.85480800	8.48439900	-0.03336100
H	-6.09554500	6.40706700	-1.37123500

Section 5. ¹H and ¹³C NMR spectra

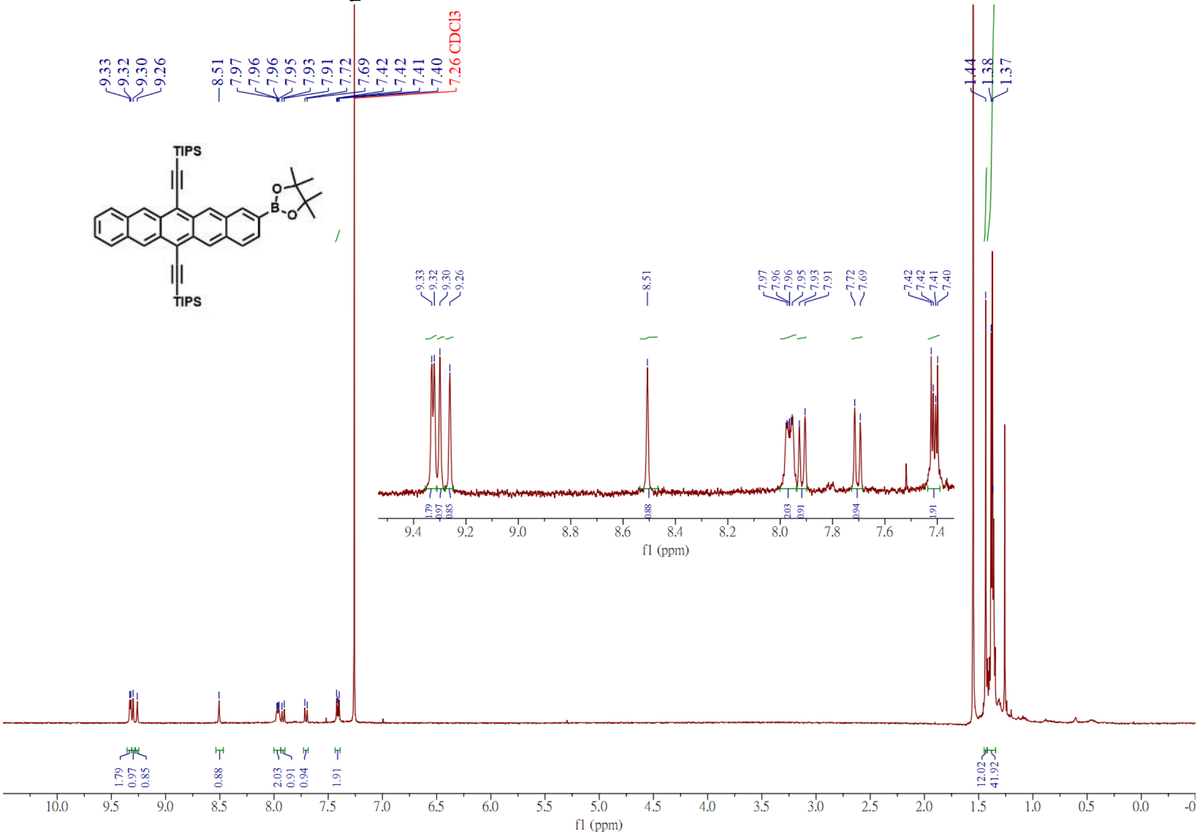

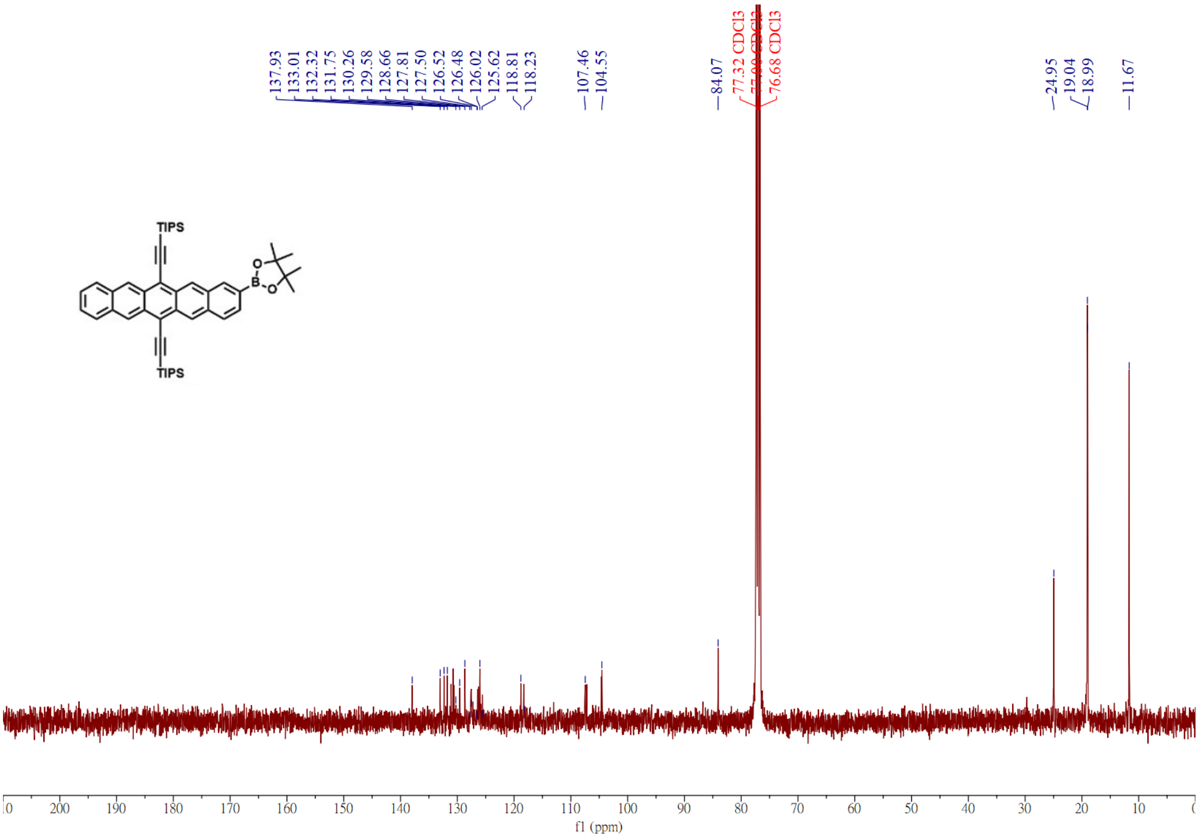

Figure S25. ¹H and ¹³C NMR spectra of intermediate **2**.

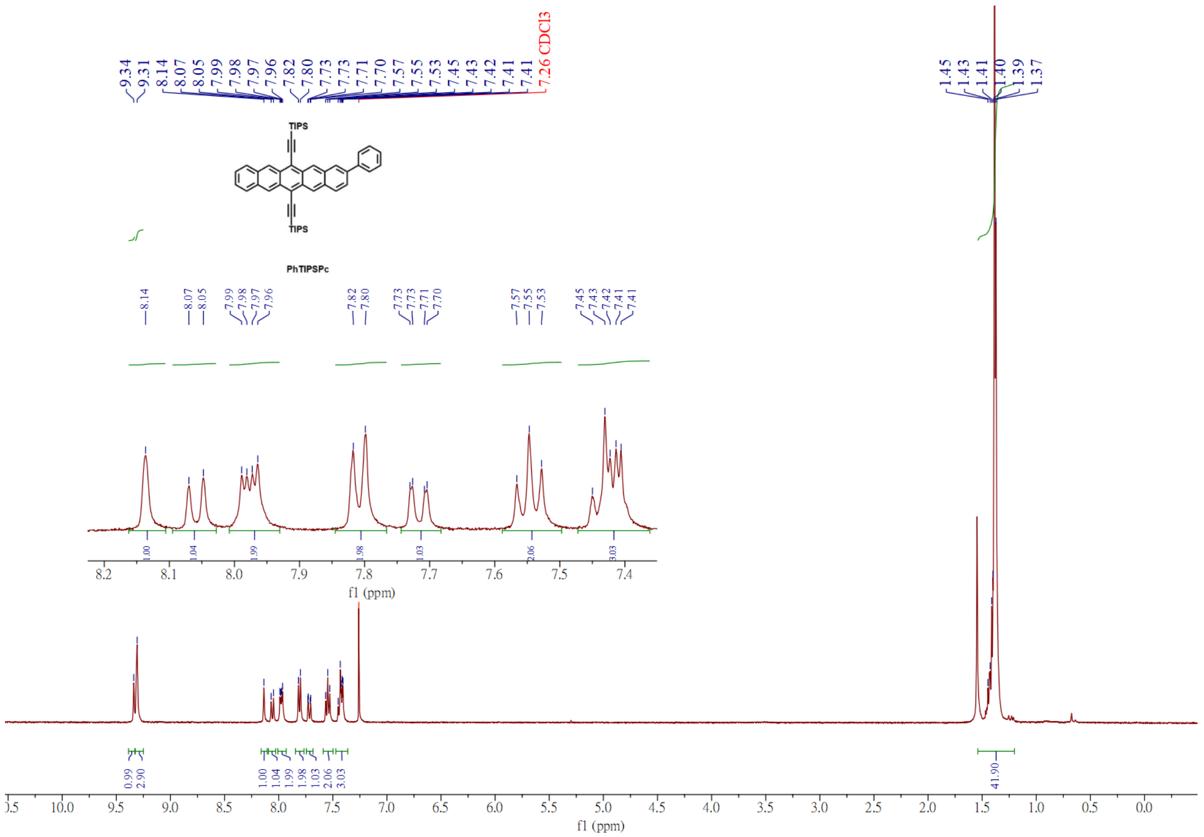

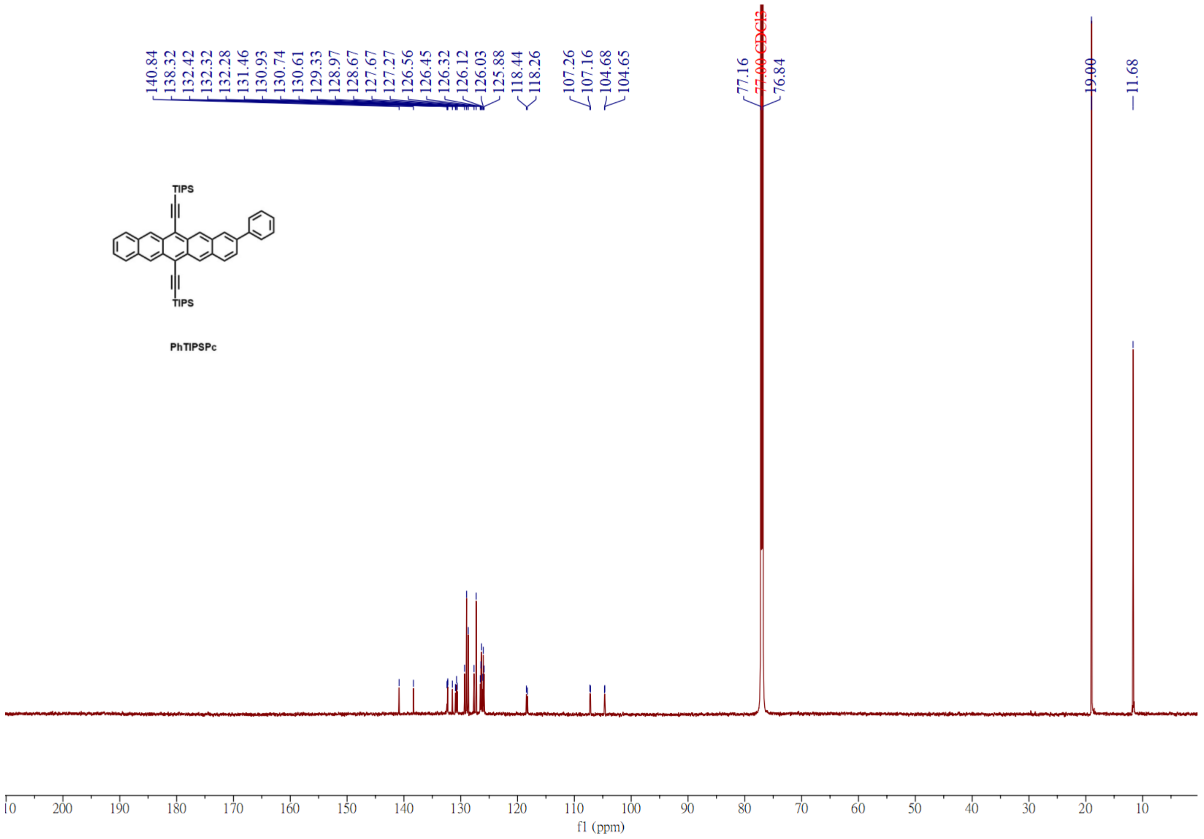

Figure S26. ^{1}H and ^{13}C NMR spectra of **PhTIPSPc**.

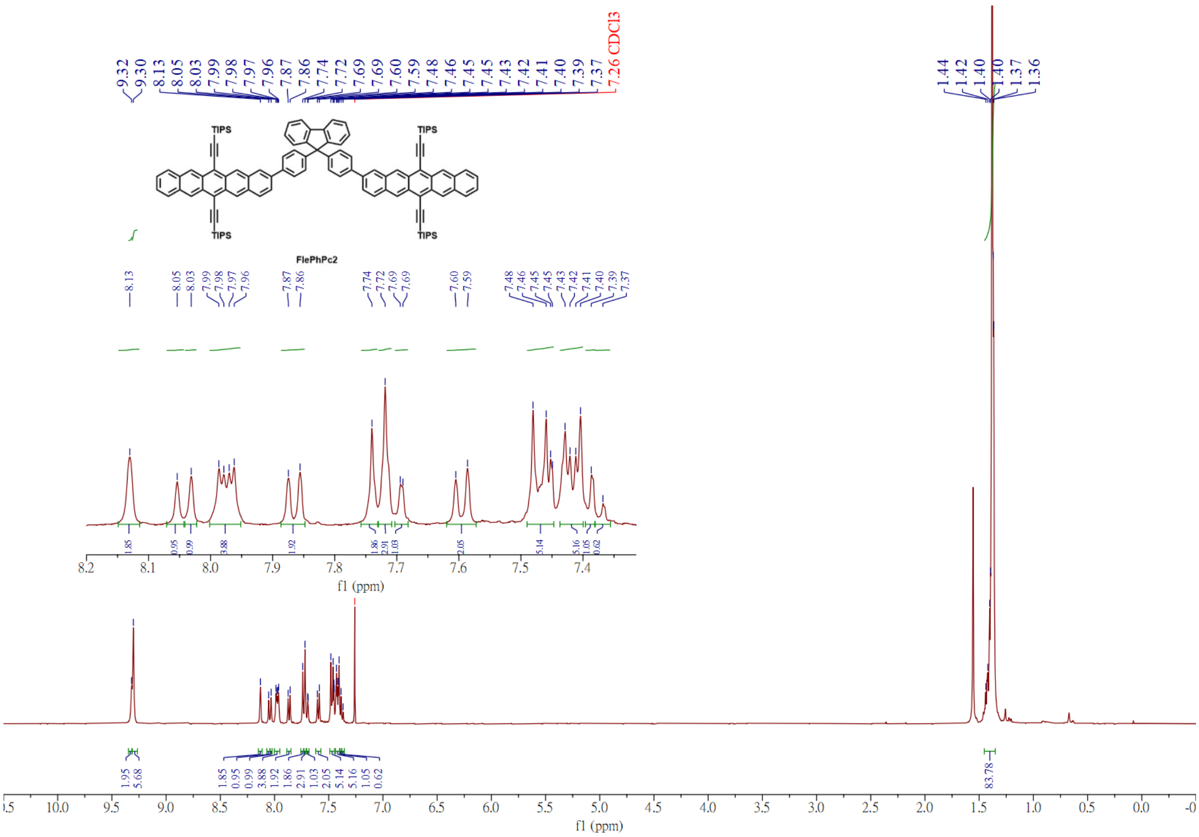

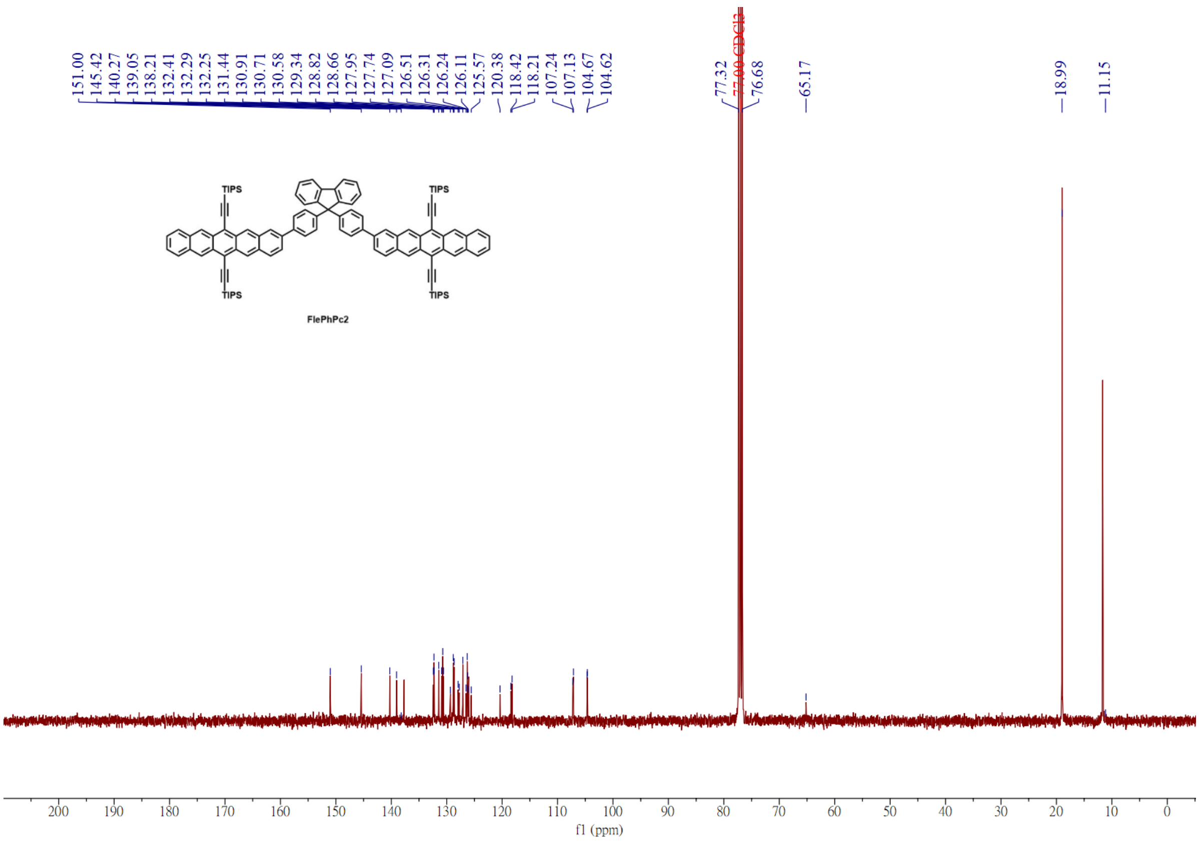

Figure S27. ^{1}H and ^{13}C NMR spectra of **FlePhPc2**.

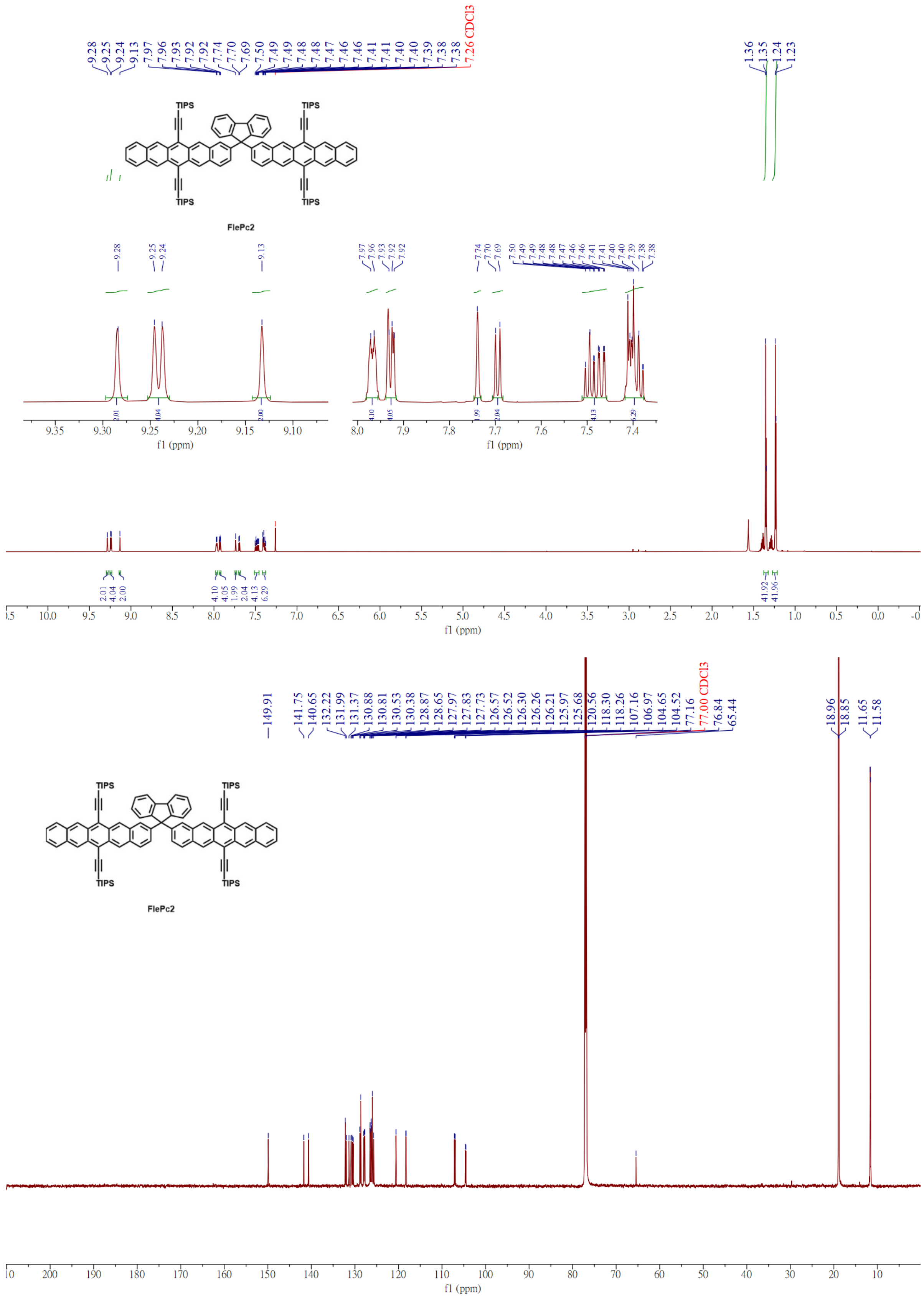

Figure S28. ^{1}H and ^{13}C NMR spectra of **FlePc2**.

Section 6. X-ray Crystallography

Table S17. Crystal data and experimental details for **FlePc2**.

	Crystal data	
Empirical formula	C101 H114 Si4	
Formula weight	1440.28	
Crystal system	Monoclinic	
Space group	C2/c	
Unit cell dimensions	a = 28.1989(13) Å	a= 90°.
	b = 11.7190(6) Å	b= 105.0179(18)°.
	c = 26.6375(13) Å	g = 90°.
Volume	8502.0(7) Å^3	
Z	4	
F(000)	3104	
Density (calculated)	1.125 Mg/m^3	
Wavelength	1.54178 Å	
Cell parameters reflections used	9934	
Theta range for Cell parameters	3.44° to 69.99°.	
Absorption coefficient	0.990 mm^{-1}	
Temperature	100(2) K	
Crystal size	0.150 x 0.100 x 0.050 mm^3	
	Data collection	
Diffractometer	Bruker AXS D8 VENTURE, PhotonIII_C28	
Absorption correction	Semi-empirical from equivalents	
Max. and min. transmission	0.7533 and 0.6388	
No. of measured reflections	44756	
No. of independent reflections	8038 [R(int) = 0.0609]	

No. of observed [I>2_igma(I)]	6997
Completeness to theta = 67.679°	99.8 %
Theta range for data collection	3.245° to 70.091°
	Refinement
Final R indices [I>2sigma(I)]	R1 = 0.0563, wR2 = 0.1553
R indices (all data)	R1 = 0.0636, wR2 = 0.1628
Goodness-of-fit on F2	1.042
No. of reflections	8038
No. of parameters	486
No. of restraints	27
Largest diff. peak and hole	1.040 and -0.586 e·Å^{-3}

Section 7. Reference

(1) Brackmann, U. L. L. D., 2nd ed.; Lambda Physik: Göttingen, Germany, 1997.There is no corresponding record for this reference.

(2) Wei, Y. C.; Chen, B. H.; Ye, R. S.; Huang, H. W.; Su, J. X.; Lin, C. Y.; Hodgkiss, J.; Hsu, L. Y.; Chi, Y.; Chen, K. Excited-State THz Vibrations in Aggregates of PtII Complexes Contribute to the Enhancement of Near-Infrared Emission Efficiencies. *Angew. Chem. Int. Ed.* **2023**, *62* (16), e202300815.

(3) Jaumot, J.; de Juan, A.; Tauler, R. MCR-ALS GUI 2.0: New features and applications. *Chemom. Intell. Lab. Syst.* **2015**, *140*, 1-12.

(4) Šimėnas, M.; O'Sullivan, J.; Zollitsch, C. W.; Kennedy, O.; Seif-Eddine, M.; Ritsch, I.; Hülsmann, M.; Qi, M.; Godt, A.; Roessler, M. M. A sensitivity leap for X-band EPR using a probehead with a cryogenic preamplifier. *J. Magn. Reson.* **2021**, *322*, 106876.

(5) Cheng, C.-C.; Tsai, R.-F.; Lin, C.-K.; Tan, K.-T.; Kalendra, V.; Simenas, M.; Lin, C.-W.; Chiang, Y.-W. In-Cell DEER Spectroscopy of Nanodisc-Delivered Membrane Proteins in Living Cell Membranes. *JACS Au* **2024**, *4* (10), 3766-3770.

(6) Wu, Y.-S.; Chu, L.-K.; Yu, T.-Y. Investigation of Methionine Metabolism in Coccolithophore by In Situ Light-Coupled Nuclear Magnetic Resonance Spectroscopy. *J. Phys. Chem. Lett.* **2025**, *16*, 5800-5805.

(7) Feng, X.; Casanova, D.; Krylov, A. I. Intra-and intermolecular singlet fission in covalently linked dimers. *J. Phys. Chem. C* **2016**, *120* (34), 19070-19077.

(8) Han, J.; Rehn, D. R.; Buckup, T.; Dreuw, A. Evaluation of single-reference DFT-based approaches for the calculation of spectroscopic signatures of excited states involved in singlet fission. *J. Phys. Chem. A* **2020**, *124* (41), 8446-8460.

(9) Feng, X.; Krylov, A. I. On couplings and excimers: lessons from studies of singlet fission in covalently linked tetracene dimers. *Phys. Chem. Chem. Phys.* **2016**, *18* (11), 7751-7761.

(10) Korovina, N. V.; Das, S.; Nett, Z.; Feng, X.; Joy, J.; Haiges, R.; Krylov, A. I.; Bradforth, S. E.; Thompson, M. E. Singlet fission in a covalently linked cofacial alkynyltetracene dimer. *J. Am. Chem. Soc.* **2016**, *138* (2), 617-627.

(11) Feng, X.; Luzanov, A. V.; Krylov, A. I. Fission of entangled spins: An electronic structure perspective. *J. Phys. Chem. Lett.* **2013**, *4* (22), 3845-3852.

(12) Matsika, S.; Feng, X.; Luzanov, A. V.; Krylov, A. I. What we can learn from the norms of one-particle density matrices, and what we can't: Some results for interstate properties in model singlet fission systems. *J. Phys. Chem. A* **2014**, *118* (51), 11943-11955.

(13) Yang, C.-H.; Hsu, C.-P. First-principle characterization for singlet fission couplings. *J. Phys. Chem. Lett.* **2015**, *6* (10), 1925-1929.

(14) Lin, H.-H.; Kue, K. Y.; Claudio, G. C.; Hsu, C.-P. First principle prediction of intramolecular singlet fission and triplet triplet annihilation rates. *J. Chem. Theory Comput.* **2019**, *15* (4), 2246-2253.

(15) Tilley, A. J.; Pensack, R. D.; Kynaston, E. L.; Scholes, G. D.; Seferos, D. S. Singlet Fission in Core–Shell Micelles of End-Functionalized Polymers. *Chem. Mater.* **2018**, *30* (13), 4409-4421.

(16) Su, T.-H.; Fan, C.-H.; Ou-Yang, Y.-H.; Hsu, L.-C.; Cheng, C.-H. Highly efficient deep-red organic electrophosphorescent devices with excellent operational stability using bis (indoloquinoxalinyl) derivatives as the host materials. *J. Mater. Chem. C* **2013**, *1* (33), 5084-5092.

(17) Cao, X.; Yang, W.; Liu, C.; Wei, F.; Wu, K.; Sun, W.; Song, J.; Xie, L.; Huang, W. Palladium-catalyzed direct arylation of C–H bond to construct quaternary carbon centers: the synthesis of diarylfluorene. *Org. Lett.* **2013**, *15* (12), 3102-3105.

(18) Kato, D.; Sakai, H.; Tkachenko, N. V.; Hasobe, T. High-Yield Excited Triplet States in Pentacene Self-Assembled Monolayers on Gold Nanoparticles through Singlet Exciton Fission. *Angew. Chem. Int. Ed.* **2016**, *55* (17), 5230-5234.

(19) Kinoshita, T.; Nakamura, S.; Harada, M.; Hasobe, T.; Fukuhara, G. Control of intramolecular singlet fission in a pentacene dimer by hydrostatic pressure. *Chem. Sci.* **2023**, *14* (12), 3293-3301.

(20) Lukman, S.; Musser, A. J.; Chen, K.; Athanasopoulos, S.; Yong, C. K.; Zeng, Z.; Ye, Q.; Chi, C.; Hodgkiss, J. M.; Wu, J. Tuneable singlet exciton fission and triplet–triplet annihilation in an orthogonal pentacene dimer. *Adv. Funct. Mater.* **2015**, *25* (34), 5452-5461.

(21) Basel, B. S.; Zirzlmeier, J.; Hetzer, C.; Phelan, B. T.; Krzyaniak, M. D.; Reddy, S. R.; Coto, P. B.; Horwitz, N. E.; Young, R. M.; White, F. J. Unified model for singlet fission within a non-conjugated covalent pentacene dimer. *Nat. Commun.* **2017**, *8* (1), 15171.

(22) Halasinski, T. M.; Hudgins, D. M.; Salama, F.; Allamandola, L. J.; Bally, T. Electronic absorption spectra of neutral pentacene (C22H14) and its positive and negative ions in Ne, Ar, and Kr matrices. *J. Phys. Chem. A* **2000**, *104* (32), 7484-7491.

(23) Mondal, R.; Tönshoff, C.; Khon, D.; Neckers, D. C.; Bettinger, H. F. Synthesis, stability, and photochemistry of pentacene, hexacene, and heptacene: A matrix isolation study. *J. Am. Chem. Soc.* **2009**, *131* (40), 14281-14289.

(24) Wilson, M. W.; Rao, A.; Clark, J.; Kumar, R. S. S.; Brida, D.; Cerullo, G.; Friend, R. H. Ultrafast dynamics of exciton fission in polycrystalline pentacene. *J. Am. Chem. Soc.* **2011**, *133* (31), 11830-11833.
