# Supplementary material for: Single-Carbon Bridged Pentacene Dimers Enable Efficient Singlet Fission and Quintet State Stabilization
Source: J Am Chem Soc. 2026 Jan 23;148(4):4062–73. doi: 10.1021/jacs.5c14851 (PMC12879938; doi:10.1021/jacs.5c14851)
Supplement: Supplementary file 2 [file ja5c14851_si_002.pdf]

No syntax errors found.
Please wait while processing ....

CIF dictionary
Interpreting this report

Datablock: ic22955

Bond precision:	C-C = 0.0031 A		Wavelength=1.54178
Cell:	a=28.1989(13)	b=11.7190(6)	c=26.6375(13)
	alpha=90	beta=105.0179(18)	gamma=90
Temperature:	100 K		

	Calculated	Reported
Volume	8502.1(7)	8502.0(7)
Space group	C 2/c	C 2/c
Hall group	-C 2yc	-C 2yc
Moiety formula	C101 H114 Si4	?
Sum formula	C101 H114 Si4	C101 H114 Si4
Mr	1440.28	1440.28
Dx,g cm-3	1.125	1.125
Z	4	4
Mu (mm-1)	0.990	0.990
F000	3104.0	3104.0
F000'	3115.01	
h,k,lmax	34,14,32	34,14,32
Nref	8094	8038
Tmin,Tmax	0.888,0.952	0.639,0.753
Tmin'	0.862	

Correction method= # Reported T Limits: Tmin=0.639 Tmax=0.753 AbsCorr = MULTI-SCAN

Data completeness= 0.993 Theta(max)= 70.091

R(reflections)= 0.0563(6997) wR2(reflections)= 0.1628(8038)

S = 1.042 Npar= 486

The following ALERTS were generated. Each ALERT has the format
test-name_ALERT_alert-type_alert-level.
Click on the hyperlinks for more details of the test.

Alert level C

```
PLAT213_ALERT_2_C Atom C45              has ADP max/min Ratio .....        3.4 prolat
PLAT220_ALERT_2_C NonSolvent   Resd 1   C   Ueq(max)/Ueq(min) Range        4.3 Ratio
PLAT222_ALERT_3_C NonSolvent Resd 1  H   Uiso(max)/Uiso(min) Range        5.1 Ratio
PLAT911_ALERT_3_C Missing FCF Refl Between Thmin & STh/L=    0.600         14 Report
                 0 14  0,   2 14  0,  -2 14  1,   0  6  1,   0 14  1,   2 14  1,
                -2 14  2,   0 14  2,   4  0  2,  -3 11  3, -32  0  4,   2  2  5,
                 5 13  7,  -6  0  8,
```

Alert level G

```
PLAT003_ALERT_2_G Number of Uiso or U(i,j)  Restrained non-H-Atoms           3 Report
PLAT083_ALERT_2_G SHELXL Second Parameter in WGHT  Unusually Large       11.98 Why ?
PLAT178_ALERT_4_G The CIF-Embedded .res File Contains SIMU Records           1 Report
PLAT187_ALERT_4_G The CIF-Embedded .res File Contains RIGU Records           1 Report
PLAT188_ALERT_3_G A Non-default SIMU Restraint Value has been used      0.0100 Report
PLAT190_ALERT_3_G A Non-default RIGU Restraint Value for First Par      0.0010 Report
PLAT230_ALERT_2_G Hirshfeld Test Diff for    Si1       --C31      .        5.3 s.u.
PLAT230_ALERT_2_G Hirshfeld Test Diff for    Si2       --C42      .        5.9 s.u.
PLAT333_ALERT_2_G Large Aver C6-Ring C-C Dist C12       -C25      .       1.43 Ang.
PLAT371_ALERT_2_G Long   C(sp2)-C(sp1) Bond  C13       - C30      .       1.43 Ang.
PLAT371_ALERT_2_G Long   C(sp2)-C(sp1) Bond  C24       - C41      .       1.43 Ang.
PLAT802_ALERT_4_G CIF Input Record(s) with more than 80 Characters           1 Info
PLAT860_ALERT_3_G Number of Least-Squares Restraints ............          27 Note
PLAT883_ALERT_1_G Absent Datum for _atom_sites_solution_primary ..     Please Do !
PLAT912_ALERT_4_G Missing # of FCF Reflections Above STh/L=  0.600          42 Note
```

```
PLAT965_ALERT_2_G The SHELXL WEIGHT Optimisation has not Converged     Please Check
PLAT969_ALERT_5_G The 'Henn et al.' R-Factor-gap value ...........      3.155 Note
              Predicted wR2: Based on SigI**2  5.16 or SHELX Weight 15.63
PLAT978_ALERT_2_G Number C-C Bonds with Positive Residual Density.          8 Info
```

```
   0 ALERT level A = Most likely a serious problem - resolve or explain
   0 ALERT level B = A potentially serious problem, consider carefully
   4 ALERT level C = Check. Ensure it is not caused by an omission or oversight
  18 ALERT level G = General information/check it is not something unexpected

   1 ALERT type 1 CIF construction/syntax error, inconsistent or missing data
  11 ALERT type 2 Indicator that the structure model may be wrong or deficient
   5 ALERT type 3 Indicator that the structure quality may be low
   4 ALERT type 4 Improvement, methodology, query or suggestion
   1 ALERT type 5 Informative message, check
```

It is advisable to attempt to resolve as many as possible of the alerts in all categories. Often the minor alerts point to easily fixed oversights, errors and omissions in your CIF or refinement strategy, so attention to these fine details can be worthwhile. In order to resolve some of the more serious problems it may be necessary to carry out additional measurements or structure refinements. However, the purpose of your study may justify the reported deviations and the more serious of these should normally be commented upon in the discussion or experimental section of a paper or in the "special_details" fields of the CIF. checkCIF was carefully designed to identify outliers and unusual parameters, but every test has its limitations and alerts that are not important in a particular case may appear. Conversely, the absence of alerts does not guarantee there are no aspects of the results needing attention. It is up to the individual to critically assess their own results and, if necessary, seek expert advice.

Publication of your CIF in IUCr journals

A basic structural check has been run on your CIF. These basic checks will be run on all CIFs submitted for publication in IUCr journals (*Acta Crystallographica*, *Journal of Applied Crystallography*, *Journal of Synchrotron Radiation*); however, if you intend to submit to *Acta Crystallographica Section C* or *E* or *IUCrData*, you should make sure that full publication checks are run on the final version of your CIF prior to submission.

Publication of your CIF in other journals

Please refer to the *Notes for Authors* of the relevant journal for any special instructions relating to CIF submission.

PLATON version of 04/06/2025; check.def file version of 30/05/2025

Datablock ic22955 - ellipsoid plot

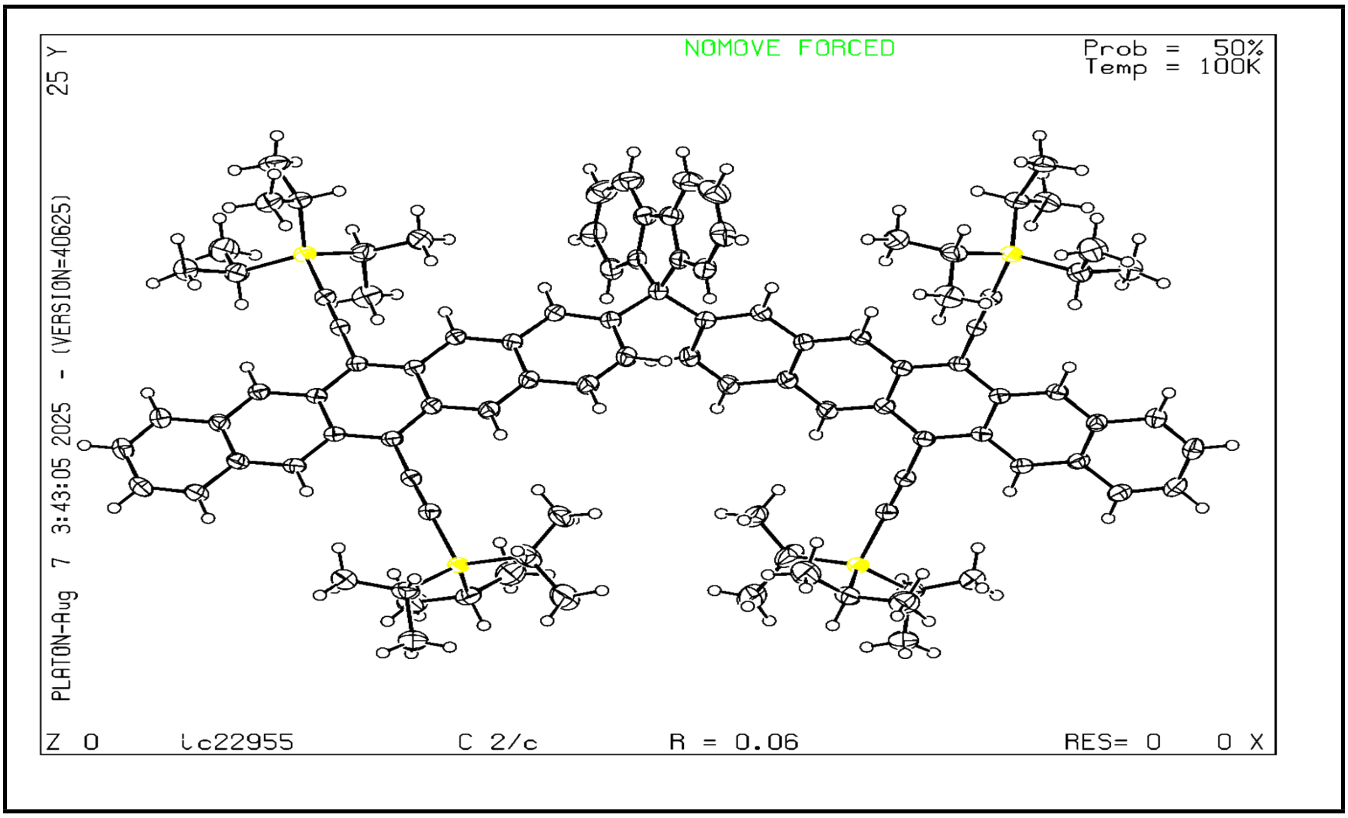

Download CIF editor (publCIF) from the IUCr
Download CIF editor (enCIFer) from the CCDC
Test a new CIF entry
